# Supplementary material for: Semiconductor photocatalysis to engineering deuterated N-alkyl pharmaceuticals enabled by synergistic activation of water and alkanols
Source: Nat Commun. 2020 Sep 18;11:4722. doi: 10.1038/s41467-020-18458-w (PMC7501254; doi:10.1038/s41467-020-18458-w)
Supplement: Supplementary file 1 — Supplementary information [file 41467_2020_18458_MOESM1_ESM.pdf]

Supplementary Information for

Semiconductor Photocatalysis to Engineering Deuterated *N*-alkyl Pharmaceuticals Enabled by Synergistic Activation of Water and Alkanols

Zhang et al

This file includes:

Supplementary Figure 1. Deuterated *N*-alkyl containing drugs.

Supplementary Figure 2. Comparison of diverse strategies for synthesis of deuterated *N*-alkyl amines.

Supplementary Discussion

Supplementary Table 1. Optimization of the Reaction Conditions and screening of reaction conditions for the reaction of *p*-toluidine.

Supplementary Figure 3. TEM images of Pd/CPCN photocatalyst.

Supplementary Figure 4. UV-Vis spectra of CPCN and Pd/CPCN photocatalyst.

Supplementary Figure 5. XRD patterns of CPCN and Pd/CPCN photocatalyst.

Supplementary Figure 6. Time-dependent yield of alkylation products.

Supplementary Methods: Gram scale synthesis of Loxapine-d3 (**4j**), Gram scale synthesis of Dofetilide-d3 (**4n**).

Supplementary Characterization of Products.

Supplementary Figure 7. Gram scale synthesis of Loxapine-d3 (**4j**).

Supplementary Figure 8. Synthesis of **4m**.

Supplementary Figure 9. Synthesis of **3da**.

Supplementary Figure 10. Synthesis of **4n**.

Supplementary Figure 11-Figure 134. ^{1}H, and ^{13}C NMR Spectra of products.

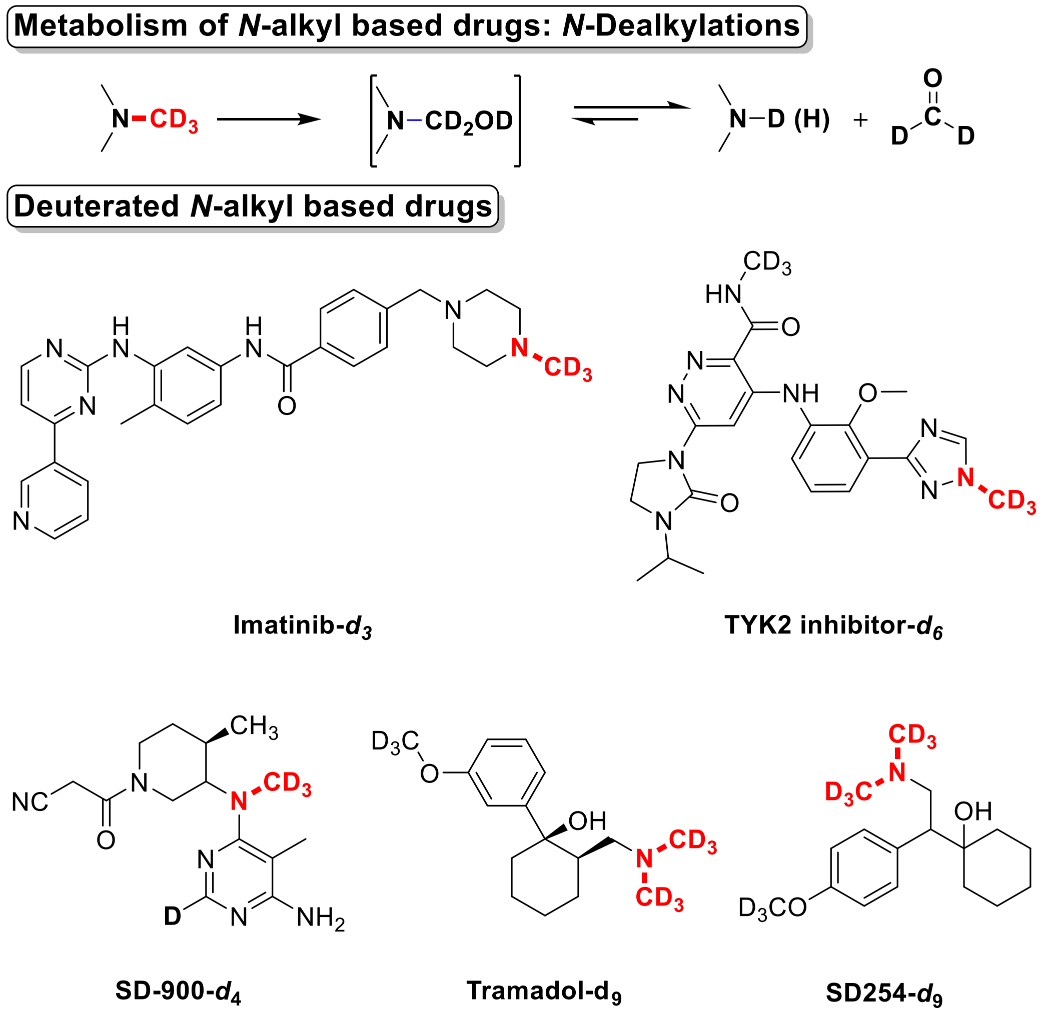

Supplementary Figure 1. *N*-Dealkylation process and typical deuterated *N*-alkyl containing drugs.

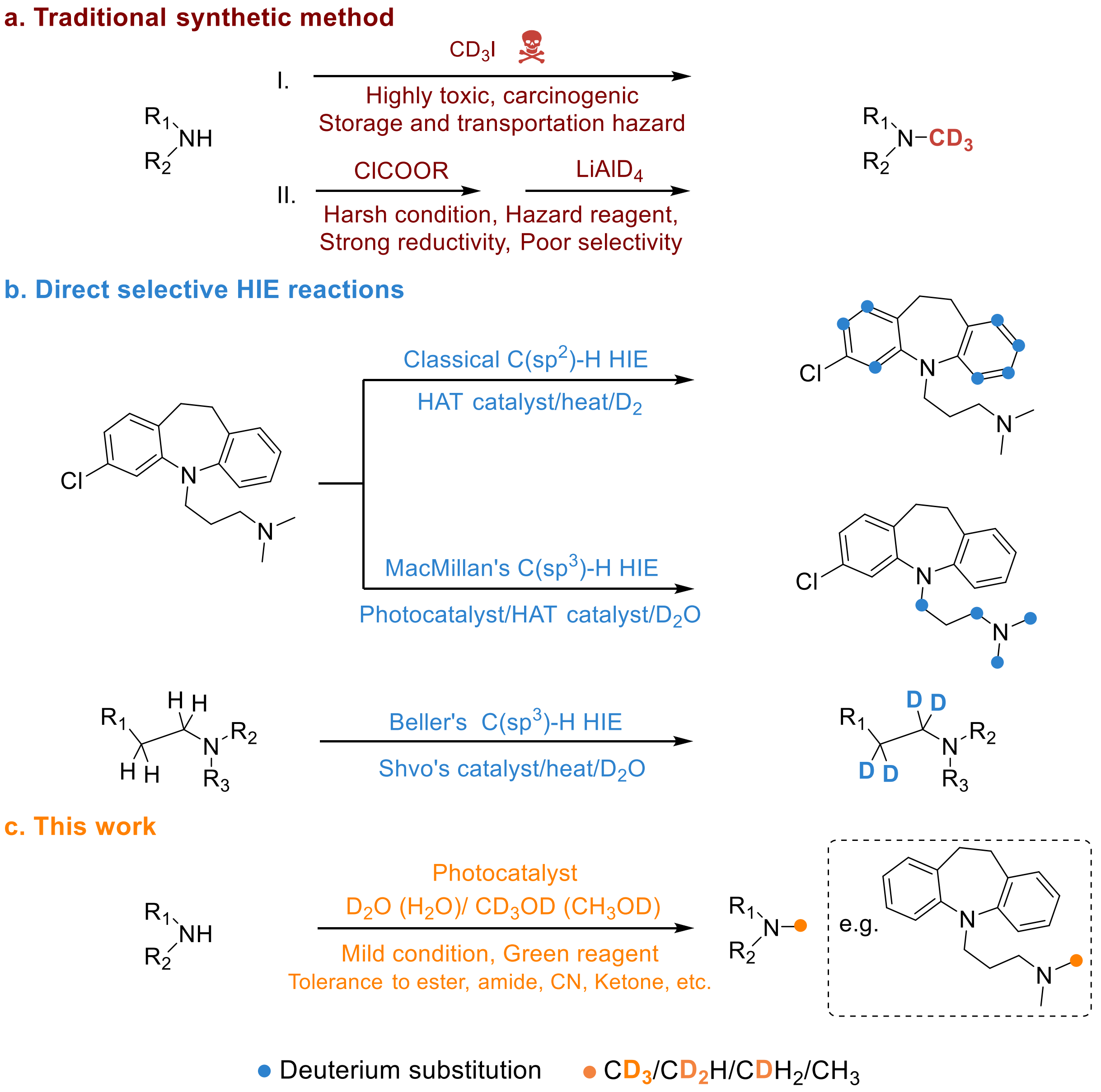

Supplementary Figure 2. Comparison of diverse strategies for synthesis of deuterated *N*-alkyl amines. a: traditional synthetic method, I: CD_3I as the deuterated methylation reagent; II: $LiAlD_4$ strategy for the incorporation of N-CD_3 group; b: direct selective HIE reactions; c: this work.

Supplementary Discussion

First, deuterated alkylation of amines with CD_3I is well-explored (Supplementary Figure 2, route a-I). However, these deuterated reagents are highly cancerogenic, and volatile, thus hinders their practical applications. Next to that, alkylation with CD_3I can lead to ammonium salts.

Reduction of *N*-CO_2R moieties with $LiAlD_4$ has good potential for the introduction of *N*-CD_3 group without formation of ammonium salts. Nevertheless, the drawbacks of this approach are obvious, such as introduction of extra functional group, use of hazard and strong reduced reagent ($LiAlH_4$/$LiAlD_4$), poor selectivity to e.g. amide, ester, cyano, ketone groups (Supplementary Figure 2, route a-II). Second, transition metal-catalyzed HIE reactions at aromatic C(sp^2)–H moieties are well established for deuterium and tritium labelling, in which directing groups are generally required.[1, 2] The direct HIE at aliphatic C(sp^3)–H moieties by transition metal-catalyst remains a challenge in the field.[3] Recently, Beller group[4] had demonstrated the transition metal HIE at both aliphatic α and β-amino C(sp^3)–H bonds. The key steps for this transformation include the generation of a reactive iminium cation or enamine intermediate followed by subsequent rehydrogenation. High temperature and complex noble metal catalyst is generally required. Third, MacMillan group[3] reported a powerful photo-redox mediated HIE reaction which could efficiently and selectively install deuterium or tritium at α-amino sp^3 C–H bonds of the *N*-alkyl amine based drug molecules. The key steps for this transformation include the generation of a reactive α-amino radical from *N*-alkyl amines catalyzed by a molecule photo catalyst followed by subsequent HAT catalysis with D_2O or T_2O. Although HIE methods are useful, they cann't be the substitutions to classic method with CD_3I (Supplementary Figure 2, route b). Herein, we have achieved a controllable isotope-labeling *N*-alkylation of amines with a combined alkylation reagents (isotopic water and alkanols), which could be a good substitution to traditional deuterated methylation of amines with CD_3I. More importantly, this work enables controllable installation of -CH_3, -CDH_2, -CD_2H, -CD_3, and -$^{13}CH_3$ into pharmaceutical amines by facilely tuning isotopic water and methanol under mild conditions (Supplementary Figure 2, route c). Also, the mechanism in this work is very different with the reported photocatalytic HIE.

In photocatalytic HIE, photoexcitation of the Ir_{III}-photocatalyst generates the long lived triplet excited state Ir_{III} complex, which oxidize amine at the α-position to give α-amino radical. Then the radical is trapped by the HAT catalysis to furnish α-deuterated or tritiated amine and the electrophilic thiol radical. A second single-electron transfer was occurred between Ir_{II} complex and electrophilic thiol radical to regenerate Ir_{III}-photocatalyst.[3] In this work, redox centers as electron-hole pairs are generated on semiconductor polymeric carbon nitride. Two aligned redox transformations named water-reduction and isotopic alkanol oxidation are accomplished on the same semiconductor surface. Amine substrates react with isotopic aldehyde from isotopic alkanol oxidation furnishes the iminium cation, which undergoes subsequent hydrogenation/deuteration by [H]/[D] to produce the corresponding products.

Supplementary Table 1. Optimization of the Reaction Conditions,[a] and screening of reaction conditions for the reaction of *p*-toluidine

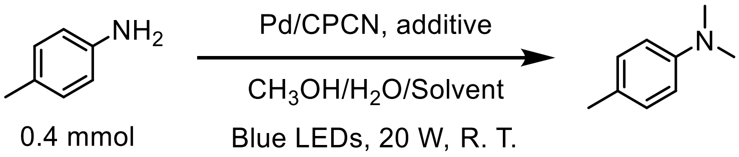

entry	solvent	additive	V_{CH3OH} (mL)	m_{cat} (mg)	Yield (%)[b]
1	EtOAC	$AlCl_3$	1.5	30	39
2	CH_3CN	$AlCl_3$	1.5	30	93
3	DMF	$AlCl_3$	1.5	30	trace
4	CH_3CN	$NaHSO_4$	1.5	30	75
5	CH_3CN	$AlCl_3$	1.5	20	89
6[c]	CH_3CN	$AlCl_3$	1.5	25	19
7	CH_3CN	$AlCl_3$	0.8	25	93
8	CH_3CN	$AlCl_3$	0.6	25	93
9	CH_3CN	$AlCl_3$	0.4	25	89
10[d]	CH_3CN	$AlCl_3$	-	25	89[e]
11[d]	CH_3CN	$AlCl_3$	-	15	80[e]
12[d, f]	CH_3CN	$AlCl_3$	-	25	trace

[a]Reactions conditions: *p*-toluidine (0.4 mmol), catalyst 3.0 wt% Pd/CPCN, $CH_3CN/H_2O/CH_3OH$ = 2 mL/1.5 mL/1.5 mL, 0.3 mmol additive, reaction time 12 h, 420 nm LED light 20 W at room temperature; [b]NMR yield; [c]Without H_2O; [d]Replacing H_2O/CH_3OH with D_2O/CD_3OD (1.5 mL/1.0 mL); [e]Isolated yield; [f]Without D_2O.

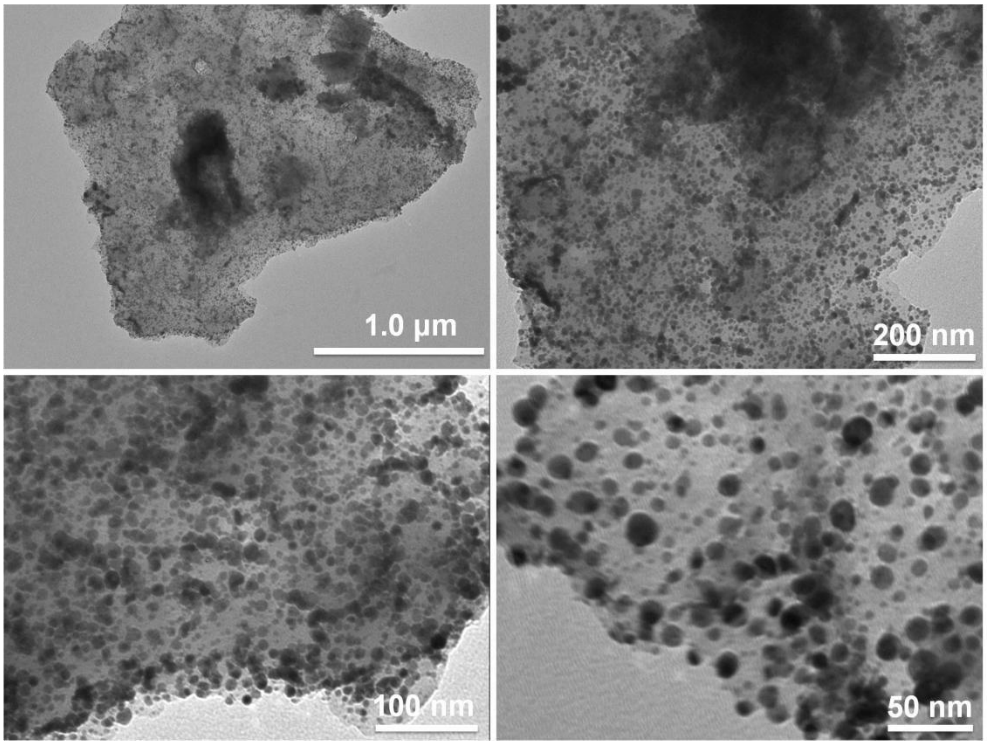

Supplementary Figure 3. TEM images of Pd/CPCN photocatalyst.

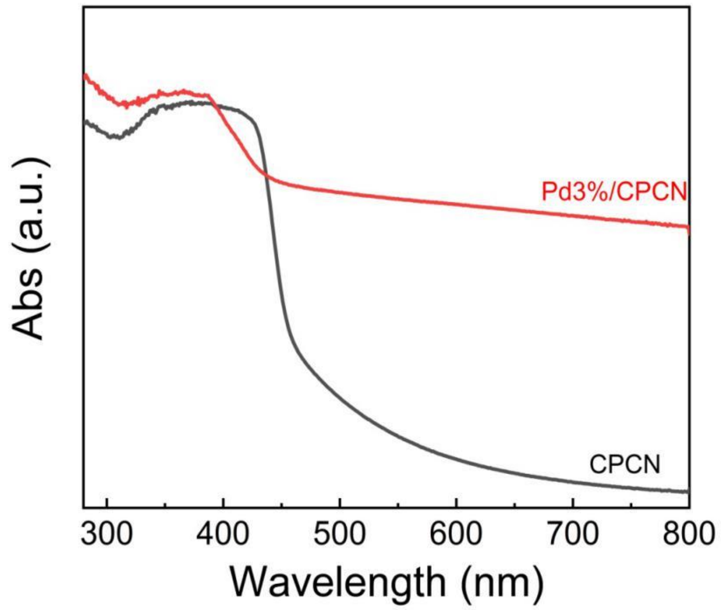

Supplementary Figure 4. UV-Vis spectra of CPCN and Pd/CPCN photocatalyst.

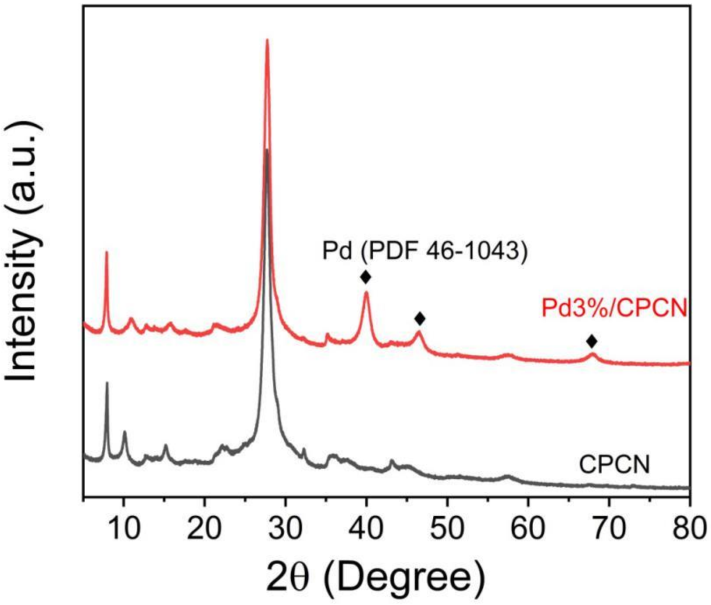

Supplementary Figure 5. XRD patterns of CPCN and Pd/CPCN photocatalyst.

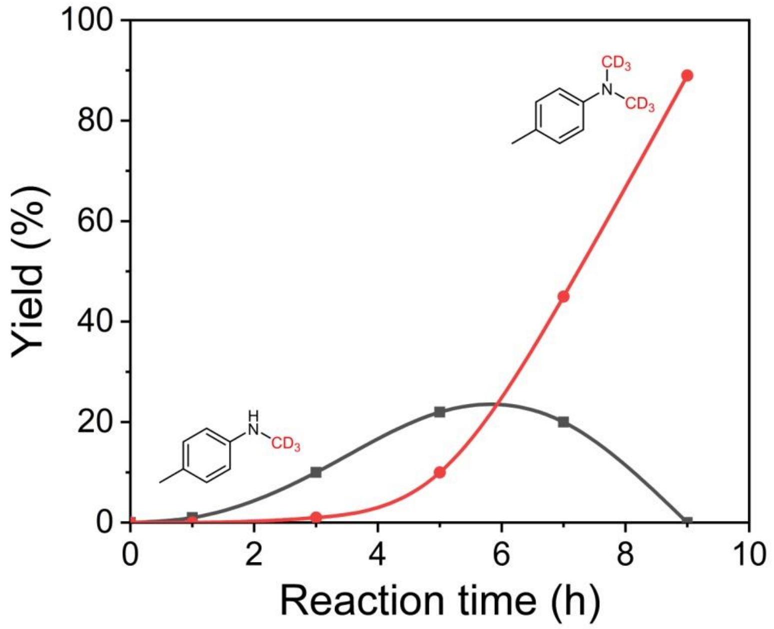

Supplementary Figure 6. Time-dependent yield of alkylation products.

Supplementary Methods

Gram scale synthesis of Loxapine-d_3 (4j)

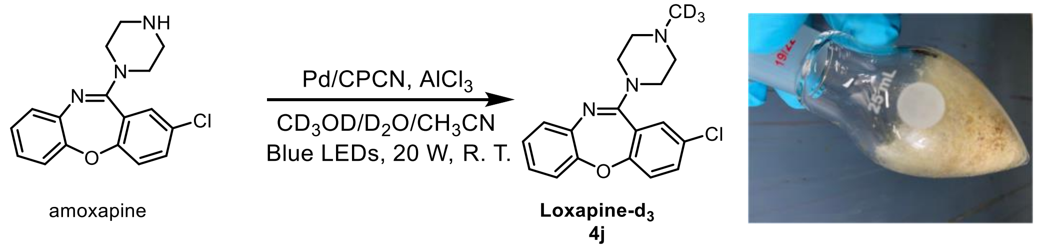

Supplementary Figure 7. Gram scale synthesis of Loxapine-d_3 (**4j**).

250 mg of Pd/CPCN, 5.0 mmol of amoxapine and $AlCl_3$ (3 mmol) were dispersed in a mixture solution with Acetonitrile/D_2O/CD_3OD=20 ml/15 ml/8 ml, and then sonicated for 10 min. The reaction mixture was then irradiated with a LED lamp (20W, λ= 420 nm) for 36 h under Argon at 25 ^oC by using a flow of cooling water during the reaction. After reaction, the mixture was centrifuged to remove photocatalyst. The supernatant was extracted by adding 50 mL of CH_2Cl_2. The reaction mixture was concentrated under reduced pressure and the residue was purified by column chromatography on silica gel to furnish the **Loxapine-d_3 (4j)** (1.4 g, 85%).

Gram scale synthesis of Dofetilide-d_3 (4n)

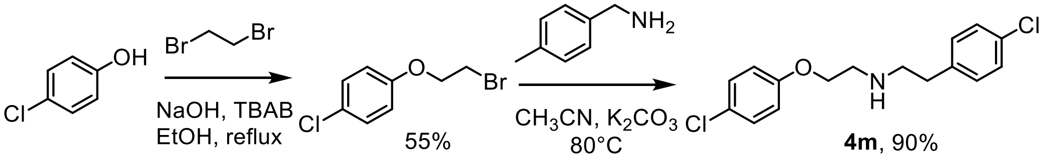

Supplementary Figure 8. Synthesis of **4m** in 50% yield in two steps following to the reported procedure.[5]

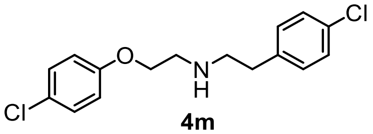

4m

N-(4-chlorophenethyl)-2-(4-chlorophenoxy)ethan-1-amine

^{1}H NMR (600 MHz, Chloroform-*d*) δ 7.28 (d, *J* = 8.3 Hz, 2H), 7.24 (d, *J* = 8.9 Hz, 2H), 7.17 (d, *J* = 8.3 Hz, 2H), 6.81 (d, *J* = 8.9 Hz, 2H), 4.04 (t, *J* = 5.2 Hz, 2H), 3.02 (t, *J* = 5.2 Hz, 2H), 2.94 (t, *J* = 7.2 Hz, 2H), 2.82 (t, *J* = 7.2 Hz, 2H); ^{13}C NMR (151 MHz, $CDCl_3$) δ 157.38, 138.34, 131.98, 130.06, 129.33, 128.59, 125.75, 115.77, 67.63, 50.88, 48.57, 35.77. HRMS (ESI) calcd for $C_{16}H_{18}NOCl_2$ $[M+H]^+$: 310.0760, found 310.0774

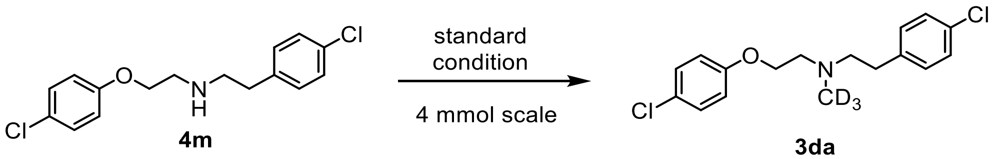

Supplementary Figure 9. Synthesis of **3da**.

250 mg of Pd/CPCN, 4.0 mmol of **4m** and $AlCl_3$ (3 mmol) were dispersed in a mixture solution with Acetonitrile/D_2O/CD_3OD=20 ml/15 ml/8 ml, and then sonicated for 10 min. The reaction mixture was then irradiated with a LED lamp (20W, λ= 420 nm) for 36 h under Argon at 25°C by using a flow of cooling water during the reaction. After reaction, the mixture was centrifuged to remove photocatalyst. The supernatant was extracted by adding 50 mL of CH_2Cl_2. The reaction mixture was concentrated under reduced pressure and used in the next step without purification (1.17 g, 90%).

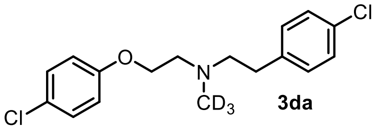

3da

N-(4-chlorophenethyl)-2-(4-chlorophenoxy)-N-(methyl-d$_3$)ethan-1-amine

^{1}H NMR (500 MHz, Chloroform-*d*) δ 7.26 – 7.21 (m, 4H), 7.16 – 7.10 (m, 2H), 6.83 – 6.79 (m, 2H), 4.12 (t, *J* = 5.5 Hz, 2H), 3.00 (t, *J* = 5.5 Hz, 2H), 2.91 – 2.78 (m, 4H), 2.47 (s, 0.1H).; ^{13}C NMR (126 MHz, $CDCl_3$) δ 157.02, 137.76, 132.12, 130.07, 129.39, 128.63, 125.96, 115.84,

65.71, 59.15, 55.65, 41.60 (d, J = 20.8 Hz), 32.37. HRMS (ESI) calcd for $C_{17}D_3H_{17}NOCl_2$ $[M+H]^+$: 327.1105, found 327.1114.

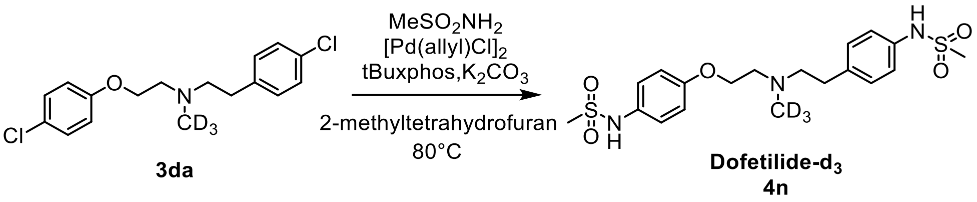

Supplementary Figure 10. Synthesis of **4n**.

In the glove box,[6] a 100 mL round-bottom flask was charged with **3da** (1.17 g, 1.54 mmol), methanesulfonamide (520 mg, 5.46 mmol), K_2CO_3 (1.21 g, 8.74 mmol), *t*BuXPhos (92.1 mg, 0.218 mmol), $[Pd(allyl)Cl]_2$ (19.8 mg, 0.055 mmol) and 2-methyltetrahydrofuran (40 mL) and a stir bar. The reaction mixture was heated to 80 ℃ with stirring overnight. The reaction was cooled to room temperature and diluted with EtOAc (50 mL) and saturated NH_4Cl solution (50 mL). The mixture was stirred at room temperature for 1 hour. The layers were separated and the organics were dried over $MgSO_4$, filtered, and concentrated. The crude product was purified by flash chromatography system to afford **Dofetilide-*d*$_3$** (**4n**).

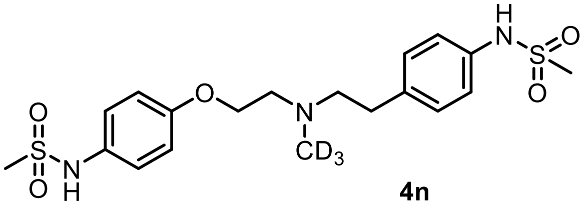

4n

N-(4-(2-((methyl-d$_3$)(2-(4

(methylsulfonamido)phenoxy)ethyl)amino)ethyl)phenyl)methanesulfonamide

Purified by FC (eluent: DCM/methanol = 20: 1) to afford the product **4n** (1.1 g, yield: 70%) as a white solid, ^{1}H NMR (500 MHz, Acetone-d_6) δ 7.28 (d, J = 8.9 Hz, 1H), 7.25 (s, 2H), 6.94 (d, J = 8.9 Hz, 1H), 4.08 (t, J = 5.9 Hz, 1H), 2.94 (s, 3H), 2.90 (s, 3H), 2.84 (t, J = 5.9 Hz, 1H), 2.78 (ddd, J = 9.3, 6.7, 2.2 Hz, 1H), 2.74 – 2.67 (m, 1H); ^{13}C NMR (126 MHz, Acetone) δ 156.76,

137.21, 136.25, 130.96, 129.63, 123.86, 120.76, 115.08, 66.65, 59.54, 55.88, 45.44, 42.58, 38.30, 37.95, 32.84. HRMS (ESI) calcd for $C_{19}D_3H_{25}N_3O_5S_2$ $[M+H]^+$: 445.1653, found 445.1670.

Supplementary Characterization of Products

^{1}H-NMR, ^{13}C-NMR and high resolution mass spectrometry of Products

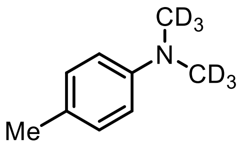

1a or **3aa**

4-methyl-N,N-bis(methyl-d$_3$)aniline

Purified by FC (eluent: EtOAc/n-hexane = 1: 100) to afford the product **1a** or **3aa** (50.3 mg, yield: 89%) as a colorless oil; ^{1}H NMR (500 MHz, Chloroform-*d*) δ 7.05 (d, J = 8.5 Hz, 2H), 6.68 (d, J = 8.5 Hz, 2H), 2.85 (p, J = 1.8 Hz, 0.21H), 2.25 (s, 3H).; ^{13}C NMR (126 MHz, $CDCl_3$) δ 148.90, 129.59, 126.06, 113.16, 40.24 (d, J = 20.2 Hz), 20.26. HRMS (ESI) calcd for $C_9 D_6H_8N$ $[M+H]^+$: 142.1497, found 142.1496.

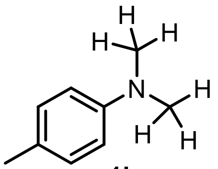

1b

N,N,4-trimethylaniline

Synthesized following the general procedure except using CH_3OH and H_2O. Purified by FC (eluent: EtOAc/n-hexane = 1: 100) to afford the product **1b** (50.8 mg, yield: 94%) as a colorless oil, ^{1}H NMR (500 MHz, Chloroform-*d*) δ 7.04 (d, J = 7.7 Hz, 2H), 6.69 (d, J = 1.4 Hz, 2H), 2.88 (s, 6H), 2.25 (s, 3H); ^{13}C NMR (126 MHz, $CDCl_3$) δ 148.91, 129.65, 126.13, 113.27, 41.12, 20.32. HRMS (ESI) calcd for $C_9H_{14}N$ $[M+H]^+$: 136.1121, found 136.1117.

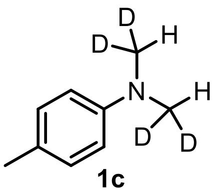

1c

4-methyl-N,N-bis(methyl-d_2)aniline

Synthesized following the general procedure except using CD_3OD and H_2O. Purified by FC (eluent: EtOAc/n-hexane = 1: 100) to afford the product **1c** (50.8 mg, yield: 91%) as a colorless oil, ^1H NMR (600 MHz, Chloroform-*d*) δ 7.08 (d, *J* = 8.4 Hz, 2H), 6.71 (d, *J* = 8.6 Hz, 2H), 2.88 (p, *J* = 1.8 Hz, 1.84H), 2.86 (s, 0.16H), 2.28 (s, 3H).; ^{13}C NMR (126 MHz, $CDCl_3$) δ 148.89, 129.60, 126.09, 113.20, 40.48 (t, *J* = 20.8 Hz), 20.26. HRMS (ESI) calcd for $C_9D_4H_{10}N$ $[M+H]^+$: 140.1372, found 140.1366.

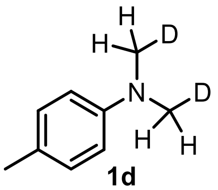

1d

4-methyl-N,N-bis(methyl-d)aniline

Synthesized following the general procedure except using CH_3OD and D_2O. Purified by FC (eluent: EtOAc/n-hexane = 1: 100) to afford the product **1d** (49.9 mg, yield: 91%) as a colorless oil, ^1H NMR (500 MHz, Chloroform-*d*) δ 7.08 – 7.02 (m, 2H), 6.72 – 6.65 (m, 2H), 2.89 (s, 0.52H), 2.88 – 2.86 (m, 1.48H), 2.25 (s, 3H); ^{13}C NMR (126 MHz, $CDCl_3$) δ 148.87, 129.59, 126.12, 113.23, 40.79 (t, *J* = 20.8 Hz), 20.26. HRMS (ESI) calcd for $C_9D_2H_{12}N$ $[M+H]^+$: 138.1246, found 138.1244.

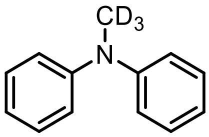

2a or **3ca**

N-(methyl-d_3)-N-phenylaniline

Purified by FC (eluent: EtOAc/n-hexane = 1: 200) to afford the product **2a** or **3ca** (68.7 mg, yield: 92%) as a colorless liquid; ^{1}H NMR (500 MHz, Chloroform-*d*) δ 7.28 – 7.22 (m, 4H), 7.03 – 6.99 (m, 4H), 6.94 (td, *J* = 7.4, 1.2 Hz, 2H), 3.26 (p, *J* = 1.8 Hz, 0.1H); ^{13}C NMR (126 MHz, $CDCl_3$) δ 149.08, 129.25, 121.29, 120.48, 39.42 (t, *J* = 20.8 Hz). HRMS (ESI) calcd for $C_{13}D_3H_{11}N$ $[M+H]^+$: 187.1309, found 187.1304.

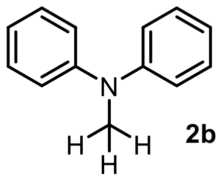

2b

N-methyl-N-phenylaniline

Synthesized following the general procedure except using CH_3OH and H_2O. Purified by FC (eluent: EtOAc/n-hexane = 1: 200) to afford the product **2b** (69.1 mg, yield: 94%) as a colorless liquid; ^{1}H NMR (500 MHz, Chloroform-*d*) δ 7.25 (dddd, *J* = 9.4, 7.3, 4.7, 2.6 Hz, 4H), 7.01 (ddd, *J* = 8.4, 5.3, 2.4 Hz, 4H), 6.93 (dq, *J* = 7.6, 3.9, 2.6 Hz, 2H), 3.29 (s, 3H); ^{13}C NMR (126 MHz, $CDCl_3$) δ 149.12, 129.27, 121.34, 120.53, 40.32. HRMS (ESI) calcd for $C_{13}H_{14}N$ $[M+H]^+$: 184.1121, found 184.1119.

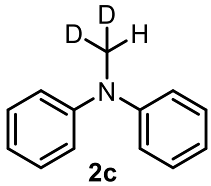

2c

N-(methyl-d_2)-N-phenylaniline

Synthesized following the general procedure except using CD_3OD and H_2O. Purified by FC (eluent: EtOAc/n-hexane = 1: 200) to afford the product **2c** (64.7 mg, yield: 87%) as a colorless liquid; ^{1}H NMR (500 MHz, Chloroform-*d*) δ 7.28 – 7.23 (m, 4H), 7.04 – 6.98 (m, 4H), 6.94 (tt, *J*

= 7.3, 1.2 Hz, 2H), 3.27 (p, *J* = 1.8 Hz, 1H); ^{13}C NMR (126 MHz, $CDCl_3$) δ 149.07, 129.23, 121.29, 120.47, 40.09-39.42 (t, *J* = 20.8 Hz). HRMS (ESI) calcd for $C_{13}D_2H_{12}N$ $[M+H]^+$: 186.1246, found 186.1248.

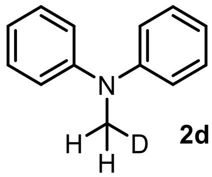

2d

N-(methyl-d)-N-phenylaniline

Synthesized following the general procedure except using CH_3OD and D_2O.Purified by FC (eluent: EtOAc/n-hexane = 1: 200) to afford the product **2d** (54.7 mg, yield: 74%) as a colorless liquid; ^1H NMR (500 MHz, Chloroform-*d*) δ 7.29 – 7.24 (m, 4H), 7.04 – 6.99 (m, 4H), 6.98 – 6.92 (m, 2H), 3.31 (s, 0.20H), 3.30 – 3.28 (m, 1.80H); ^{13}C NMR (126 MHz, $CDCl_3$) δ 149.05, 129.20, 121.26, 120.45, 40.00 (t, *J* = 20.8 Hz). HRMS (ESI) calcd for $C_{13}DH_{13}N$ $[M+H]^+$: 185.1184, found 185.1179.

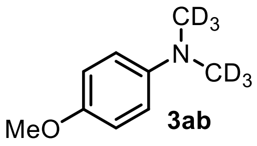

3ab

4-methoxy-N,N-bis(methyl-d$_3$)aniline

Purified by FC (eluent: EtOAc/n-hexane = 1: 50) to afford the product **3ab** (46.3 mg, yield: 74%) as a white solid, ^1H NMR (500 MHz, Chloroform-*d*) δ 6.84 (dd, *J* = 9.1, 2.4 Hz, 2H), 6.74 (dd, *J* = 9.1, 2.4 Hz, 2H), 3.76 (d, *J* = 2.3 Hz, 3H), 2.81 (q, *J* = 1.9 Hz, 0.17H).; ^{13}C NMR (126 MHz, $CDCl_3$) δ 151.95, 145.84, 114.84, 114.66, 55.79, 40.91 (t, *J* = 20.8 Hz). HRMS (ESI) calcd for $C_9D_6H_8NO$ $[M+H]^+$: 158.1447, found 158.1445.

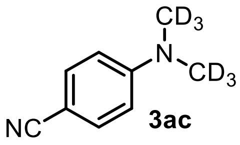

3ac

4-(bis(methyl-d_3)amino)benzonitrile

Purified by FC (eluent: EtOAc/n-hexane = 1: 100) to afford the product **3ac** (41.8 mg, yield: 69%) as a colorless oil, ^{1}H NMR (500 MHz, Chloroform-*d*) δ 7.46 (d, *J* = 9.0 Hz, 2H), 6.63 (d, *J* = 9.0 Hz, 2H), 3.00 (s, 0.16H); ^{13}C NMR (126 MHz, $CDCl_3$) δ 152.53, 133.40, 120.75, 111.34, 97.29, 39.06 (t, *J* = 20.8 Hz). HRMS (ESI) calcd for $C_9H_5N_2D_6$ $[M+H]^+$: 153.1293, found 153.1288.

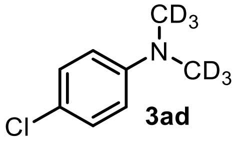

3ad

4-chloro-N,N-bis(methyl-d_3)aniline

Purified by FC (eluent: EtOAc/n-hexane = 1: 100) to afford the product **3ad** (54.7 mg, yield: 85%) as a colorless oil, ^{1}H NMR (500 MHz, Chloroform-*d*) δ 7.16 (d, *J* = 9.1 Hz, 1H), 6.61 (d, *J* = 9.1 Hz, 1H), 2.87-2.86 (p, *J* = 1.9 Hz, 0.17H); ^{13}C NMR (126 MHz, $CDCl_3$) δ 149.24, 128.82, 121.35, 113.59, 39.94 (hept, *J* = 20.8 Hz). HRMS (ESI) calcd for $C_8D_6H_5NCl$ $[M+H]^+$: 162.0951, found 162.0947.

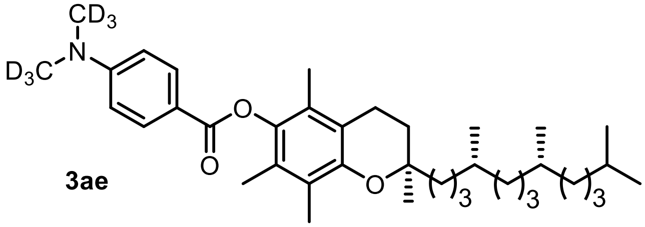

3ae

2,5,7,8-tetramethyl-2-((2R,4R)-2,4,6-trimethylheptyl)chroman-6-yl 4-(bis(methyl-d_3)amino)benzoate

0.2 mmol scale. Purified by FC (eluent: EtOAc/n-hexane = 1: 15) to afford the product **3ae** (91.4 mg, yield: 78%) as a colorless oil, ^{1}H NMR (500 MHz, Chloroform-*d*) δ 8.11 (d, *J* = 9.0 Hz, 2H), 6.71 (d, *J* = 9.0 Hz, 2H), 3.03 (p, *J* = 1.8 Hz, 0.1H), 2.61 (t, *J* = 6.8 Hz, 2H), 2.11 (s, 3H), 2.05 (s,

3H), 2.01 (s, 3H), 1.86-1.72 (m, 2H), 1.55-1.50 (m, 2H), 1.43-1.36 (m, 4H), 1.34 – 1.19 (m, 12H), 1.19 – 1.02 (m, 6H), 0.87-0.87 (m, 12H); ^{13}C NMR (126 MHz, $CDCl_3$) δ 165.51, 153.69, 149.19, 140.85, 131.96, 127.25, 125.43, 122.91, 117.33, 116.19, 110.77, 74.98, 39.55-38.89 (m), 39.40, 37.52, 37.49, 37.42, 37.32, 32.82, 28.01, 24.85, 24.48, 22.75, 22.66, 21.08, 20.66, 19.78, 19.72, 19.63, 13.08, 12.23, 11.86. HRMS (ESI) calcd for $C_{38}D_6H_{54}NO_3$ $[M+H]^+$: 584.4944, found 584.4959.

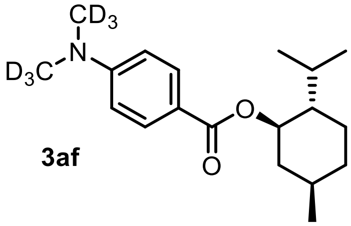

3af

(1R,2S,5R)-2-isopropyl-5-methylcyclohexyl 4-(bis(methyl-d$_3$)amino)benzoate

0.3 mmol scale. Purified by FC (eluent: EtOAc/n-hexane = 1: 20) to afford the product **3af** (65.9 mg, yield: 71%) as a white soild, ^{1}H NMR (500 MHz, Chloroform-*d*) δ 7.91 (d, *J* = 9.0 Hz, 2H), 6.64 (d, *J* = 8.9 Hz, 2H), 4.87 (td, *J* = 10.9, 4.4 Hz, 1H), 2.99 (t, *J* = 1.9 Hz, 0.09H), 2.12 (dtd, *J* = 12.0, 3.7, 1.7 Hz, 1H), 1.97 (heptd, *J* = 7.0, 2.8 Hz, 1H), 1.75 – 1.71 (m, 1H), 1.70 (t, *J* = 3.0 Hz, 1H), 1.60-1.49 (m, 2H), 1.18 – 1.09 (m, 1H), 1.06 (dd, *J* = 12.5, 11.1 Hz, 1H), 0.91 (t, *J* = 6.7 Hz, 6H), 0.79 (d, *J* = 6.9 Hz, 3H); ^{13}C NMR (126 MHz, $CDCl_3$) δ 166.51, 153.28, 131.22, 117.72, 110.64, 73.78, 47.41, 41.21, 39.21 (t, *J* = 21.4 Hz), 34.45, 31.46, 26.54, 23.78, 22.11, 20.80, 16.66. HRMS (ESI) calcd for $C_{19}D_6H_{24}NO_2$ $[M+H]^+$: 310.2648, found 310.2661.

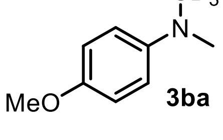

3ba

4-methoxy-N-methyl-N-(methyl-d$_3$)aniline

Purified by FC (eluent: EtOAc/n-hexane = 1: 100) to afford the product **3ba** (56.7 mg, yield: 92%) as a dark oil, ^{1}H NMR (500 MHz, Chloroform-*d*) δ 6.84 (d, *J* = 9.1 Hz, 2H), 6.74 (d, *J* = 9.1 Hz, 2H), 3.75 (s, 3H), 2.85 (s, 3H), 2.85-2.81 (p, *J* = 1.7 Hz, 0.1H); ^{13}C NMR (126 MHz, $CDCl_3$) δ 152.00, 145.82, 114.91, 114.66, 55.78, 41.77, 41.02 (t, *J* = 20.8 Hz). HRMS (ESI) calcd for $C_9H_{11}NOD_3$ $[M+H]^+$: 155.1258, found 155.1252.

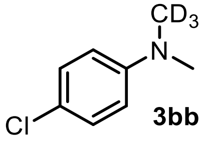

3bb

4-chloro-N-methyl-N-(methyl-d$_3$)aniline

Purified by FC (eluent: EtOAc/n-hexane = 1: 100) to afford the product **3bb** (53.4 mg, yield: 84%) as a colorless oil, ^{1}H NMR (500 MHz, Chloroform-*d*) δ 7.17 (d, *J* = 9.1 Hz, 2H), 6.63 (d, *J* = 9.1 Hz, 2H), 2.92 (s, 3H), 2.89-2.88 (m, 0.09H); ^{13}C NMR (126 MHz, $CDCl_3$) δ 149.20, 128.80, 121.40, 113.61, 40.59. HRMS (ESI) calcd for $C_8D_3H_8NCl$ $[M+H]^+$: 159.0763, found 159.0761.

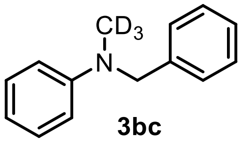

3bc

N-benzyl-N-(methyl-d$_3$)aniline

Purified by FC (eluent: EtOAc/n-hexane = 1: 100) to afford the product **3bc** (73.0 mg, yield: 91%) as a colorless oil, ^{1}H NMR (500 MHz, Chloroform-*d*) δ 7.30 (dd, *J* = 8.2, 6.9 Hz, 2H), 7.24 – 7.18 (m, 5H), 6.74 (d, *J* = 8.3 Hz, 2H), 6.70 (t, *J* = 7.3 Hz, 1H), 4.51 (s, 2H), 2.97-2.96 (p, *J* = 1.8 Hz, 0.08H); ^{13}C NMR (126 MHz, $CDCl_3$) δ 149.83, 139.11, 129.23, 129.12, 128.60, 126.90, 126.78, 116.55, 112.38, 56.59, 37.91 (hept, *J* = 20.8 Hz). HRMS (ESI) calcd for $C_{14}H_{13}ND_3$ $[M+H]^+$: 201.1466, found 201.1464.

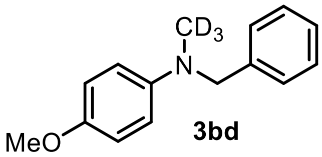

3bd

N-benzyl-4-methoxy-N-(methyl-d$_3$)aniline

Purified by FC (eluent: EtOAc/n-hexane = 1: 100) to afford the product **3bd** (86.9 mg, yield: 94%) as a colorless oil, ^{1}H NMR (500 MHz, Chloroform-*d*) δ 7.33 – 7.27 (m, 2H), 7.26 – 7.19 (m, 3H), 6.81 (dd, *J* = 9.1, 1.4 Hz, 2H), 6.73 (dd, *J* = 9.1, 1.4 Hz, 2H), 4.41 (s, 2H), 3.74 (s, 3H), 2.91 – 2.83 (m, 0.08H); ^{13}C NMR (126 MHz, $CDCl_3$) δ 151.76, 144.84, 139.27, 128.49, 127.12, 126.86, 114.77, 114.51, 57.94, 55.80, 38.26 (t, *J* = 20.8 Hz). HRMS (ESI) calcd for $C_{15}D_3H_{15}NO$ [M+H]$^+$: 231.1571, found 231.1575.

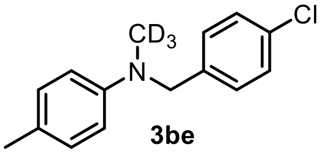

3be

N-(4-chlorobenzyl)-4-methyl-N-(methyl-d$_3$)aniline

Purified by FC (eluent: EtOAc/n-hexane = 1: 100) to afford the product **3be** (90.6 mg, yield: 91%) as a colorless oil, ^{1}H NMR (500 MHz, Chloroform-*d*) δ 7.25 (d, *J* = 8.4 Hz, 2H), 7.14 (d, *J* = 8.2 Hz, 2H), 7.02 (d, *J* = 8.2 Hz, 2H), 6.64 (d, *J* = 8.6 Hz, 2H), 4.41 (s, 2H), 2.90 (p, *J* = 1.8 Hz, 0.05H), 2.24 (s, 3H); ^{13}C NMR (126 MHz, $CDCl_3$) δ 147.64, 137.83, 132.52, 129.81, 128.69, 128.29, 126.17, 112.88, 56.50, 37.94 (t, *J* = 20.8 Hz), 20.31. HRMS (ESI) calcd for $C_{15}D_3H_{14}NCl$ [M+H]$^+$: 249.1232, found 249.1241.

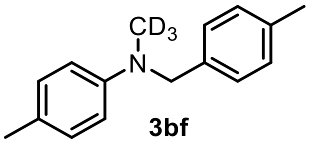

3bf

4-methyl-N-(methyl-d$_3$)-N-(4-methylbenzyl)aniline

Purified by FC (eluent: EtOAc/n-hexane = 1: 100) to afford the product **3bf** (77.5 mg, yield: 85%) as a colorless oil, ^{1}H NMR (500 MHz, Chloroform-*d*) δ 7.14 – 7.08 (m, 5H), 7.02 (d, *J* = 8.3 Hz, 2H), 6.67 (d, *J* = 8.7 Hz, 2H), 4.43 (s, 2H), 2.90 (p, *J* = 1.8 Hz, 0.07H), 2.32 (s, 3H), 2.24 (s, 3H); ^{13}C NMR (126 MHz, $CDCl_3$) δ 147.92, 136.37, 136.15, 129.70, 129.20, 126.88, 125.69, 112.74, 56.67, 37.75 (t, *J* = 20.8 Hz), 21.10, 20.27. HRMS (ESI) calcd for $C_{16}D_3H_{17}N$ $[M+H]^+$: 229.1779, found 229.1781.

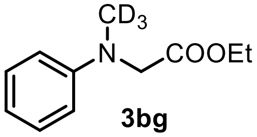

3bg

ethyl N-(methyl-d$_3$)-N-phenylglycinate

Purified by FC (eluent: EtOAc/n-hexane = 1: 100) to afford the product **3bg** (66.0 mg, yield: 84%) as a dark oil, ^{1}H NMR (500 MHz, Chloroform-*d*) δ 7.22 (dd, *J* = 8.8, 7.2 Hz, 2H), 6.74 (t, *J* = 7.3 Hz, 1H), 6.70 – 6.64 (m, 2H), 4.16 (q, *J* = 7.1 Hz, 2H), 4.04 (s, 2H), 3.02 (p, *J* = 1.8 Hz, 0.07H), 1.23 (t, *J* = 7.1 Hz, 2H); ^{13}C NMR (126 MHz, $CDCl_3$) δ 171.07, 148.95, 129.20, 117.30, 112.31, 60.86, 54.47, 38.72 (p, *J* = 20.8 Hz), 14.26. HRMS (ESI) calcd for $C_{11}D_3H_{13}NO_2$ $[M+H]^+$: 197.1364, found 197.1365.

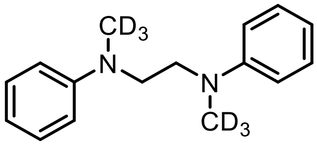

3bh

N1,N2-bis(methyl-d$_3$)-N1,N2-diphenylethane-1,2-diamine

Purified by FC (eluent: EtOAc/n-hexane = 1: 40) to afford the product **3bh** (78.0 mg, yield: 79%) as a colorless oil, ^{1}H NMR (500 MHz, Chloroform-*d*) δ 7.26 – 7.21 (m, 4H), 6.73 – 6.67 (m, 6H), 3.53 (s, 4H), 2.90 (p, *J* = 1.8 Hz, 0.21H); ^{13}C NMR (126 MHz, $CDCl_3$) δ 148.92, 129.33, 116.24,

111.82, 49.69, 37.91 (t, *J* = 21.4 Hz). HRMS (ESI) calcd for $C_{16}D_6H_{15}N_2$ $[M+H]^+$: 247.2076, found 247.2086.

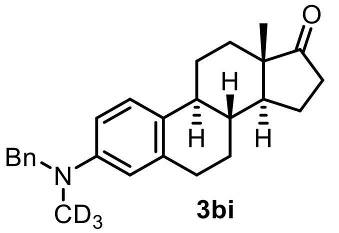

3bi

(8R,9S,13S,14S)-3-(benzyl(methyl-d$_3$)amino)-13-methyl-6,7,8,9,11,12,13,14,15,16-decahydro-17H-cyclopenta[a]phenanthren-17-one

0.3 mmol scale. Purified by FC (eluent: EtOAc/n-hexane = 1: 30) to afford the product **3bi** (84.6 mg, yield: 75%) as a white solid, ^{1}H NMR (500 MHz, Chloroform-*d*) δ 7.31 (dd, *J* = 8.1, 6.7 Hz, 2H), 7.26 – 7.22 (m, 4H), 7.14 (d, *J* = 8.6 Hz, 1H), 6.60 (dd, *J* = 8.7, 2.6 Hz, 1H), 6.50 (d, *J* = 2.7 Hz, 1H), 4.48 (s, 2H), 2.94 – 2.92 (m, 0.14H), 2.92 – 2.78 (m, 2H), 2.49 (dd, *J* = 19.0, 8.6 Hz, 1H), 2.40 – 2.34 (m, 1H), 2.23 (td, *J* = 10.6, 4.1 Hz, 1H), 2.13 (dt, *J* = 18.9, 8.9 Hz, 1H), 2.08 – 1.89 (m, 3H), 1.65 – 1.56 (m, 3H), 1.56 – 1.45 (m, 3H), 1.45 – 1.41 (m, 1H), 0.90 (s, 3H); ^{13}C NMR (151 MHz, $CDCl_3$) δ 221.23, 148.07, 139.33, 137.14, 128.53, 128.35, 128.06, 126.81, 126.12, 112.54, 110.55, 56.67, 50.43, 48.12, 43.95, 38.62, 35.93, 31.63, 29.98, 26.78, 25.92, 21.61, 13.91. HRMS (ESI) calcd for $C_{26}D_3H_{29}NO$ $[M+H]^+$: 377.2667, found 377.2671.

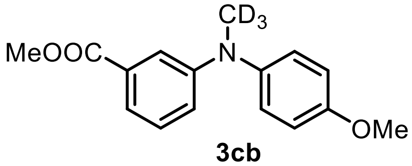

3cb

methyl 3-((4-methoxyphenyl)(methyl-d$_3$)amino)benzoate

Purified by FC (eluent: EtOAc/n-hexane = 1: 100) to afford the product **3cb** (89.9 mg, yield: 82%) as a colorless oil, ^{1}H NMR (500 MHz, Chloroform-*d*) δ 7.44 (dd, *J* = 2.7, 1.5 Hz, 1H), 7.42 (dt, *J* = 7.8, 1.3 Hz, 1H), 7.20 (t, *J* = 7.9 Hz, 1H), 7.12 – 7.06 (m, 2H), 6.93 – 6.87 (m, 3H), 3.86

(s, 3H), 3.81 (s, 3H), 3.24 (p, J = 1.8 Hz, 0.11H); ^{13}C NMR (126 MHz, $CDCl_3$) δ 167.58, 156.82, 149.78, 141.58, 130.84, 128.80, 126.87, 119.57, 119.05, 115.35, 115.00, 55.50, 52.04, 39.80 (p, J = 20.8 Hz). HRMS (ESI) calcd for $C_{16}D_3H_{15}NO_3$ $[M+H]^+$: 275.1470, found 275.1483.

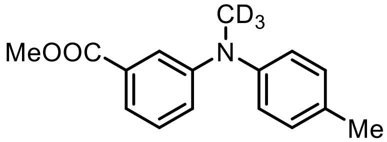

3cc

methyl 3-((methyl-d$_3$)(p-tolyl)amino)benzoate

Purified by FC (eluent: EtOAc/n-hexane = 1: 100) to afford the product **3cc** (83.5 mg, yield: 81%) as a colorless oil, ^{1}H NMR (500 MHz, Chloroform-*d*) δ 7.56 – 7.53 (m, 1H), 7.48 (dt, J = 7.6, 1.3 Hz, 1H), 7.23 (t, J = 7.9 Hz, 1H), 7.14 (d, J = 8.0 Hz, 2H), 7.02 (d, J = 8.2 Hz, 3H), 3.88 (s, 3H), 3.28 (p, J = 1.8 Hz, 0.08H), 2.33 (s, 3H); ^{13}C NMR (126 MHz, $CDCl_3$) δ 167.45, 149.41, 146.02, 133.31, 130.97, 130.19, 128.85, 123.78, 121.43, 120.12, 117.28, 52.06, 39.64 (t, J = 20.8 Hz), 20.85. HRMS (ESI) calcd for C_{16} $D_3H_{15}NO_2$ $[M+H]^+$: 259.1520, found 259.1533.

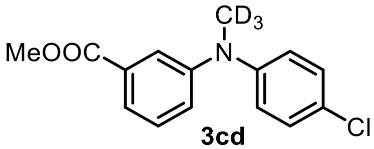

3cd

methyl 3-((4-chlorophenyl)(methyl-d$_3$)amino)benzoate

Purified by FC (eluent: EtOAc/n-hexane = 1: 100) to afford the product **3cd** (96.7 mg, yield: 87%) as a colorless oil, ^{1}H NMR (500 MHz, Chloroform-*d*) δ 7.65 (dd, J = 2.5, 1.6 Hz, 1H), 7.61 (dt, J = 7.8, 1.3 Hz, 1H), 7.30 (t, J = 7.9 Hz, 1H), 7.26 – 7.21 (m, 2H), 7.15 (ddd, J = 8.1, 2.5, 1.1 Hz, 1H), 6.99 – 6.93 (m, 2H), 3.89 (s, 3H), 3.28 (p, J = 1.8 Hz, 0.08H); ^{13}C NMR (126 MHz, $CDCl_3$) δ 167.07, 148.77, 147.16, 131.34, 129.41, 129.23, 127.16, 124.17, 122.47, 122.26, 120.32, 52.16, 39.68 (q, J = 20.8 Hz). HRMS (ESI) calcd for $C_{15}D_3H_{12}NO_2Cl$ $[M+H]^+$: 279.0979, found 279.0974.

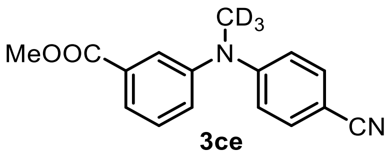

3ce

methyl 3-((4-cyanophenyl)(methyl-d$_3$)amino)benzoate

Purified by FC (eluent: EtOAc/n-hexane = 1: 100) to afford the product **3ce** (85.3 mg, yield: 79%) as a colorless oil, ^{1}H NMR (500 MHz, Chloroform-*d*) δ 7.91 (dt, *J* = 7.6, 1.4 Hz, 1H), 7.88 (t, *J* = 2.0 Hz, 1H), 7.49 (t, *J* = 7.9 Hz, 1H), 7.47 – 7.43 (m, 2H), 7.40 (ddd, *J* = 7.9, 2.3, 1.1 Hz, 1H), 6.80 – 6.74 (m, 2H), 3.92 (s, 3H), 3.35 (p, *J* = 1.9 Hz, 0.08H); ^{13}C NMR (126 MHz, $CDCl_3$) δ 166.38, 151.61, 147.02, 133.38, 132.20, 130.50, 130.07, 127.00, 126.86, 120.05, 114.55, 100.38, 52.36, 39.38 (t, *J* = 21.4 Hz). HRMS (ESI) calcd for $C_{16}D_3H_{12}N_2O_2$ $[M+H]^+$: 270.1316, found 270.1320.

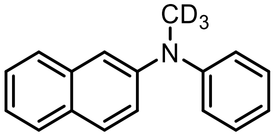

3cf

N-(methyl-d$_3$)-N-phenylnaphthalen-2-amine

Purified by FC (eluent: EtOAc/n-hexane = 1: 150) to afford the product **3cf** (69.3 mg, yield: 74%) as a brown oil, ^{1}H NMR (500 MHz, Chloroform-*d*) δ 7.73 (dd, *J* = 8.0, 1.1 Hz, 1H), 7.68 (t, *J* = 8.1 Hz, 2H), 7.41 (ddd, *J* = 8.2, 6.8, 1.3 Hz, 1H), 7.33 – 7.31 (m, 2H), 7.31 – 7.27 (m, 2H), 7.21 (dd, *J* = 8.9, 2.3 Hz, 1H), 7.13 – 7.07 (m, 2H), 7.01 (tt, *J* = 7.3, 1.1 Hz, 1H), 3.39 (p, *J* = 1.8 Hz, 0.07H); ^{13}C NMR (126 MHz, $CDCl_3$) δ 149.05, 146.58, 134.68, 129.27, 129.09, 128.55, 127.52, 126.70, 126.24, 123.69, 121.90, 121.78, 121.34, 114.54. HRMS (ESI) calcd for $C_{17}D_3H_{13}N$ $[M+H]^+$: 237.1466, found 237.1465.

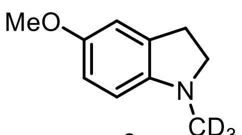

3cg

5-methoxy-1-(methyl-d$_3$)indoline

Purified by FC (eluent: EtOAc/n-hexane = 1: 20) to afford the product **3cg** (53.3 mg, yield: 80%) as a brown oil, ^{1}H NMR (500 MHz, Chloroform-*d*) δ 6.74 (d, *J* = 2.5 Hz, 1H), 6.65 (dd, *J* = 8.4, 2.6 Hz, 1H), 6.43 (d, *J* = 8.4 Hz, 1H), 3.74 (s, 3H), 3.31 (s, 0.13H), 3.23 (t, *J* = 8.0 Hz, 2H), 2.91 (t, *J* = 8.0 Hz, 2H); ^{13}C NMR (126 MHz, CDCl$_3$) δ 153.04, 147.83, 131.99, 111.90, 111.75, 107.85, 56.90, 56.02, 36.78, 29.08. HRMS (ESI) calcd for $C_{10}D_3H_{11}NO$ [M+H]$^+$: 167.1258, found 167.1256.

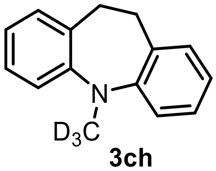

3ch

5-(methyl-d$_3$)-10,11-dihydro-5H-dibenzo[b,f]azepine

Purified by FC (eluent: EtOAc/n-hexane = 1: 100) to afford the product **3ch** (64.6 mg, yield: 76%) as a yellow solid, ^{1}H NMR (500 MHz, Chloroform-*d*) δ 7.14 (ddd, *J* = 8.7, 7.3, 1.8 Hz, 2H), 7.07 (dd, *J* = 7.5, 1.7 Hz, 2H), 7.04 (dd, *J* = 8.1, 1.3 Hz, 2H), 6.88 (td, *J* = 7.3, 1.2 Hz, 2H), 3.32 (t, *J* = 1.8 Hz, 0.22H), 3.18 – 3.08 (m, 4H); ^{13}C NMR (126 MHz, CDCl$_3$) δ 148.70, 133.11, 129.66, 126.38, 121.67, 118.63, 39.97-39.38 (m), 32.87. HRMS (ESI) calcd for $C_{15}H_{13}ND_3$ [M+H]$^+$: 213.1466, found 213.1463.

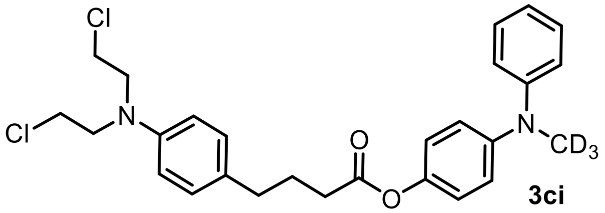

3ci

4-((methyl-d$_3$)(phenyl)amino)phenyl 4-(4-(bis(2-chloroethyl)amino)phenyl)butanoate

0.3 mmol scale. Purified by FC (eluent: EtOAc/n-hexane = 1: 20) to afford the product **3ci** (98.7 mg, yield: 68%) as a colorless oil, ^{1}H NMR (500 MHz, Chloroform-*d*) δ 7.29 – 7.22 (m, 1H), 7.11 (d, *J* = 8.6 Hz, 1H), 7.03 – 6.98 (m, 2H), 6.98 – 6.92 (m, 1H), 6.64 (d, *J* = 8.7 Hz, 1H), 3.70 (t, *J* = 6.7 Hz, 2H), 3.62 (t, *J* = 7.4 Hz, 2H), 3.26 (p, *J* = 1.8 Hz, 0H), 2.65 (t, *J* = 7.5 Hz, 1H), 2.55 (t, *J* = 7.4 Hz, 1H), 2.03 (p, *J* = 7.5 Hz, 0.08H); ^{13}C NMR (126 MHz, CDCl3) δ 172.43, 148.97, 146.73, 144.99, 144.43, 130.42, 129.78, 129.22, 122.14, 121.56, 121.14, 119.96, 112.22, 53.63, 40.54, 33.98, 33.68, 26.80. HRMS (ESI) calcd for $C_{27}\,D_3H_{28}N_2O_2Cl_2$ [M+H]$^+$: 488.1945, found 488.1958.

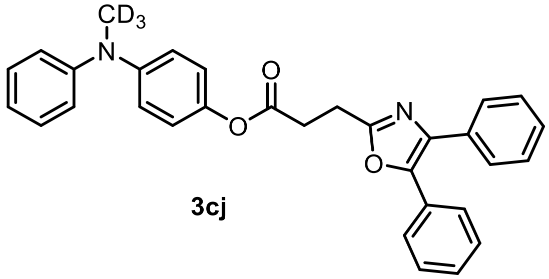

3cj

4-((methyl-d$_3$)(phenyl)amino)phenyl 3-(4,5-diphenyloxazol-2-yl)propanoate

0.3 mmol scale. Purified by FC (eluent: EtOAc/n-hexane = 1: 8) to afford the product **3cj** (118.8 mg, yield: 83%) as a colorless oil, ^{1}H NMR (500 MHz, Chloroform-*d*) δ 7.67 – 7.63 (m, 2H), 7.60 – 7.55 (m, 2H), 7.38 – 7.28 (m, 6H), 7.25 (dd, *J* = 8.6, 7.2 Hz, 2H), 7.03 – 6.96 (m, 6H), 6.93 (td, *J* = 7.3, 1.3 Hz, 1H), 3.29 (t, *J* = 7.3 Hz, 2H), 3.24 (p, *J* = 1.8 Hz, 0.09H), 3.14 (t, *J* = 7.3 Hz, 2H); ^{13}C NMR (126 MHz, $CDCl_3$) δ 171.03, 161.58, 148.97, 146.88, 145.61, 144.91, 135.21, 132.45, 129.26, 128.98, 128.70, 128.62, 128.56, 128.15, 127.94, 126.58, 122.14, 121.49, 121.25, 120.09, 39.66 (t, *J* = 21.4 Hz), 31.30, 23.62. HRMS (ESI) calcd for $C_{31}D_3H_{24}N_2O_3$ [M+H]$^+$: 478.2204, found 478.2212.

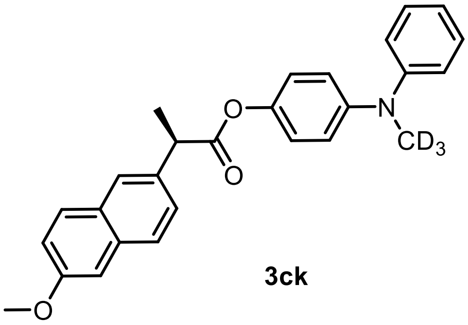

3ck

4-((methyl-d$_3$)(phenyl)amino)phenyl (R)-2-(6-methoxynaphthalen-2-yl)propanoate

0.3 mmol scale. Purified by FC (eluent: EtOAc/n-hexane = 1: 15) to afford the product **3ck** (95.0 mg, yield: 76%) as a white solid, ^{1}H NMR (500 MHz, Chloroform-*d*) δ 7.77 (d, *J* = 1.8 Hz, 1H), 7.74 (t, *J* = 8.1 Hz, 2H), 7.50 (dd, *J* = 8.5, 1.9 Hz, 1H), 7.23 (dd, *J* = 8.7, 7.3 Hz, 2H), 7.16 (dd, *J* = 8.9, 2.5 Hz, 1H), 7.13 (d, *J* = 2.5 Hz, 1H), 6.96 (td, *J* = 6.3, 3.1 Hz, 4H), 6.93 – 6.90 (m, 1H), 6.90 – 6.87 (m, 2H), 4.08 (q, *J* = 7.1 Hz, 1H), 3.92 (s, 3H), 3.25 – 3.21 (m, 0.15H), 1.69 (d, *J* = 7.1 Hz, 3H); ^{13}C NMR (151 MHz, $CDCl_3$) δ 173.38, 157.61, 148.83, 146.56, 145.09, 135.13, 133.69, 129.21, 129.05, 128.88, 127.23, 126.03, 126.01, 121.86, 121.53, 120.87, 119.62, 118.97, 105.50, 55.19, 45.44, 39.52 (q, *J* = 21.1 Hz), 18.46. HRMS (ESI) calcd for $C_{27}D_3H_{23}NO_3$ $[M+H]^+$: 415.2096, found 415.2103.

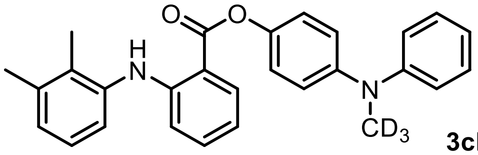

3cl

4-((methyl-d$_3$)(phenyl)amino)phenyl 2-((2,3-dimethylphenyl)amino)benzoate

0.3 mmol scale. Purified by FC (eluent: EtOAc/n-hexane = 1: 15) to afford the product **3cl** (103.0 mg, yield: 81%) as a yellow solid, ^{1}H NMR (600 MHz, Chloroform-*d*) δ 9.25 (s, 1H), 8.24 (dd, *J* = 8.1, 1.7 Hz, 1H), 7.37 – 7.35 (m, 1H), 7.35 – 7.30 (m, 2H), 7.21 (d, *J* = 7.9 Hz, 1H), 7.19 – 7.16 (m, 2H), 7.16 – 7.12 (m, 2H), 7.10 – 7.06 (m, 3H), 7.00 (t, *J* = 7.4 Hz, 1H), 6.85 – 6.82 (m, 1H), 6.81 – 6.75 (m, 1H), 3.34 (p, *J* = 1.8 Hz, 0.11H), 2.37 (s, 3H), 2.21 (s, 3H); ^{13}C

NMR (151 MHz, $CDCl_3$) δ 167.78, 150.25, 149.00, 146.88, 144.99, 138.45, 138.29, 134.90, 132.64, 131.91, 129.25, 127.03, 125.98, 123.29, 122.60, 121.68, 121.19, 120.05, 116.19, 113.81, 109.81, 20.65, 14.06. HRMS (ESI) calcd for $C_{28}D_3H_{24}N_2O_2$ $[M+H]^+$: 426.2255, found 426.2271.

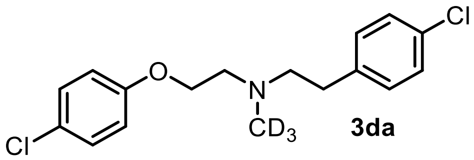

3da

N-(4-chlorophenethyl)-2-(4-chlorophenoxy)-N-(methyl-d$_3$)ethan-1-amine

Purified by FC (eluent: DCM/methnol = 20: 1) to afford the product **3da** (125.5 mg, yield: 96%) as a white solid, ^{1}H NMR (500 MHz, Chloroform-*d*) δ 7.26 – 7.21 (m, 4H), 7.16 – 7.10 (m, 2H), 6.83 – 6.79 (m, 2H), 4.12 (t, *J* = 5.5 Hz, 2H), 3.00 (t, *J* = 5.5 Hz, 2H), 2.91 – 2.78 (m, 4H), 2.47 (s, 0.06H).; ^{13}C NMR (126 MHz, $CDCl_3$) δ 157.02, 137.76, 132.12, 130.07, 129.39, 128.63, 125.96, 115.84, 65.71, 59.15, 55.65, 41.60 (d, *J* = 20.8 Hz), 32.37. HRMS (ESI) calcd for $C_{17}D_3H_{17}NOCl_2$ $[M+H]^+$: 327.1105, found 327.1114.

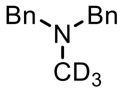

3db

N,N-dibenzylmethanamine-d$_3$

Purified by FC (eluent: EtOAc/n-hexane = 1: 10) to afford the product **3db** (62.6 mg, yield: 73%) as a yellow oil, ^{1}H NMR (500 MHz, Chloroform-*d*) δ 7.38 – 7.35 (m, 4H), 7.32 (dd, *J* = 8.5, 6.7 Hz, 4H), 7.28 – 7.21 (m, 2H), 3.52 (s, 4H); ^{13}C NMR (126 MHz, $CDCl_3$) δ 139.25, 128.95, 128.23, 126.94, 61.79. HRMS (ESI) calcd for $C_{15}H_{15}ND_3$ $[M+H]^+$: 215.1622, found 215.1625.

3e

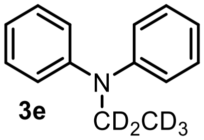

N-(ethyl-d$_5$)-N-phenylaniline

Purified by FC (eluent: EtOAc/n-hexane = 1: 100) to afford the product **3e** (43.8 mg, yield: 54%) as a colorless oil, ^{1}H NMR (500 MHz, Chloroform-*d*) δ 7.29 – 7.23 (m, 1H), 6.99 (d, *J* = 7.9 Hz, 1H), 6.93 (t, *J* = 7.3 Hz, 1H); ^{13}C NMR (126 MHz, $CDCl_3$) δ 147.73, 129.25, 121.06, 120.88. HRMS (ESI) calcd for $C_{14}D_5H_{11}N$ $[M+H]^+$: 203.1591, found 203.1596.

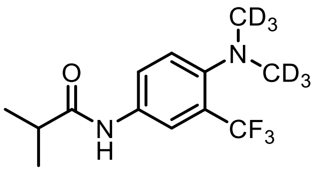

4a

N-(4-(bis(methyl-d$_3$)amino)-3-(trifluoromethyl)phenyl)isobutyramide

0.3 mmol scale. Purified by FC (eluent: EtOAc/n-hexane = 8: 1) to afford the product **4a** (59.9 mg, yield: 71%) as a yellow solid, ^{1}H NMR (500 MHz, Chloroform-*d*) δ 7.86 (s, 1H), 7.75 (dt, *J* = 8.9, 3.1 Hz, 1H), 7.68 (t, *J* = 3.1 Hz, 1H), 7.26 (dd, *J* = 8.6, 3.4 Hz, 1H), 2.62 (d, *J* = 3.2 Hz, 0.13H), 2.53 (ddp, *J* = 10.2, 6.8, 3.4 Hz, 1H), 1.23 (dd, *J* = 6.9, 3.4 Hz, 6H); ^{13}C NMR (126 MHz, $CDCl_3$) δ 175.93, 149.82, 134.00, 123.77 (q, *J* = 272.5Hz), 126.28 (q, *J* = 30.0Hz) 124.49, 123.50, 119.06 (t, *J* = 7.5Hz), 45.14, 44.98, 39.59 (t, *J* = 20.8 Hz), 36.40. HRMS (ESI) calcd for $C_{13}D_6H_{12}N_2OF_3$ $[M+H]^+$: 281.1742, found 281.1749.

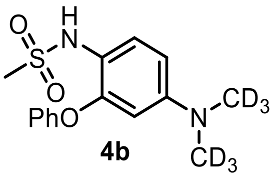

4b

N-(4-(bis(methyl-d$_3$)amino)-2-phenoxyphenyl)methanesulfonamide

0.3 mmol scale. Purified by FC (eluent: EtOAc/n-hexane = 8: 1) to afford the product **4b** (74.9 mg, yield: 80%) as a white solid, ^{1}H NMR (500 MHz, Chloroform-*d*) δ 7.43 (d, *J* = 8.9 Hz, 1H), 7.35 (dd, *J* = 8.6, 7.4 Hz, 1H), 7.19 – 7.06 (m, 1H), 7.03 – 6.92 (m, 1H), 6.48 (dd, *J* = 9.0, 2.8 Hz, 1H), 6.23 (t, *J* = 2.3 Hz, 2H), 2.89 (s, 3H), 2.83 (p, *J* = 1.9 Hz, 0.15H); ^{13}C NMR (126 MHz,

$CDCl_3$) δ 156.47, 150.34, 149.94, 130.07, 127.29, 123.74, 117.85, 116.23, 108.38, 102.79, 102.77, 39.42 (t, J = 19.5 Hz), 38.95. HRMS (ESI) calcd for $C_{15}D_6H_{13}N_2O_3S$ $[M+H]^+$: 313.1488, found 313.1503.

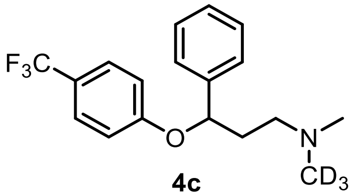

4c

N-methyl-N-(methyl-d$_3$)-3-phenyl-3-(4-(trifluoromethyl)phenoxy)propan-1-amine

0.3 mmol scale. Purified by FC (eluent: DCM/methnol = 20: 1) to afford the product **4c** (73.5 mg, yield: 75%) as a colorless oil, ^{1}H NMR (500 MHz, Chloroform-*d*) δ 7.42 (d, J = 8.7 Hz, 2H), 7.34 (d, J = 5.6 Hz, 4H), 7.27 (dt, J = 9.1, 2.8 Hz, 1H), 6.90 (d, J = 8.6 Hz, 2H), 5.32 (dd, J = 8.2, 4.8 Hz, 1H), 2.66 – 2.52 (m, 2H), 2.35 (s, 3H), 2.25 (dtd, J = 14.0, 8.4, 5.7 Hz, 1H), 2.14 – 2.05 (m, 1H); ^{13}C NMR (126 MHz, $CDCl_3$) δ 160.44, 140.63, 128.84, 127.98, 126.76 (q, J = 3.8Hz), 125.82, 124.42 (q, J = 267.5Hz). 123.00 (q, J = 34.0Hz), 115.78, 78.23, 55.49, 44.92, 44.29, 36.00. HRMS (ESI) calcd for $C_{18}D_3H_{18}NOF_3$ $[M+H]^+$: 327.1758, found 327.1772.

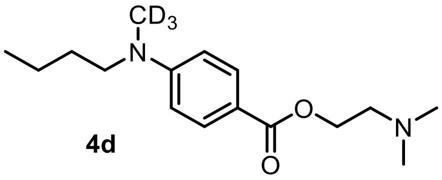

4d

2-(dimethylamino)ethyl 4-(butyl(methyl-d$_3$)amino)benzoate

0.3 mmol scale. Purified by FC (eluent: DCM/methnol = 20: 1) to afford the product **4d** (50.7 mg, yield: 60%) as a colorless oil, ^{1}H NMR (500 MHz, Chloroform-*d*) δ 7.89 (d, J = 9.0 Hz, 2H), 6.61 (d, J = 9.0 Hz, 2H), 4.38 (t, J = 5.9 Hz, 2H), 3.41 – 3.27 (m, 2H), 2.97 (p, J = 1.9 Hz, 0.07H), 2.71 (t, J = 5.9 Hz, 2H), 2.35 (s, 6H), 1.63 – 1.47 (m, 2H), 1.35 (h, J = 7.4 Hz, 2H), 0.95 (t, J = 7.3 Hz, 3H); ^{13}C NMR (126 MHz, $CDCl_3$) δ 166.91, 152.40, 131.46, 116.40, 110.37,

62.24, 57.99, 52.05, 45.85, 28.98, 20.27, 13.96. HRMS (ESI) calcd for $C_{16}D_3H_{24}N_2O_2$ $[M+H]^+$: 282.2255, found 282.2263.

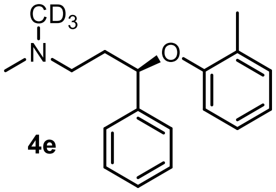

4e

(R)-N-methyl-N-(methyl-d$_3$)-3-phenyl-3-(o-tolyloxy)propan-1-amine

0.3 mmol scale. Purified by FC (eluent: DCM/methnol = 20: 1) to afford the product **4e** (70.1 mg, yield: 86%) as a colorless oil, ^{1}H NMR (600 MHz, Chloroform-*d*) δ 7.39 – 7.32 (m, 1H), 7.28 (d, *J* = 3.8 Hz, 0H), 7.17 – 7.10 (m, 0H), 7.04 – 6.92 (m, 0H), 6.81 (t, *J* = 7.4 Hz, 0H), 6.64 – 6.60 (m, 0H), 5.32 (dd, *J* = 8.1, 4.5 Hz, 0H), 2.89 – 2.79 (m, 2H), 2.52 (s, 3H), 2.38-2.35 (m, 1H), 2.33 (s, 3H), 2.30-2.25 (m, 1H), 2.07 (d, *J* = 1.1 Hz, 0.1H); ^{13}C NMR (151 MHz, $CDCl_3$) δ 155.61, 140.98, 130.70, 128.76, 127.83, 126.84, 126.68, 125.75, 120.54, 112.78, 77.26, 55.47, 44.22, 43.47, 35.08, 16.59. HRMS (ESI) calcd for $C_{18}D_3H_{21}NO$ $[M+H]^+$: 273.2041, found 273.2053.

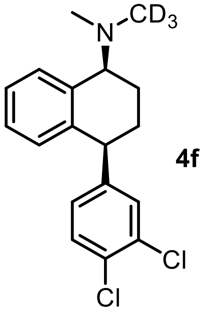

4f

(1S,4S)-4-(3,4-dichlorophenyl)-N-methyl-N-(methyl-d$_3$)-1,2,3,4-tetrahydronaphthalen-1-amine

Purified by FC (eluent: DCM/methnol = 20: 1) to afford the product **4f** (90.0 mg, yield: 70%) as a yellow oil, ^{1}H NMR (500 MHz, Chloroform-*d*) δ 7.70 (d, *J* = 7.6 Hz, 1H), 7.31 (d, *J* = 8.2 Hz,

1H), 7.25 – 7.22 (m, 1H), 7.17 – 7.12 (m, 2H), 6.88 (dd, *J* = 7.7, 1.3 Hz, 1H), 6.85 (dd, *J* = 8.3, 2.1 Hz, 1H), 4.11 (t, *J* = 5.2 Hz, 1H), 3.77 (dd, *J* = 8.4, 6.2 Hz, 1H), 2.30 (s, 3H), 2.14 – 2.08 (m, 1H), 2.06 – 2.01 (m, 1H), 1.72 – 1.67 (m, 2H); ^{13}C NMR (126 MHz, $CDCl_3$) δ 147.68, 139.06, 138.20, 132.10, 130.77, 130.16, 129.98, 129.82, 128.94, 128.21, 126.91, 126.75, 62.54, 43.89, 40.87, 29.71, 15.97. HRMS (ESI) calcd for $C_{18}D_3H_{17}NCl_2$ [M+H]$^+$: 323.1156, found 323.1162.

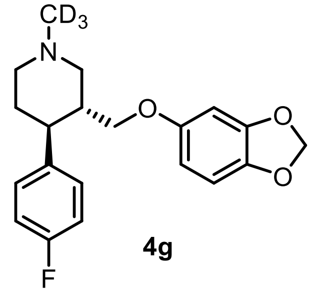

4g

(3R,4S)-3-((benzo[d][1,3]dioxol-5-yloxy)methyl)-4-(4-fluorophenyl)-1-(methyl-d$_3$)piperidine

0.3 mmol scale. Purified by FC (eluent: DCM/methnol = 20: 1) to afford the product **4g** (97.5 mg, yield: 94%) as a brown solid, ^{1}H NMR (600 MHz, Chloroform-*d*) δ 7.26 – 7.19 (m, 2H), 7.05 – 6.94 (m, 2H), 6.64 (ddd, *J* = 8.5, 3.0, 1.9 Hz, 1H), 6.39 – 6.32 (m, 1H), 6.14 (dq, *J* = 8.4, 2.3 Hz, 1H), 5.96 – 5.80 (m, 2H), 3.60 (dd, *J* = 9.5, 2.0 Hz, 1H), 3.53 – 3.47 (m, 2H), 3.45 (d, *J* = 12.6 Hz, 1H), 2.81 – 2.69 (m, 3H), 2.68 – 2.63 (m, 1H), 2.53 – 2.42 (m, 1H), 1.98 (d, *J* = 14.2 Hz, 1H); ^{13}C NMR (151 MHz, $CDCl_3$) δ 161.76 (d, *J* = 246.1 Hz), 160.94, 153.80, 148.18, 141.90, 137.46 (d, *J* = 4.5 Hz), 129.00 (d, *J* = 9.0 Hz), 115.65 (d, *J* = 21.1 Hz), 107.86, 105.47, 101.15, 97.88, 67.91, 57.67, 55.11, 44.08, 43.93, 43.79 (q, *J* = 21.1 Hz), 41.53, 40.27, 31.41. HRMS (ESI) calcd for $C_{20}D_3H_{19}NO_3F$ [M+H]$^+$: 347.1845, found 347.1862.

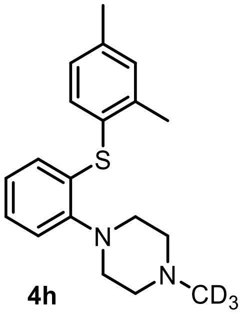

4h

1-(2-((2,4-dimethylphenyl)thio)phenyl)-4-(methyl-d$_3$)piperazine

Purified by FC (eluent: DCM/methnol = 20: 1) to afford the product **4h** (113.5 mg, yield: 90%) as a colorless oil, ^{1}H NMR (600 MHz, Chloroform-*d*) δ 7.39 (d, *J* = 7.8 Hz, 1H), 7.18 – 7.15 (m, 1H), 7.14 – 7.07 (m, 2H), 7.06 – 7.03 (m, 1H), 6.90 (ddd, *J* = 8.3, 6.7, 2.0 Hz, 1H), 6.52 (dd, *J* = 7.9, 1.2 Hz, 1H), 3.26 (t, *J* = 4.9 Hz, 4H), 2.88 (s, 4H), 2.38 (s, 3H), 2.33 (s, 3H); ^{13}C NMR (151 MHz, $CDCl_3$) δ 148.38, 142.41, 139.35, 136.20, 134.52, 131.73, 127.85, 127.65, 126.15, 125.59, 124.78, 120.10, 55.07, 50.57, 44.47 (d, *J* = 20.4 Hz). 21.20, 20.60. HRMS (ESI) calcd for $C_{19}D_3H_{22}N_2S$ [M+H]$^+$: 316.1921, found 316.1931.

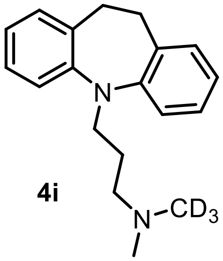

4i

3-(10,11-dihydro-5H-dibenzo[b,f]azepin-5-yl)-N-methyl-N-(methyl-d$_3$)propan-1-amine

Purified by FC (eluent: DCM/methnol = 20: 1) to afford the product **4i** (104.0 mg, yield: 92%) as a white solid, ^{1}H NMR (500 MHz, Chloroform-*d*) δ 7.12 (td, *J* = 7.5, 6.9, 1.5 Hz, 2H), 7.08 (td, *J* = 6.2, 5.5, 2.8 Hz, 4H), 6.90 (td, *J* = 7.3, 1.4 Hz, 2H), 3.77 (t, *J* = 6.9 Hz, 2H), 3.15 (d, *J* = 5.3 Hz, 4H), 2.33 (t, *J* = 7.3 Hz, 2H), 2.16 (s, 3H), 1.73 (p, *J* = 7.1 Hz, 2H); ^{13}C NMR (126 MHz, $CDCl_3$) δ 148.27, 134.22, 129.79, 126.37, 122.43, 120.00, 57.54, 48.79, 45.31, 32.23, 26.00. HRMS (ESI) calcd for $C_{19}D_3H_{22}N_2$ [M+H]$^+$: 284.2201, found 284.2209.

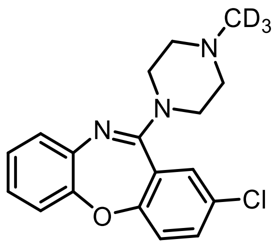

4j

2-chloro-11-(4-(methyl-d$_3$)piperazin-1-yl)dibenzo[b,f][1,4]oxazepine

0.3 mmol scale. Purified by FC (eluent: DCM/methnol = 20: 1) to afford the product **4j** (92.9 mg, yield: 94%) as a white solid, ^{1}H NMR (600 MHz, Chloroform-*d*) δ 7.41 (dd, *J* = 8.6, 2.6 Hz, 1H), 7.33 (d, *J* = 2.6 Hz, 1H), 7.20 (d, *J* = 8.7 Hz, 1H), 7.16 (dd, *J* = 7.8, 1.7 Hz, 1H), 7.13 – 7.07 (m, 2H), 7.01 (td, *J* = 7.6, 1.7 Hz, 1H), 3.68 (s, 4H), 2.70 (s, 4H), 2.43 (s, 0.1H); ^{13}C NMR (151 MHz, CDCl$_3$) δ 159.30, 158.62, 151.77, 139.96, 132.70, 130.38, 129.02, 127.09, 125.84, 124.81, 124.77, 122.75, 120.13, 54.28, 47.17. HRMS (ESI) calcd for $C_{18}D_3H_{16}N_3OCl$ [M+H]$^+$: 331.1399, found 331.1412.

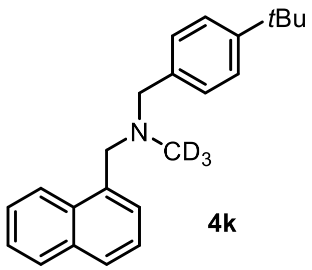

4k

N-(4-(tert-butyl)benzyl)-N-(naphthalen-1-ylmethyl)methanamine-d$_3$

Purified by FC (eluent: DCM/methnol = 20: 1) to afford the product **4k** (85.3 mg, yield: 67%) as a colorless oil, ^{1}H NMR (600 MHz, Chloroform-*d*) δ 8.31 – 8.25 (m, 1H), 7.88 (dd, *J* = 7.7, 1.8 Hz, 1H), 7.81 (d, *J* = 8.2 Hz, 1H), 7.57 – 7.48 (m, 3H), 7.45 (dd, *J* = 8.2, 6.9 Hz, 1H), 7.39 (d, *J* = 8.3 Hz, 2H), 7.34 (d, *J* = 8.3 Hz, 2H), 3.98 (s, 2H), 3.63 (s, 2H), 2.21 (s, 0.09H), 1.36 (s, 9H); ^{13}C NMR (151 MHz, CDCl$_3$) δ 149.88, 136.28, 135.08, 133.92, 132.57, 128.84, 128.38, 127.90,

127.70, 127.42, 125.67, 125.56, 125.27, 125.12, 125.10, 124.92, 62.02, 60.35, 41.58 (d, J = 21.1 Hz), 34.50, 31.45. HRMS (ESI) calcd for $C_{23}D_3H_{25}N$ [M+H]$^+$: 321.2405, found 321.2419.

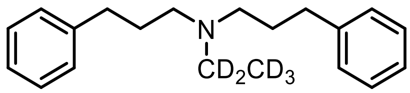

4l

N-(ethyl-d$_5$)-3-phenyl-N-(3-phenylpropyl)propan-1-amine

0.3 mmol scale. Purified by FC (eluent: DCM/methnol = 20: 1) to afford the product **2d** (72.1 mg, yield: 84%) as a colorless oil, ^{1}H NMR (600 MHz, Chloroform-*d*) δ 7.29 – 7.25 (m, 4H), 7.22 – 7.18 (m, 2H), 7.15 – 7.12 (m, 4H), 2.92 – 2.79 (m, 4H), 2.65 (t, J = 7.3 Hz, 4H), 2.11 – 1.94 (m, 4H); ^{13}C NMR (151 MHz, $CDCl_3$) δ 139.60, 128.69, 128.29, 126.56, 51.05, 32.71, 24.77. HRMS (ESI) calcd for $C_{20}D_5H_{23}N$ [M+H]$^+$: 287.2530, found 287.2539.

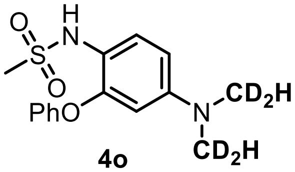

4o

N-(4-(bis(methyl-d$_2$)amino)-2-phenoxyphenyl)methanesulfonamide

Synthesized following the general procedure except using CH_3OD and D_2O. Purified by FC (eluent: EtOAc/n-hexane = 8: 1) to afford the product **4o** (95.4 mg, yield: 77%) as a white solid, ^{1}H NMR (600 MHz, Chloroform-*d*) δ 7.45 (d, J = 9.0 Hz, 1H), 7.37 (dd, J = 8.6, 7.4 Hz, 2H), 7.15 (td, J = 7.4, 1.1 Hz, 1H), 7.03 – 6.96 (m, 2H), 6.50 (dd, J = 9.0, 2.8 Hz, 1H), 6.26 (s, 1H), 2.91 (s, 3H), 2.90 (s, 0.25H), 2.86 (t, J = 2.0 Hz, 1.87H); ^{13}C NMR (151 MHz, $CDCl_3$) δ 156.47, 150.32, 149.95, 130.07, 127.29, 123.74, 117.87, 116.24, 108.40, 102.80, 39.89 (d, J = 21.1 Hz), 38.96. HRMS (ESI) calcd for $C_{15}D_4H_{15}N_2O_3S$ [M+H]$^+$: 311.1362, found 311.1373.

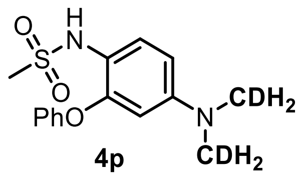

N-(4-(bis(methyl-d)amino)-2-phenoxyphenyl)methanesulfonamide

Synthesized following the general procedure except using $CD_3OD$and H_2O. Purified by FC (eluent: EtOAc/n-hexane = 8: 1) to afford the product **4p** (102.1 mg, yield: 83%) as a white solid, ^{1}H NMR (500 MHz, Chloroform-*d*) δ 7.44 (d, *J* = 8.9 Hz, 1H), 7.35 (dd, *J* = 8.7, 7.3 Hz, 2H), 7.16 – 7.11 (m, 1H), 7.01 – 6.96 (m, 2H), 6.49 (dd, *J* = 9.0, 2.8 Hz, 1H), 6.24 (d, *J* = 2.8 Hz, 1H), 2.89 (s, 3H), 2.87 (s, 0.97H), 2.86 – 2.85 (m, 3.02H); ^{13}C NMR (126 MHz, $CDCl_3$) δ 156.47, 149.91, 130.07, 127.26, 123.74, 117.84, 116.27, 108.43, 102.83, 40.19 (t, *J* = 21.4 Hz), 38.95. HRMS (ESI) calcd for $C_{15}D_2H_{17}N_2O_3S$ $[M+H]^+$: 309.1236, found 309.1250.

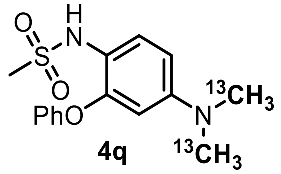

N-(4-(di(methyl-^{13}C)amino)-2-phenoxyphenyl)methanesulfonamide

Synthesized following the general procedure except using $^{13}CH_3OH$ and H_2O. 0.3 mmol scale. Purified by FC (eluent: EtOAc/n-hexane = 8: 1) to afford the product **4q** (78.7 mg, yield: 85%) as a white solid, ^{1}H NMR (600 MHz, Chloroform-*d*) δ 7.46 (d, *J* = 8.9 Hz, 1H), 7.38 (dd, *J* = 8.7, 7.4 Hz, 2H), 7.20 – 7.12 (m, 1H), 7.05 – 6.98 (m, 2H), 6.51 (dd, *J* = 9.0, 2.8 Hz, 1H), 6.29 (s, 1H), 6.27 (d, *J* = 2.8 Hz, 1H), 2.91 (s, 3H), 2.90 (d, J^{13}C-H = 138 Hz, 3 H), 2.89 (d, J^{13}C-H = 138 Hz, 3 H); ^{13}C NMR (151 MHz, $CDCl_3$) δ 156.47, 150.28, 149.96, 130.07, 127.29, 123.75, 117.88, 116.28, 108.45, 108.43, 108.42, 102.86, 102.84, 102.82, 40.49 (intense), 38.97. HRMS (ESI) calcd for $C_{13}H_{19}N_2O_3S^{13}C_2$ $[M+H]^+$: 309.1178, found 309.1175.

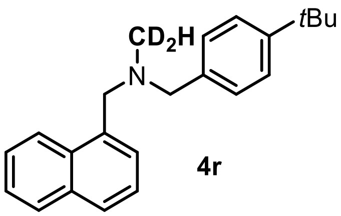

4r

N-(4-(tert-butyl)benzyl)-N-(naphthalen-1-ylmethyl)methanamine-d$_2$

Synthesized following the general procedure except using CD_3OD and H_2O. Purified by FC (eluent: DCM/methnol = 20: 1) to afford the product **4x** (95.0 mg, yield: 75%) as a colorless oil, ^{1}H NMR (500 MHz, Chloroform-*d*) δ 8.24 – 8.20 (m, 1H), 7.84 – 7.80 (m, 1H), 7.75 (d, *J* = 8.1 Hz, 1H), 7.47 (dd, *J* = 6.8, 2.9 Hz, 3H), 7.39 (t, *J* = 7.5 Hz, 1H), 7.33 (d, *J* = 8.0 Hz, 2H), 7.28 (d, *J* = 8.1 Hz, 2H), 3.92 (s, 2H), 3.57 (s, 2H), 2.15 (s, 1H), 1.31 (s, 9H); ^{13}C NMR (126 MHz, $CDCl_3$) δ 149.87, 136.30, 135.10, 133.92, 132.57, 128.83, 128.37, 127.89, 127.41, 125.67, 125.56, 125.12, 125.10, 124.92, 62.05, 60.40, 41.82 (t, *J* = 21.4 Hz), 34.50, 31.46. HRMS (ESI) calcd for $C_{23}D_2H_{26}N$ $[M+H]^+$: 320.2342, found 320.2354.

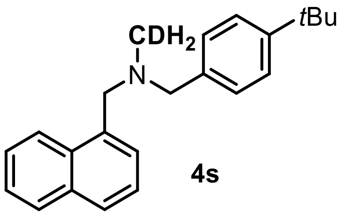

4s

N-(4-(tert-butyl)benzyl)-N-(methyl-d)-1-(naphthalen-1-yl)methanamine

Synthesized following the general procedure except using CH_3OD and D_2O. Purified by FC (eluent: DCM/methnol = 20: 1) to afford the product **4y** (89.0 mg, yield: 70%) as a colorless oil, ^{1}H NMR (600 MHz, Chloroform-*d*) δ 8.27 (d, *J* = 7.3 Hz, 1H), 7.89 (dd, *J* = 8.0, 1.6 Hz, 1H), 7.83 (d, *J* = 8.2 Hz, 1H), 7.57 (dd, *J* = 6.5, 4.1 Hz, 1H), 7.53 (qd, *J* = 7.1, 1.6 Hz, 2H), 7.47 (dd, *J* = 8.2, 7.0 Hz, 1H), 7.40 (d, *J* = 8.4 Hz, 2H), 7.36 (d, *J* = 8.1 Hz, 2H), 4.04 (s, 2H), 3.69 (s, 2H), 2.24 (s, 2H), 1.37 (s, 9H); ^{13}C NMR (151 MHz, $CDCl_3$) δ 149.88, 136.28, 135.08, 133.92, 132.57, 128.84, 128.38, 127.90, 127.70, 127.42, 125.67, 125.56, 125.27, 125.12, 125.10, 124.92, 62.02,

60.35, 42.11 (t, *J* = 21.1 Hz), 34.50, 31.45. HRMS (ESI) calcd for $C_{23}DH_{27}N$ $[M+H]^+$: 319.2279, found 319.2294.

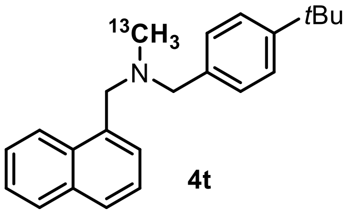

4t

N-(4-(tert-butyl)benzyl)-N-(methyl-^{13}C)-1-(naphthalen-1-yl)methanamine

Synthesized following the general procedure except using $^{13}CH_3OH$ and H_2O. 0.3 mmol scale. Purified by FC (eluent: DCM/methnol = 20: 1) to afford the product **4w** (71.5 mg, yield: 75%) as a light yellow oil, ^{1}H NMR (500 MHz, Chloroform-*d*) δ 8.22 (dd, *J* = 8.0, 1.8 Hz, 1H), 7.84 – 7.80 (m, 1H), 7.75 (d, *J* = 8.1 Hz, 1H), 7.50 – 7.44 (m, 3H), 7.39 (dd, *J* = 8.2, 7.0 Hz, 1H), 7.33 (dd, *J* = 8.4, 2.3 Hz, 2H), 7.31 – 7.26 (m, 2H), 3.92 (d, *J* = 4.8 Hz, 2H), 3.57 (d, *J* = 5.2 Hz, 2H), 2.20 (d, *J* = 133.1 Hz, 3H), 1.31 (s, 9H); ^{13}C NMR (126 MHz, $CDCl_3$) δ 162.65, 149.86, 136.29, 135.09, 133.91, 132.55, 128.81, 128.35, 127.87, 125.65, 125.54, 125.10, 125.08, 124.91, 62.07, 60.44, 42.41, 42.26 (intense), 34.48, 31.44. HRMS (ESI) calcd for $C_{22}H_{28}N^{13}C$ $[M+H]^+$: 319.2250, found 319.2247.

4u

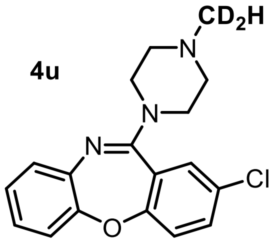

2-chloro-11-(4-(methyl-d$_2$)piperazin-1-yl)dibenzo[b,f][1,4]oxazepine

Synthesized following the general procedure except using CD_3OD and H_2O. Purified by FC (eluent: DCM/methnol = 20: 1) to afford the product **4u** (85.0 mg, yield: 87%) as a colorless oil, ^{1}H NMR (600 MHz, Chloroform-*d*) δ 7.44 (td, *J* = 7.7, 1.6 Hz, 0.20H), 7.40 (dd, *J* = 8.7, 2.6 Hz,

0.80H), 7.37 (dd, J = 7.7, 1.7 Hz, 0.20H), 7.33 (d, J = 2.6 Hz, 0.80H), 7.27 (dd, J = 8.2, 1.0 Hz, 0.21H), 7.20 (d, J = 8.7 Hz, 0.79H), 7.17 (dd, J = 7.9, 1.7 Hz, 1H), 7.15 – 7.06 (m, 2H), 7.00 (td, J = 7.6, 1.7 Hz, 1H), 3.57 (s, 4H), 2.56 (s, 4H), 2.34 (s, 1H); ^{13}C NMR (151 MHz, $CDCl_3$) δ 159.29, 158.84, 151.80, 140.11, 132.55, 130.26, 129.07, 127.09, 125.80, 124.98, 124.57, 122.71, 120.10, 54.72, 47.16. HRMS (ESI) calcd for $C_{18}D_2H_{17}N_3OCl$ $[M+H]^+$: 330.1337, found 330.1352.

4v

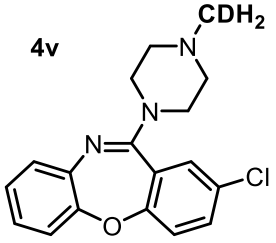

2-chloro-11-(4-(methyl-d)piperazin-1-yl)dibenzo[b,f][1,4]oxazepine

Synthesized following the general procedure except using CH_3OD and D_2O. Purified by FC (eluent: DCM/methnol = 20: 1) to afford the product **4v** (84.0 mg, yield: 86%) as a colorless oil, ^{1}H NMR (600 MHz, Chloroform-*d*) δ 7.39 (dd, J = 8.6, 2.6 Hz, 1H), 7.33 (d, J = 2.6 Hz, 1H), 7.19 (d, J = 8.6 Hz, 1H), 7.16 (dd, J = 7.8, 1.7 Hz, 1H), 7.13 – 7.07 (m, 2H), 7.00 (td, J = 7.6, 1.7 Hz, 1H), 3.59 (s, 4H), 2.59 (s, 4H), 2.40 (s, 0.32H), 2.38 (s, 1.70H); ^{13}C NMR (151 MHz, $CDCl_3$) δ 159.29, 158.84, 151.80, 140.11, 132.55, 130.26, 129.07, 127.09, 125.80, 124.98, 124.57, 122.71, 120.10, 54.72, 47.16, 45.78 (q, J = 20.4 Hz). HRMS (ESI) calcd for $C_{18}DH_{18}N_3OCl$ $[M+H]^+$: 329.1274, found 329.1290.

4w

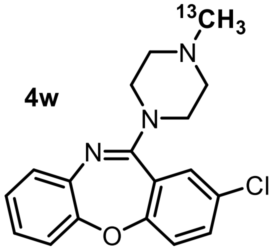

2-chloro-11-(4-(methyl-^{13}C)piperazin-1-yl)dibenzo[b,f][1,4]oxazepine

Synthesized following the general procedure except using $^{13}CH_3OH$ and H_2O. 0.3 mmol scale. Purified by FC (eluent: DCM/methnol = 20: 1) to afford the product **4w** (88.8 mg, yield: 90%) as a light yellow oil, ^1H NMR (600 MHz, Chloroform-*d*) δ 7.40 (dd, *J* = 8.7, 2.6 Hz, 1H), 7.33 (d, *J* = 2.6 Hz, 1H), 7.20 (d, *J* = 8.6 Hz, 1H), 7.17 (dd, *J* = 7.9, 1.7 Hz, 1H), 7.13 – 7.07 (m, 2H), 7.00 (td, *J* = 7.6, 1.7 Hz, 1H), 3.58 (s, 4H), 2.64 – 2.53 (m, 4H), 2.38 (d, J^{13}C-H = 138 Hz, 3 H); ^{13}C NMR (151 MHz, $CDCl_3$) δ 159.30, 158.89, 151.81, 140.15, 132.51, 130.24, 129.09, 127.09, 125.80, 125.03, 124.53, 122.70, 120.10, 54.83, 46.09 (intense). HRMS (ESI) calcd for $C_{17}H_{19}N_3OCl^{13}C$ $[M+H]^+$: 329.1245, found 329.1240.

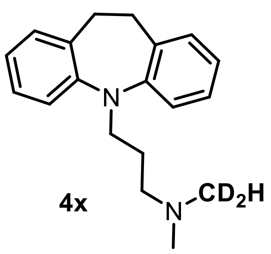

3-(10,11-dihydro-5H-dibenzo[b,f]azepin-5-yl)-N-methyl-N-(methyl-d$_{2)}$propan-1-amine

Synthesized following the general procedure except using CD_3OD and H_2O. 0.3 mmol scale. Purified by FC (eluent: DCM/methnol = 20: 1) to afford the product **4r** (75.0 mg, yield: 89%) as a white solid, ^1H NMR (600 MHz, Chloroform-*d*) δ 7.16 (td, *J* = 7.7, 1.6 Hz, 2H), 7.13 – 7.11 (m, 2H), 7.08 (d, *J* = 8.1 Hz, 2H), 6.99 – 6.93 (m, 2H), 3.86 (t, *J* = 6.3 Hz, 2H), 3.16 (s, 4H), 2.87 (dd, *J* = 9.2, 6.6 Hz, 2H), 2.52 (s, 3H), 2.49 (s, 1H), 2.14 – 2.00 (m, 2H); ^{13}C NMR (151 MHz, $CDCl_3$) δ 147.14, 133.71, 129.67, 126.30, 122.70, 119.39, 55.98, 47.26, 42.38 (p, *J* = 21.1 Hz), 42.23, 31.76, 22.71. HRMS (ESI) calcd for $C_{19}D_2H_{23}N_2$ $[M+H]^+$: 283.2138, found 283.2151.

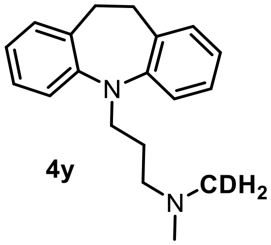

3-(10,11-dihydro-5H-dibenzo[b,f]azepin-5-yl)-N-methyl-N-(methyl-d)propan-1-amine

Synthesized following the general procedure except using CH_3OD and D_2O. 0.3 mmol scale. Purified by FC (eluent: DCM/methnol = 20: 1) to afford the product **4s** (77.9 mg, yield: 92%) as a white solid, ^{1}H NMR (600 MHz, Chloroform-*d*) δ 7.18 – 7.14 (m, 2H), 7.12 (dt, *J* = 7.5, 1.7 Hz, 2H), 7.07 (dt, *J* = 8.1, 1.4 Hz, 2H), 6.98 – 6.92 (m, 2H), 3.86 (td, *J* = 6.4, 2.1 Hz, 2H), 3.16 (d, *J* = 1.7 Hz, 4H), 2.90 (dd, *J* = 9.6, 6.4 Hz, 2H), 2.54 (s, 3H), 2.53 – 2.51 (m, 2.03H), 2.13 – 2.02 (m, 2H); ^{13}C NMR (151 MHz, $CDCl_3$) δ 147.33, 133.94, 129.92, 126.55, 122.96, 119.61, 56.15, 47.42, 42.95, 31.96, 22.76. HRMS (ESI) calcd for $C_{19}DH_{24}N_2$ $[M+H]^+$: 282.2075, found 282.2087.

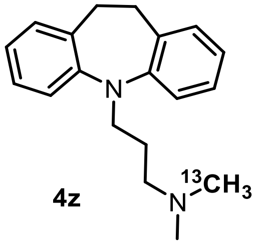

4z

3-(10,11-dihydro-5H-dibenzo[b,f]azepin-5-yl)-N-methyl-N-(methyl-^{13}C)propan-1-amine

Synthesized following the general procedure except using $^{13}CH_3OH$ and H_2O. 0.3 mmol scale. Purified by FC (eluent: DCM/methnol = 20: 1) to afford the product **4t** (76.1 mg, yield: 90%) as a light yellow oil, ^{1}H NMR (600 MHz, Chloroform-*d*) δ 7.15 – 7.11 (m, 2H), 7.10 (dd, *J* = 7.5, 1.7 Hz, 2H), 7.06 (dd, *J* = 8.1, 1.3 Hz, 2H), 6.93 (td, *J* = 7.3, 1.2 Hz, 2H), 3.81 (t, *J* = 6.6 Hz, 2H), 3.14 (s, 4H), 2.77 – 2.68 (m, 2H), 2.42 (d, J^{13}C-H = 138 Hz, 3 H), 2.44 – 2.38 (m, 3H), 2.02 – 1.88 (m, 2H); ^{13}C NMR (151 MHz, $CDCl_3$) δ 147.66, 134.10, 129.98, 126.60, 122.93, 119.80, 56.64, 47.86, 43.71 (intense), 32.14, 23.70. HRMS (ESI) calcd for $C_{18}{}^{13}CH_{25}N$ $[M+H]^+$: 282.2046, found 282.2041.

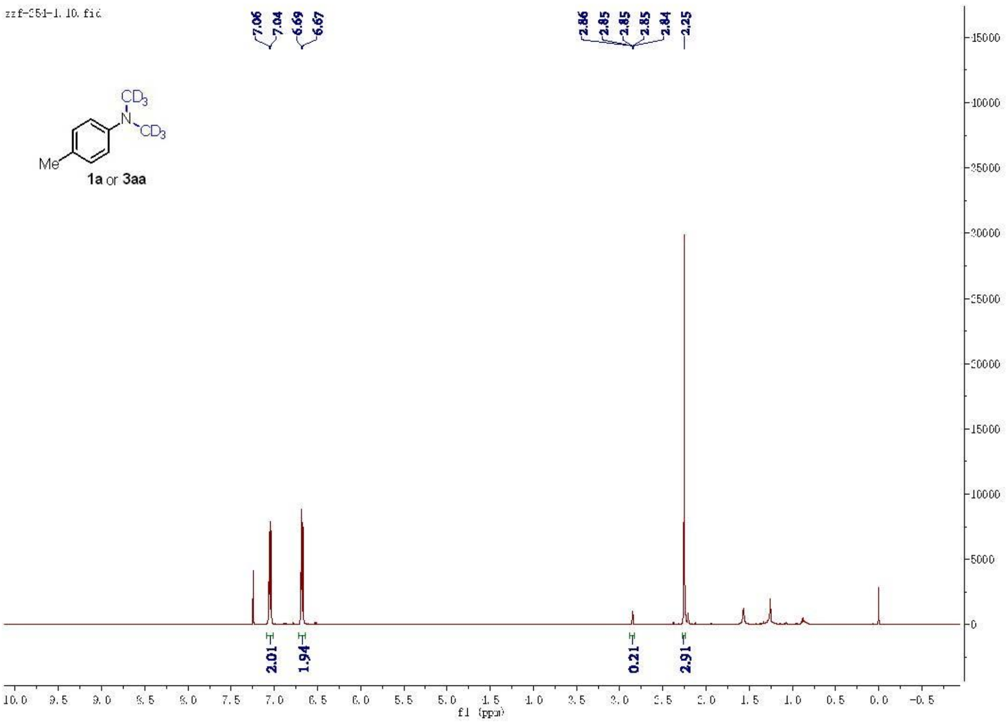

Supplementary Figure 11. ^{1}H NMR of product **1a**.

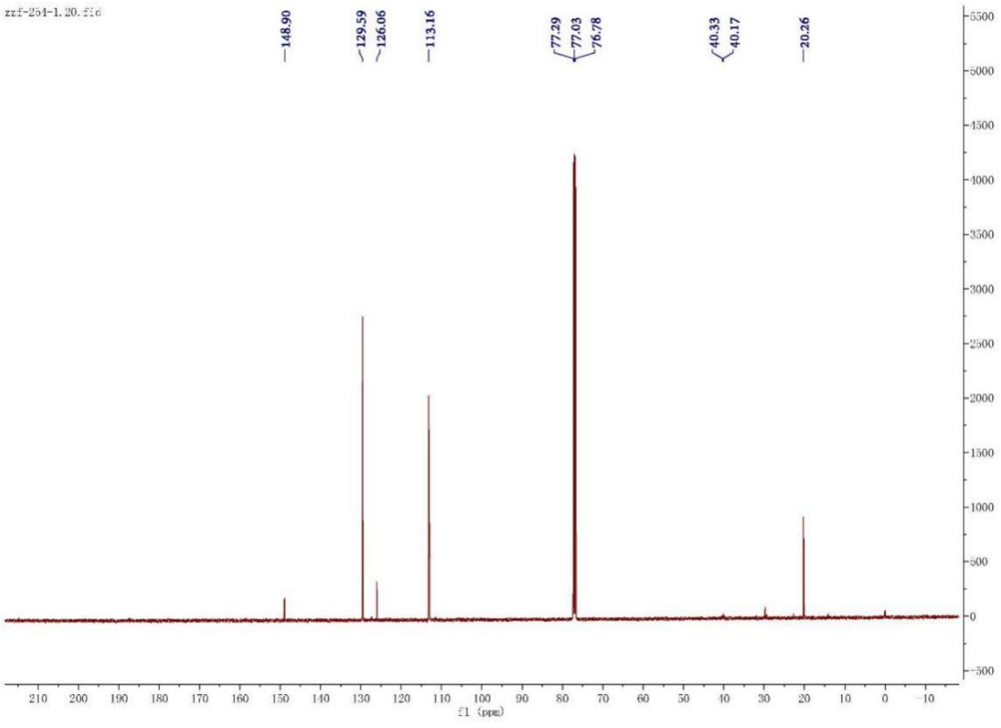

Supplementary Figure 12. ^{13}C NMR of product **1a**.

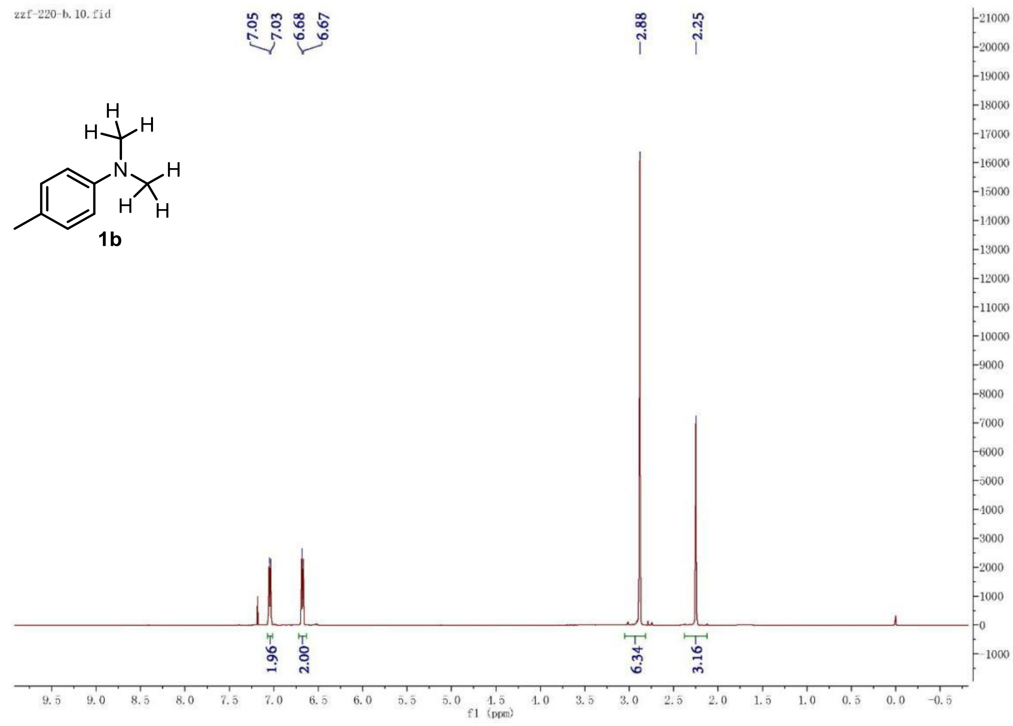

Supplementary Figure 13. ^{1}H NMR of product **1b**.

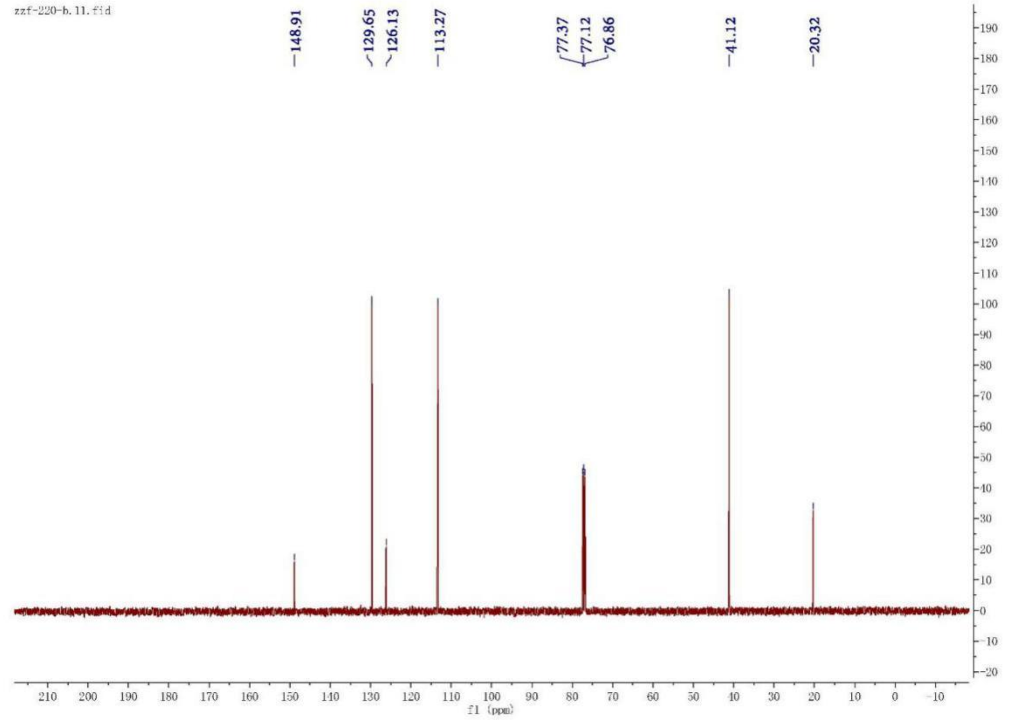

Supplementary Figure 14. ^{13}C NMR of product **1b**.

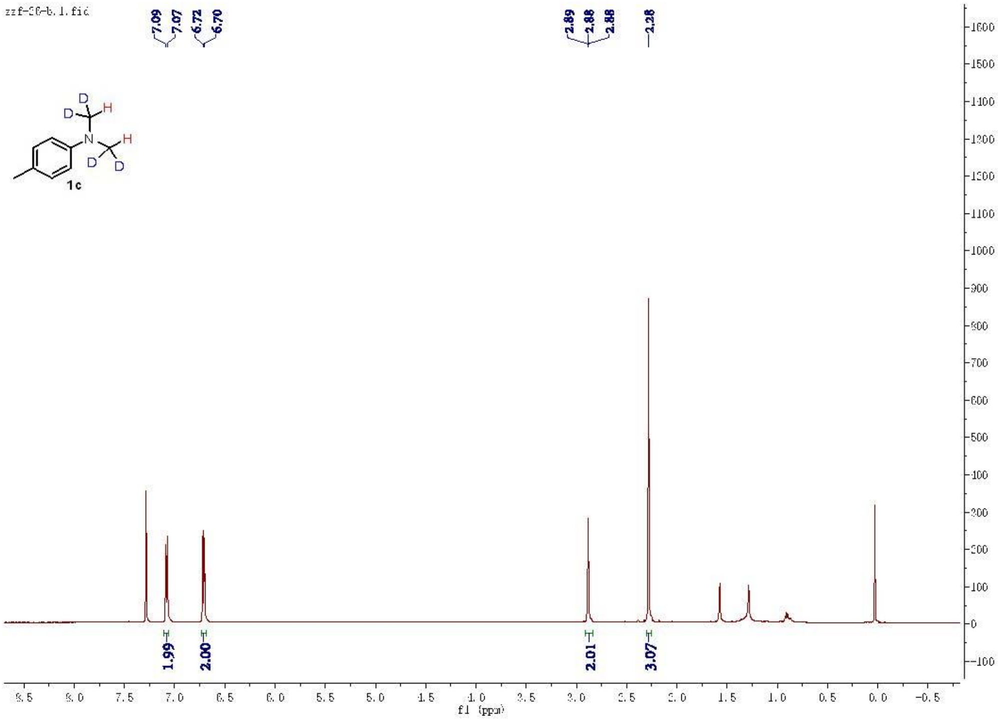

Supplementary Figure 15. ^{1}H NMR of product **1c**.

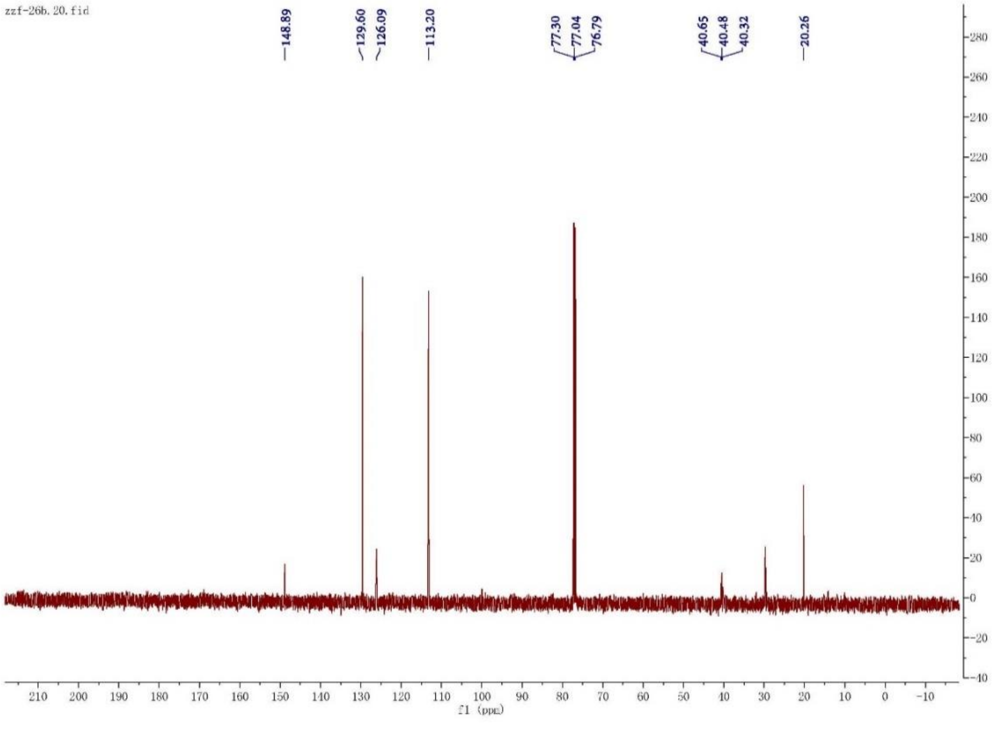

Supplementary Figure 16. ^{13}C NMR of product **1c**.

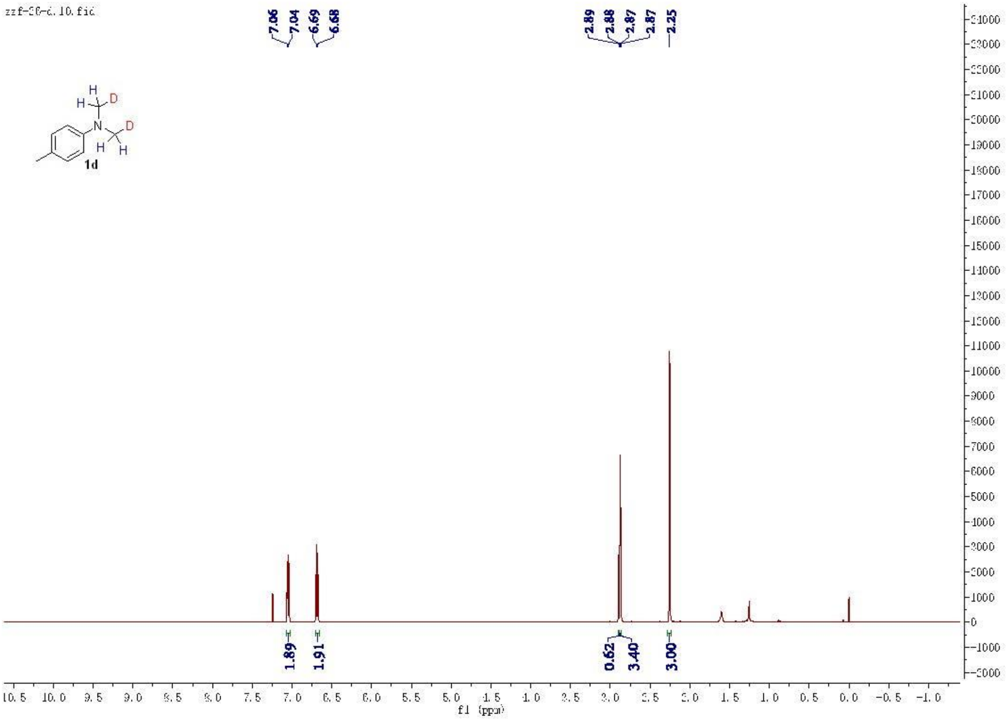

Supplementary Figure 17. ^{1}H NMR of product **1d**.

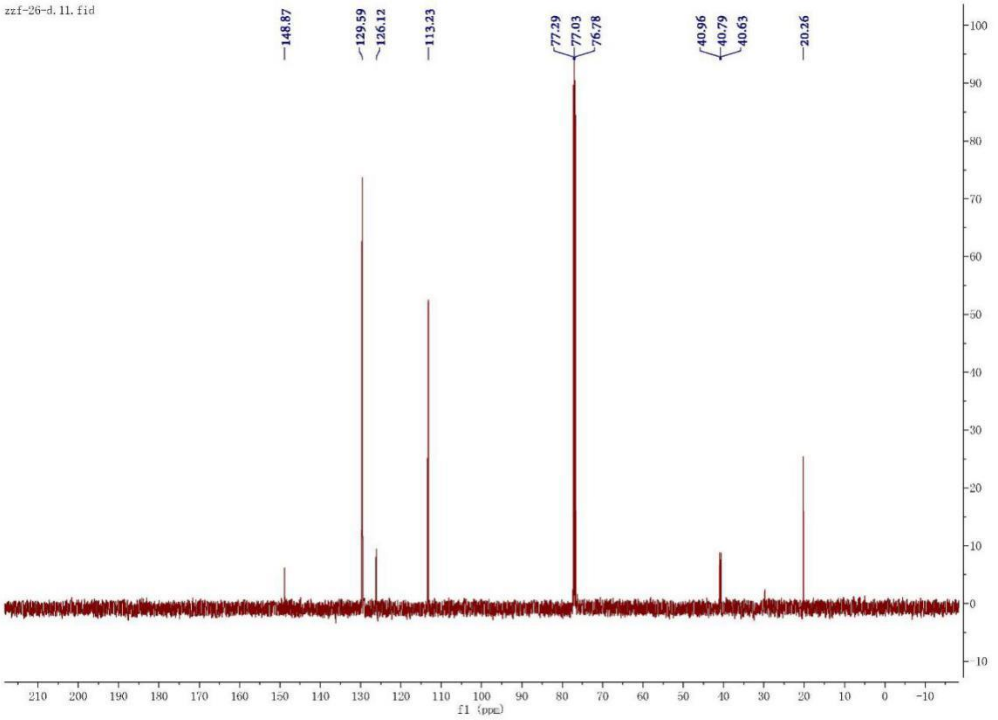

Supplementary Figure 18. ^{13}C NMR of product **1d**.

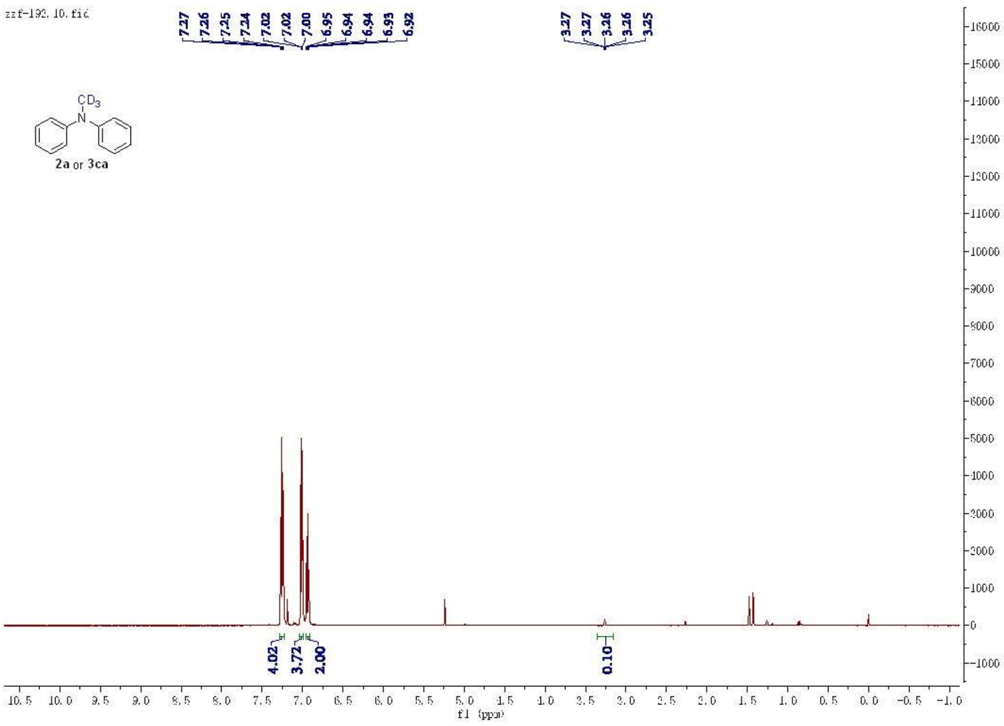

Supplementary Figure 19. ^{1}H NMR of product **2a**.

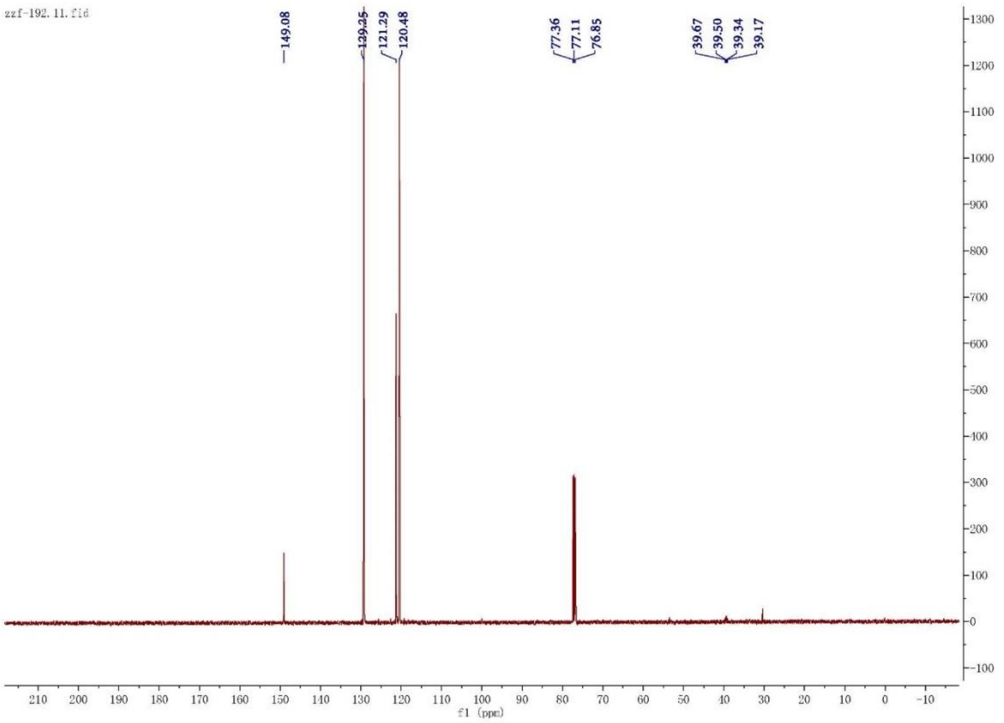

Supplementary Figure 20. ^{13}C NMR of product **2a**.

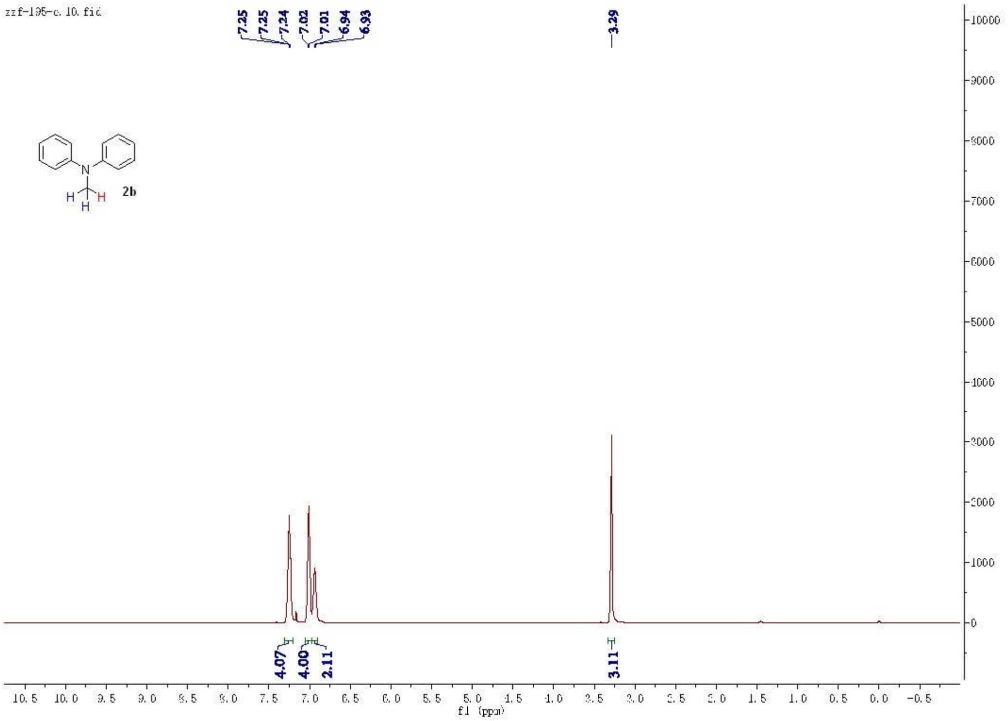

Supplementary Figure 21. ^{1}H NMR of product **2b**.

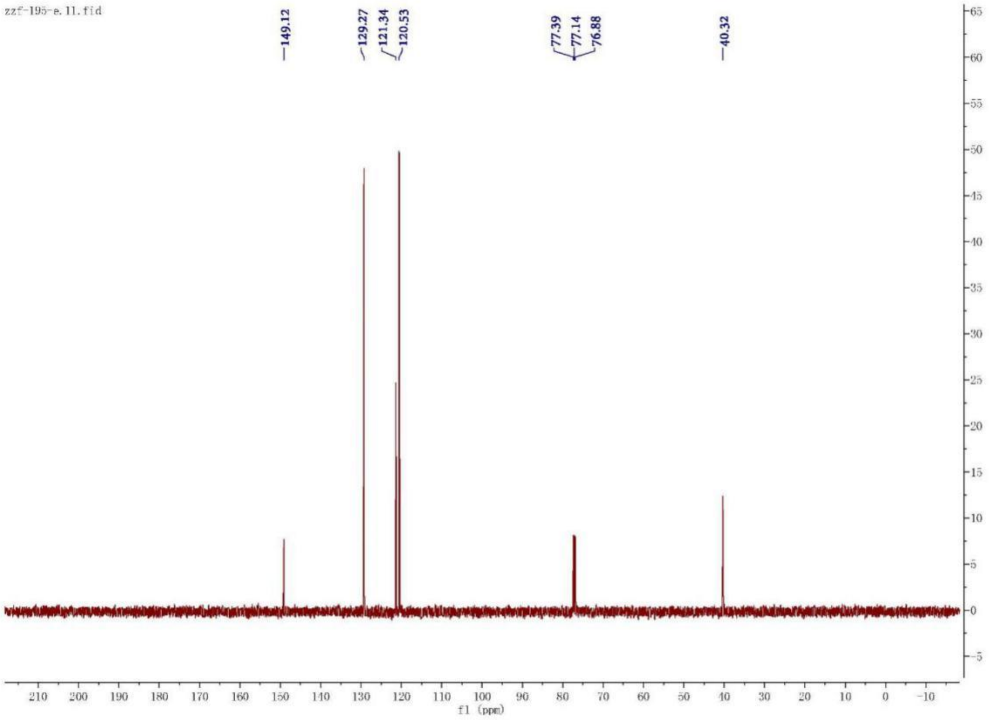

Supplementary Figure 22. ^{13}C NMR of product **2b**.

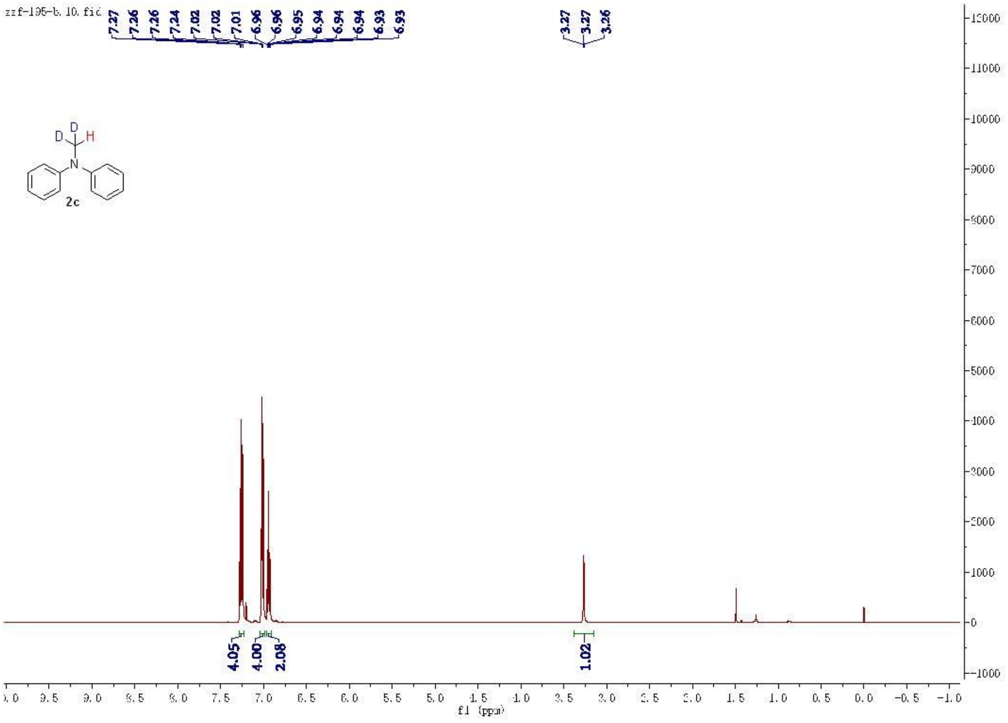

Supplementary Figure 23. ^{1}H NMR of product **2c**.

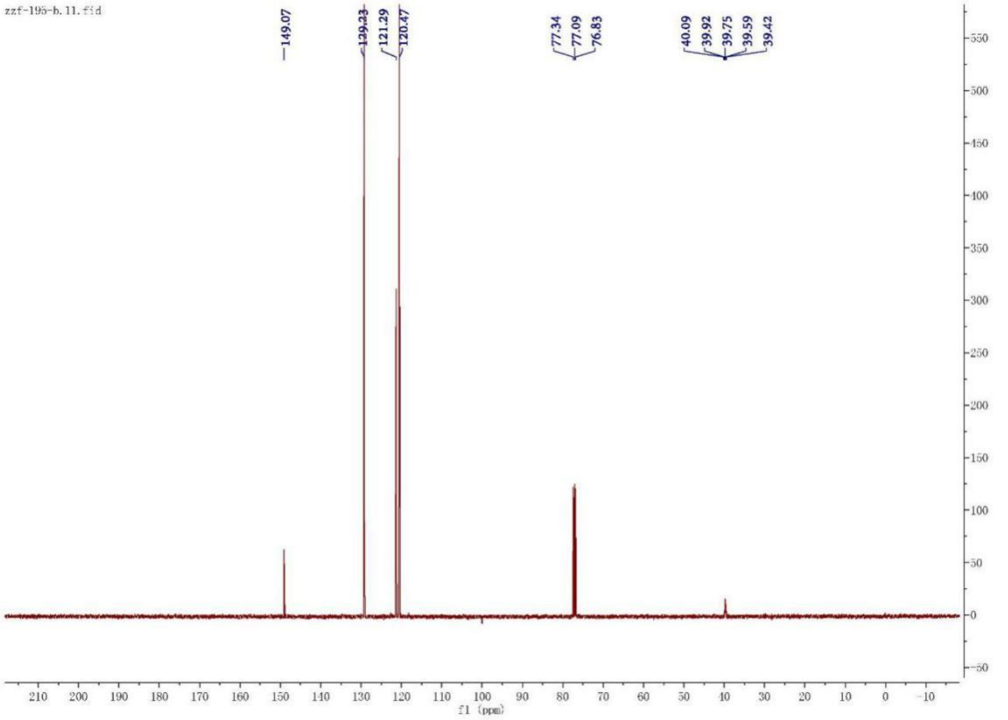

Supplementary Figure 24. ^{13}C NMR of product **2c**.

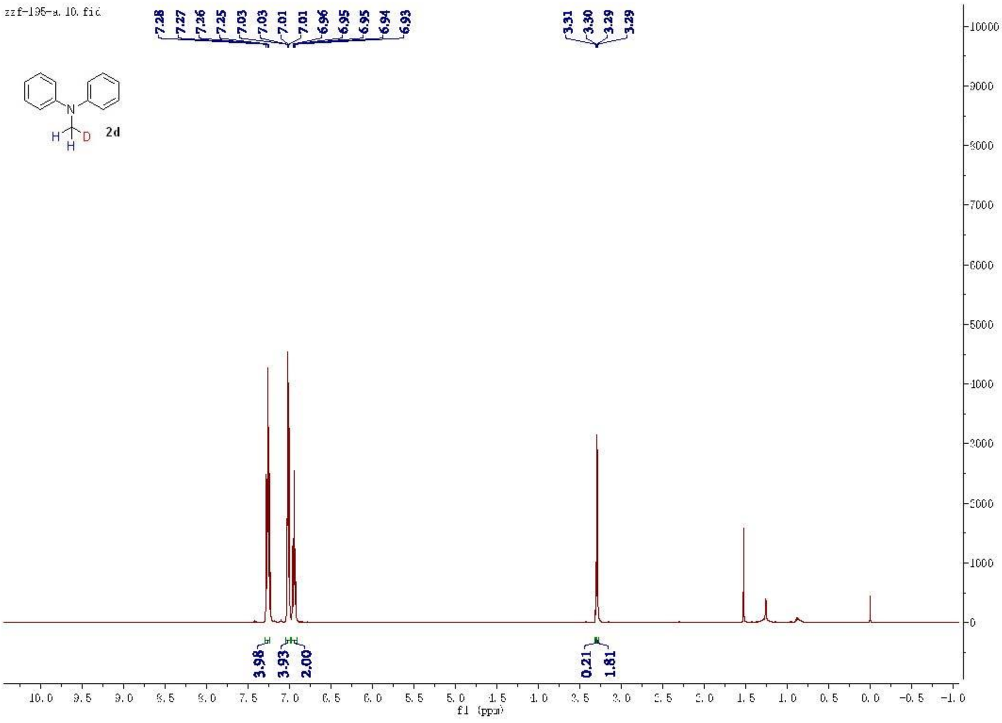

Supplementary Figure 25. ^{1}H NMR of product **2d**.

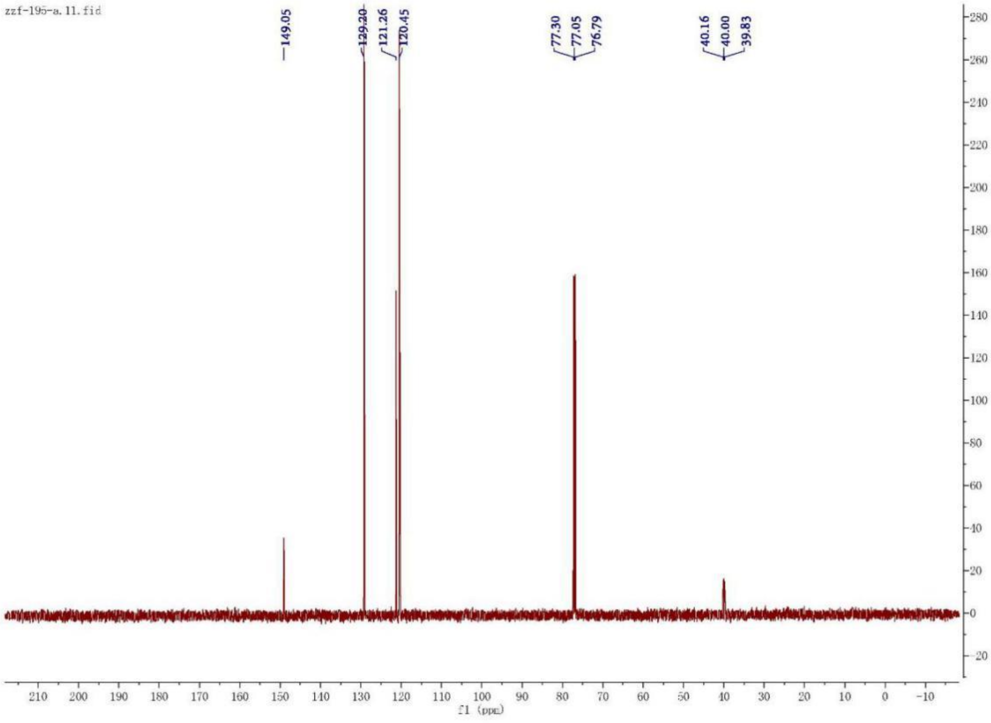

Supplementary Figure 26. ^{13}C NMR of product **2d**.

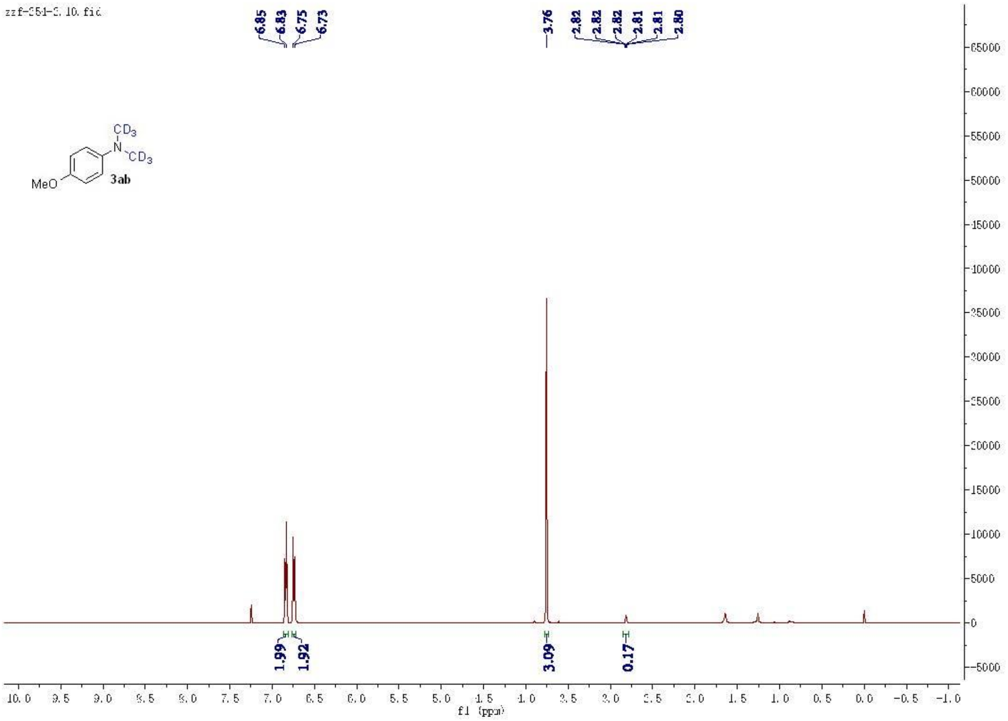

Supplementary Figure 27. ^{1}H NMR of product 3**ab**.

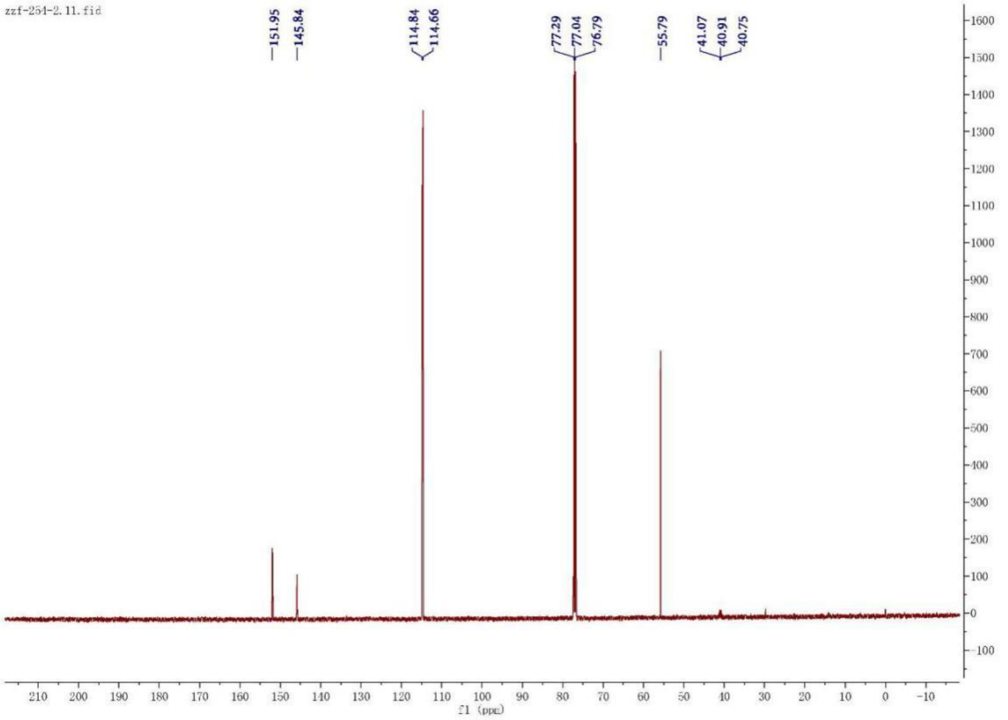

Supplementary Figure 28. ^{13}C NMR of product **3ab**.

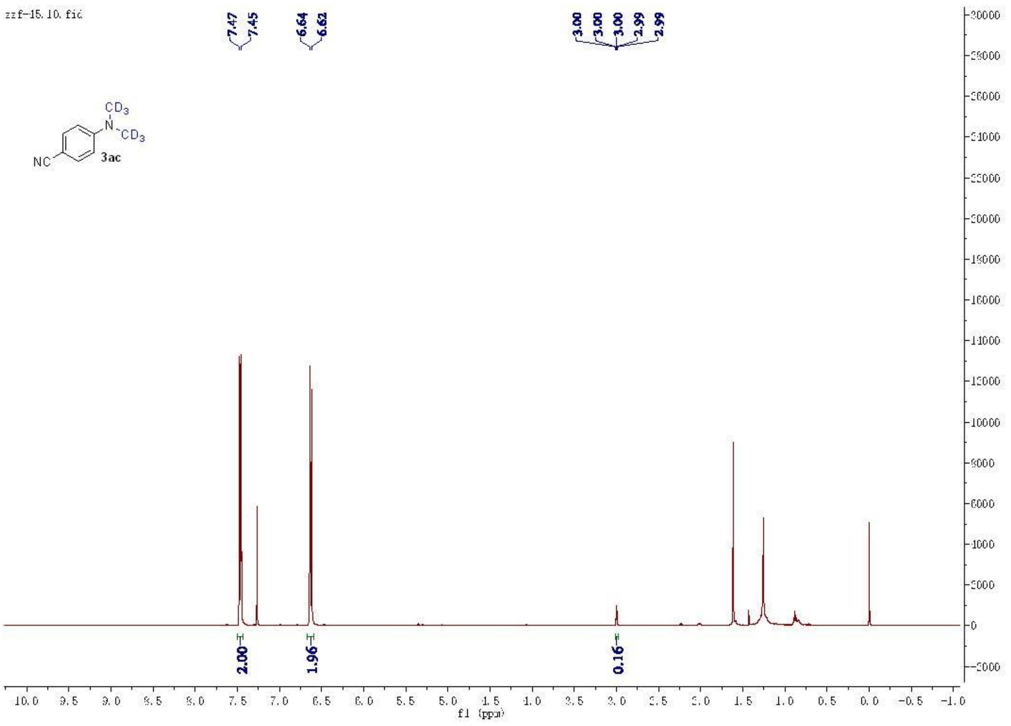

Supplementary Figure 29. ^{1}H NMR of product **3ac**.

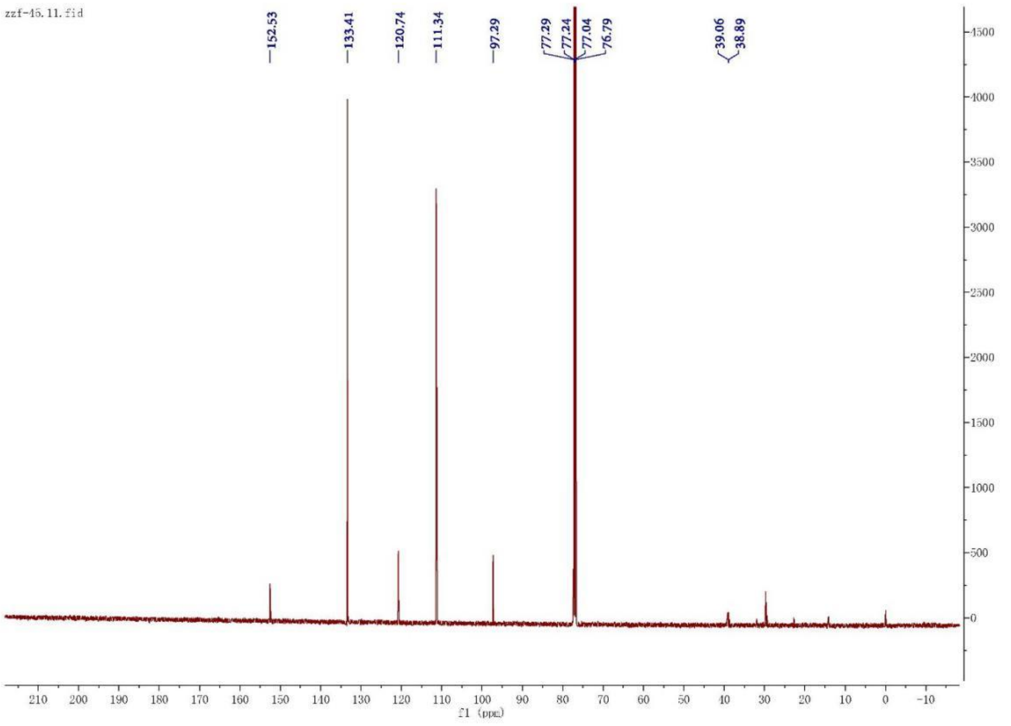

Supplementary Figure 30. ^{13}C NMR of product **3ac**.

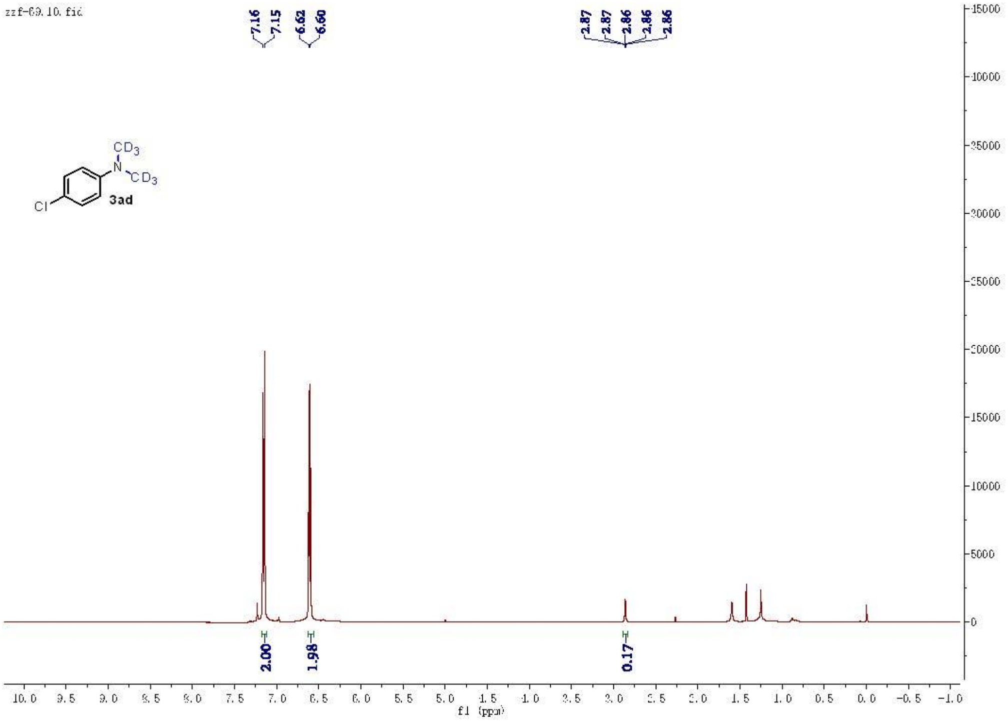

Supplementary Figure 31. ^{1}H NMR of product **3ad**.

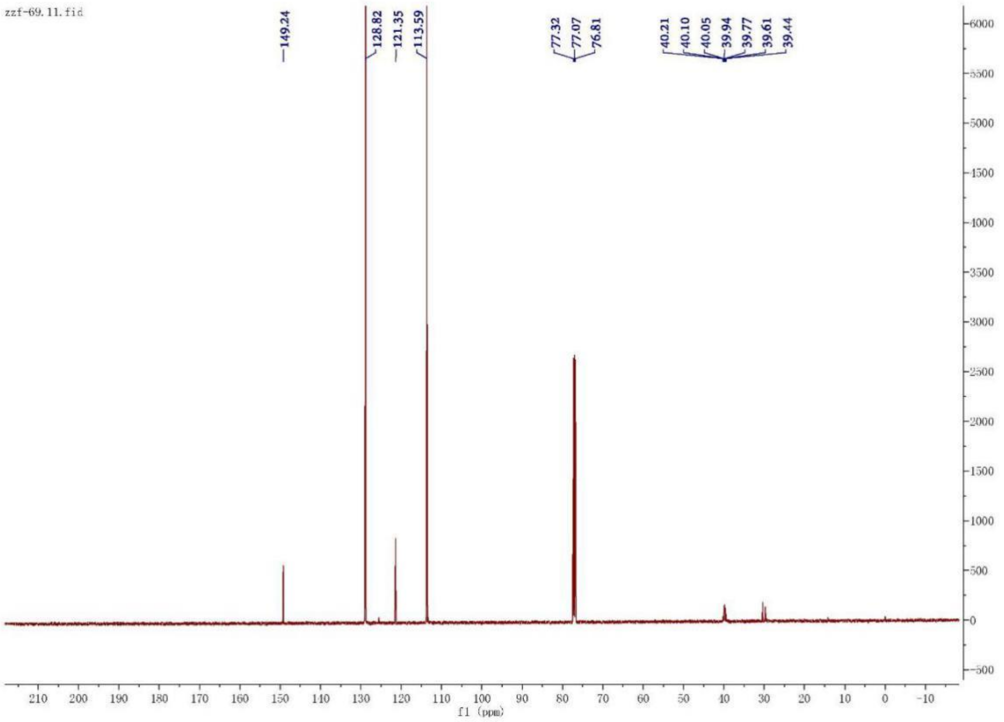

Supplementary Figure 32. ^{13}C NMR of product **3ad**.

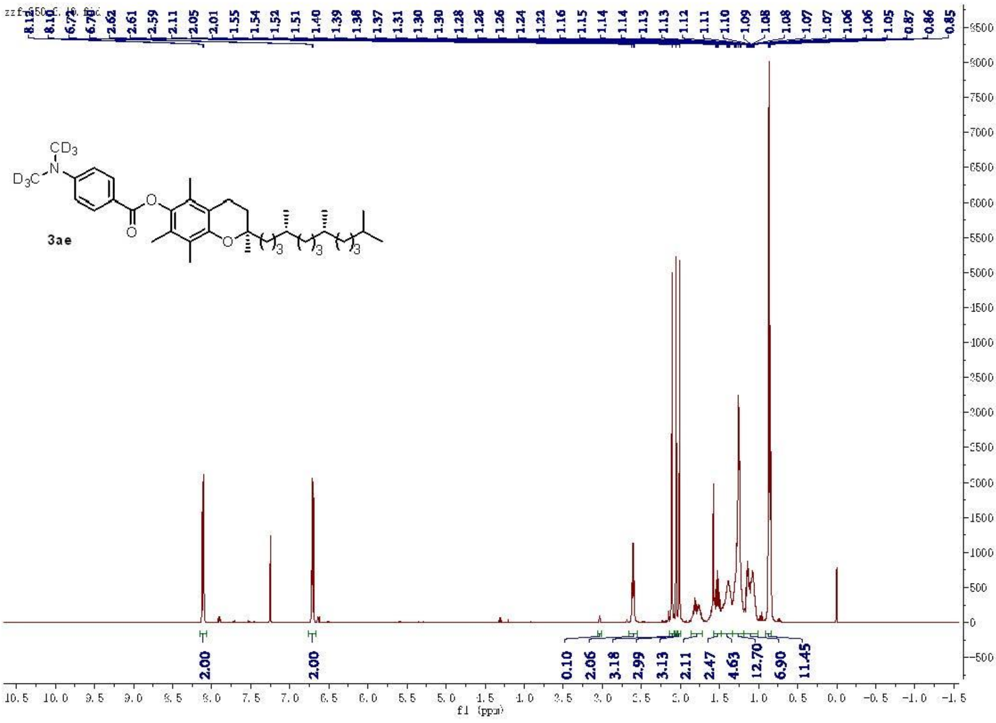

Supplementary Figure 33. ^{1}H NMR of product **3ae**.

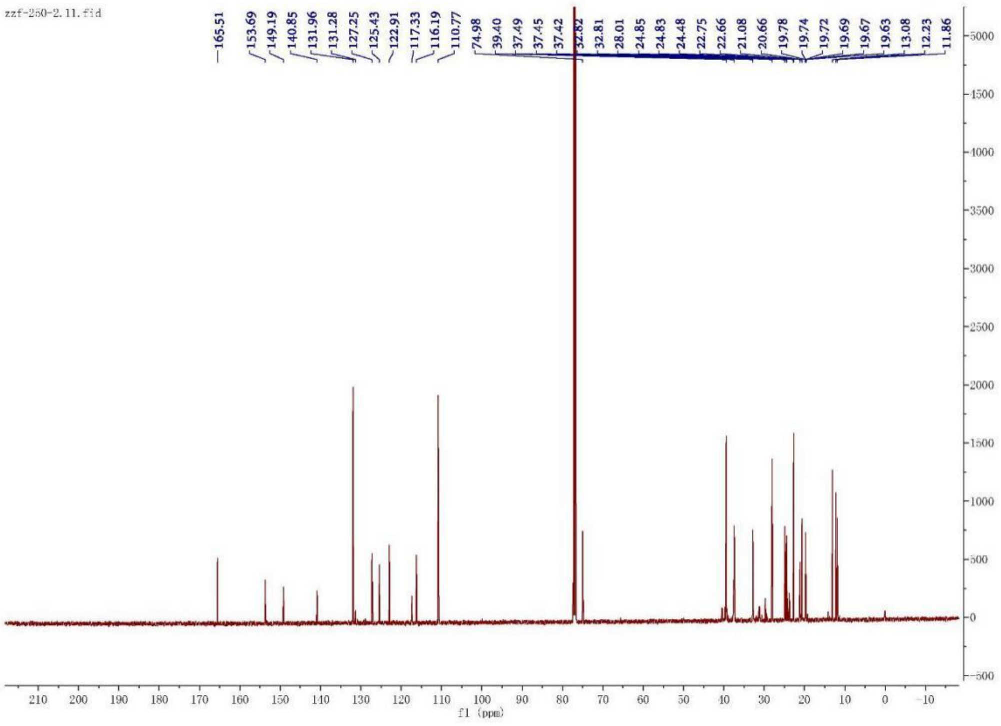

Supplementary Figure 34. ^{13}C NMR of product **3ae**.

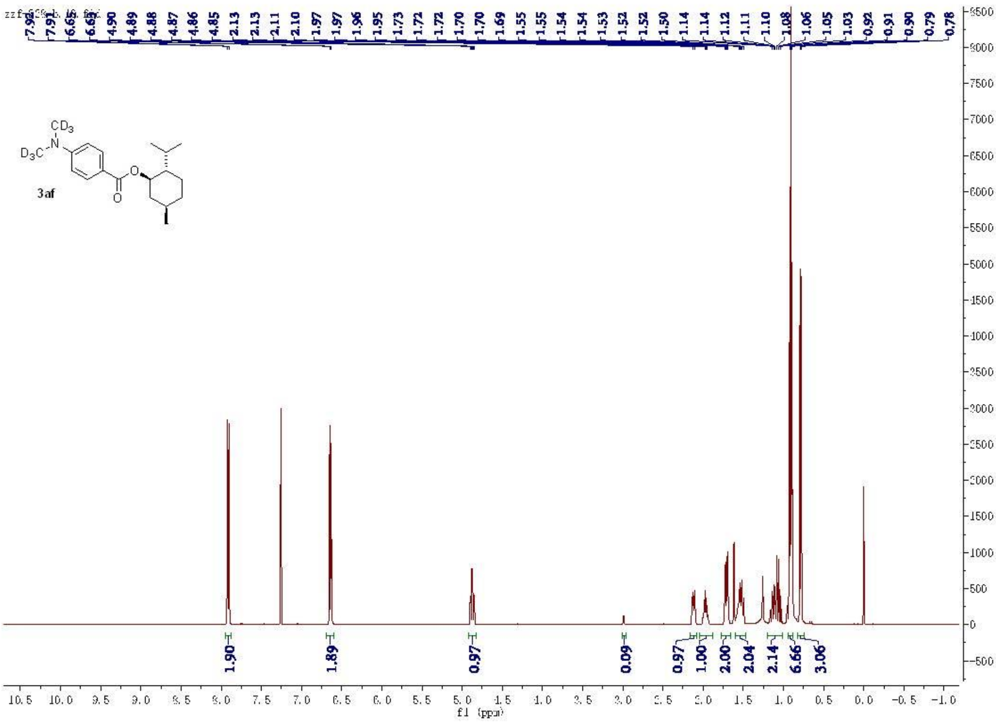

Supplementary Figure 35. ^{1}H NMR of product **3af**.

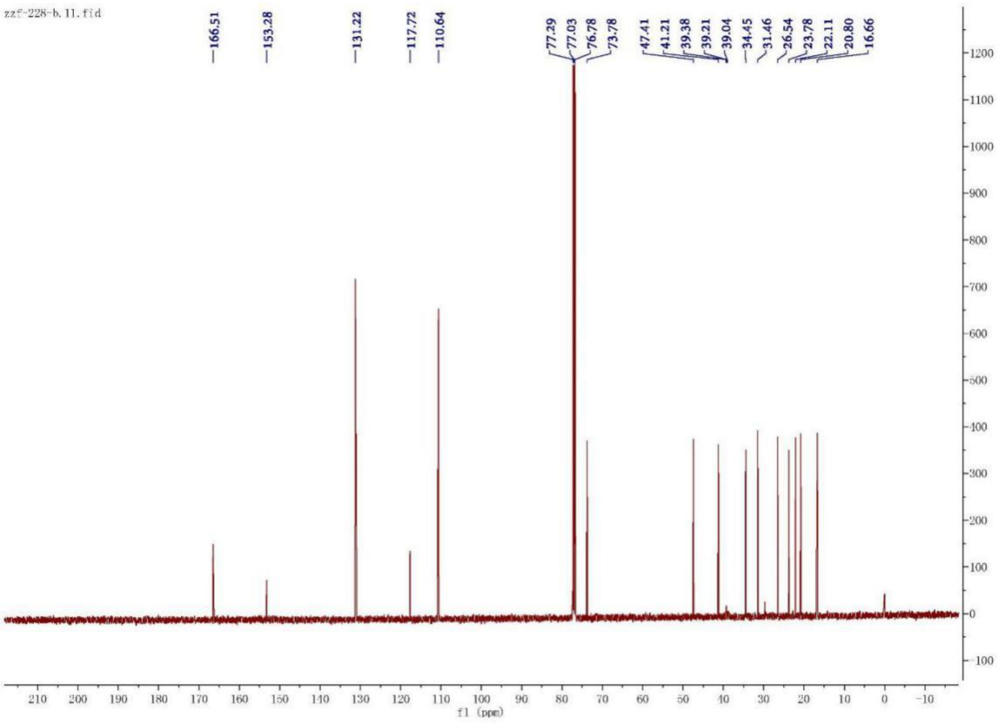

Supplementary Figure 36. ^{13}C NMR of product **3af**.

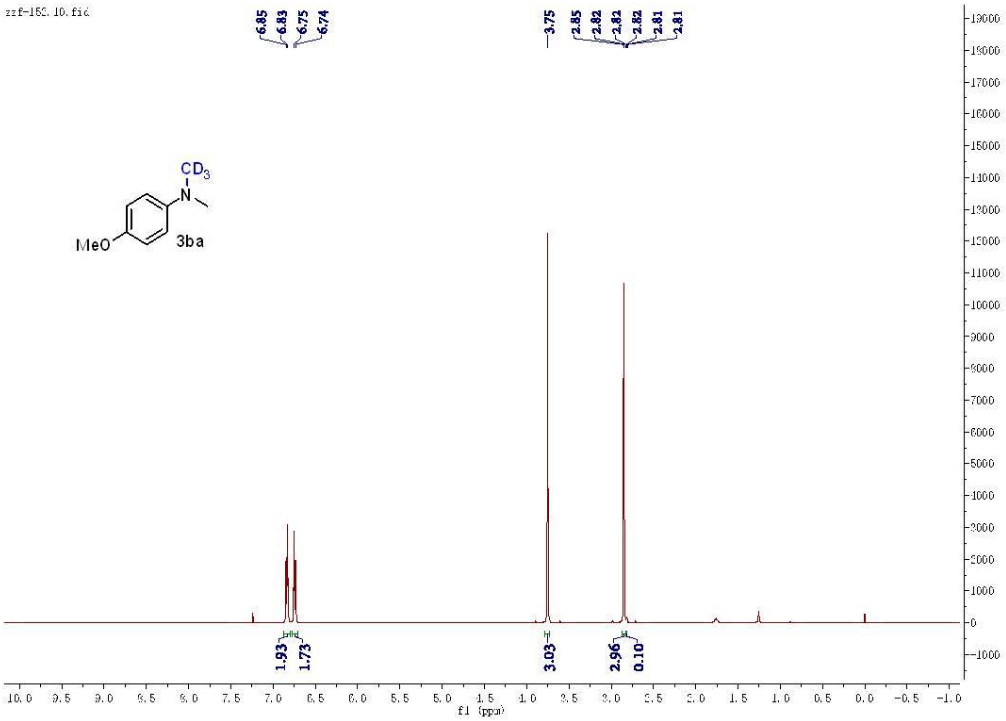

Supplementary Figure 37. ^{1}H NMR of product **3ba**.

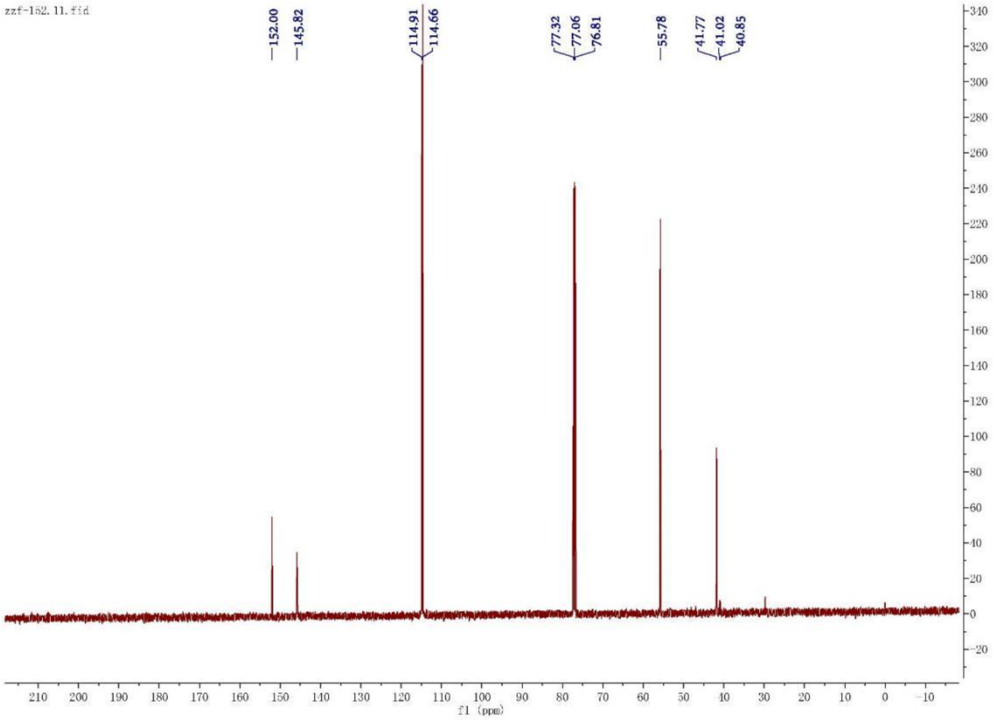

Supplementary Figure 38. ^{13}C NMR of product **3ba**.

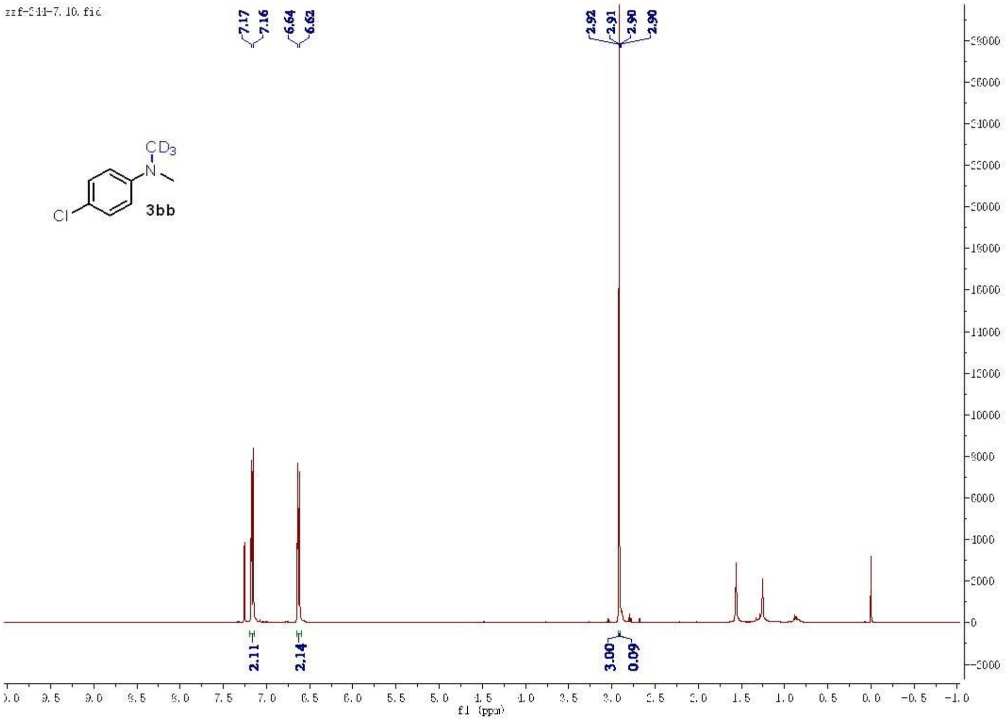

Supplementary Figure 39. ^{1}H NMR of product **3bb**.

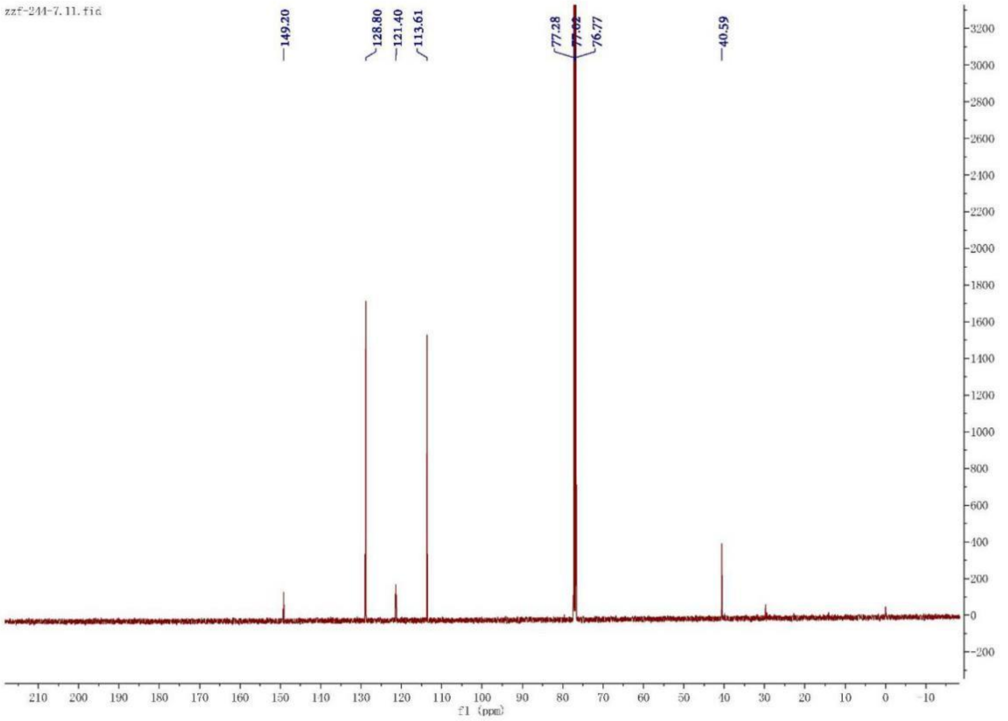

Supplementary Figure 40. ^{13}C NMR of product **3bb**.

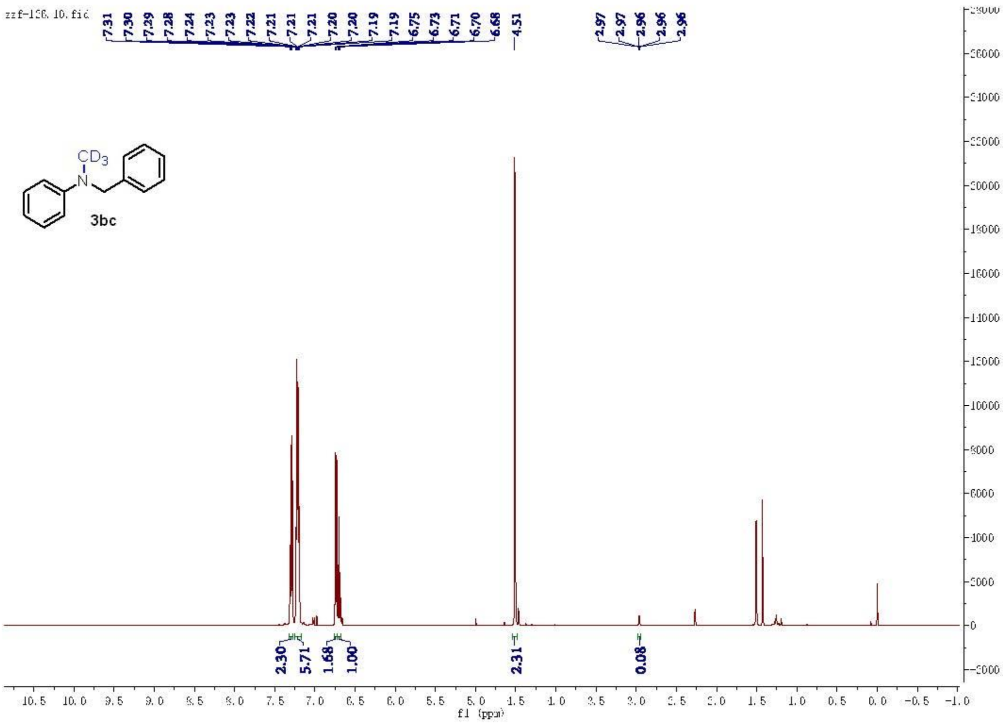

Supplementary Figure 41. ^{1}H NMR of product **3bc**.

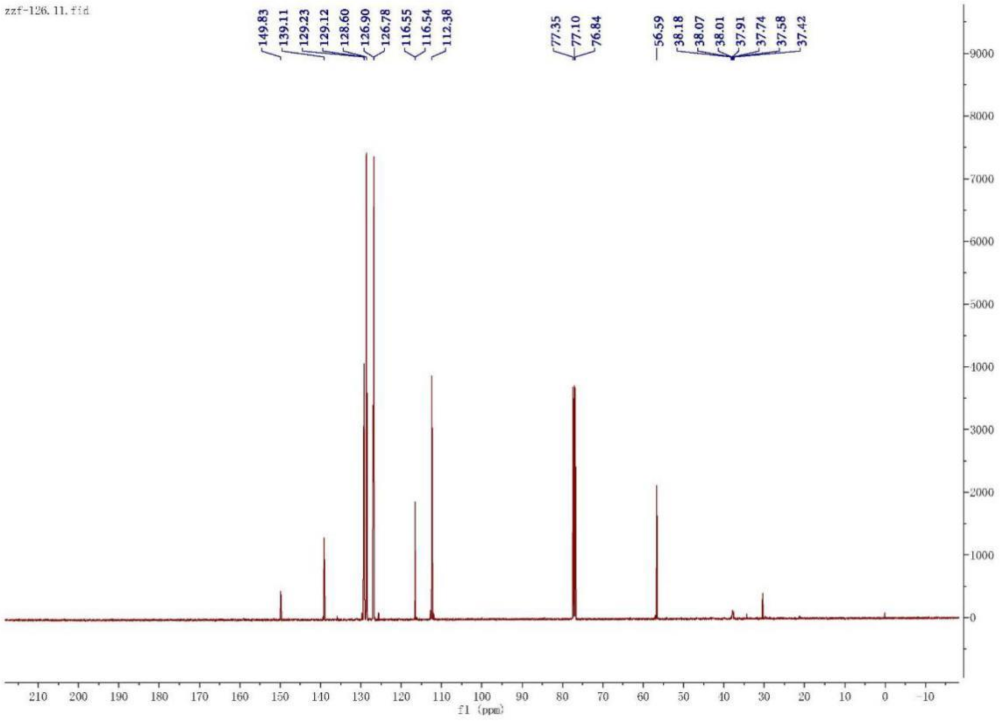

Supplementary Figure 42. ^{13}C NMR of product **3bc**.

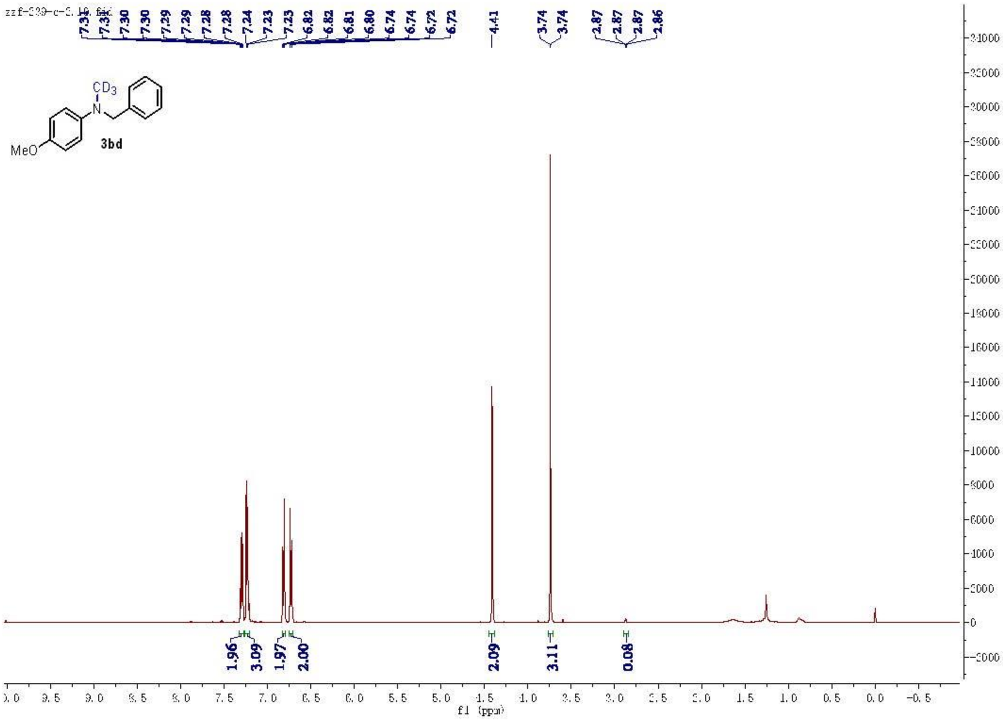

Supplementary Figure 43. ^{1}H NMR of product **3bd**.

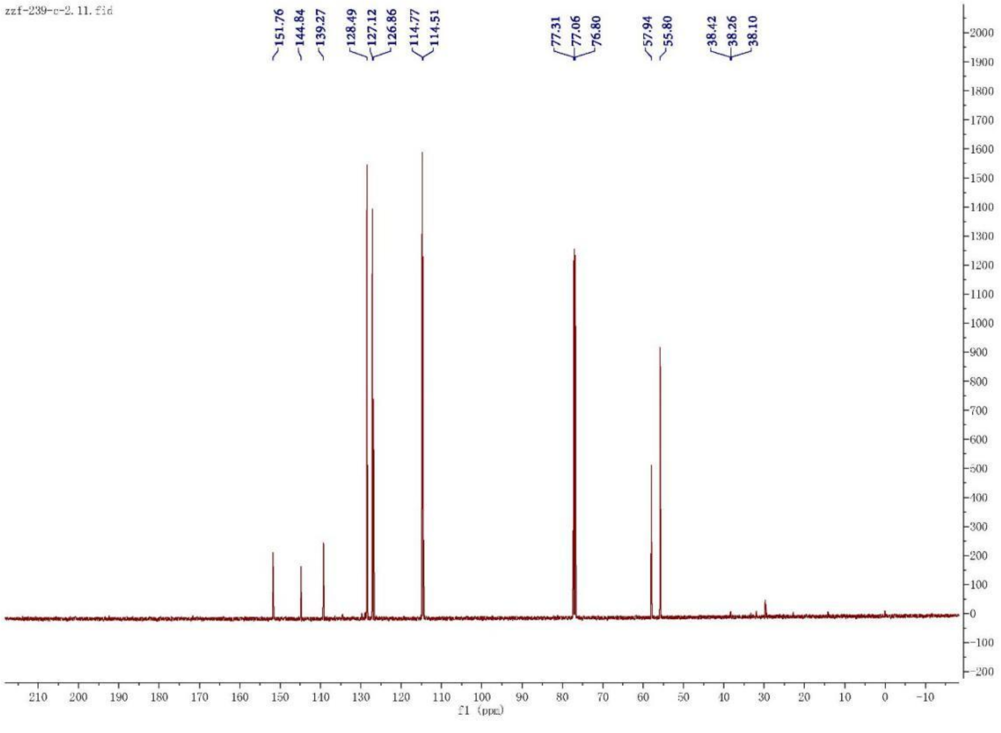

Supplementary Figure 44. ^{13}C NMR of product **3bd**.

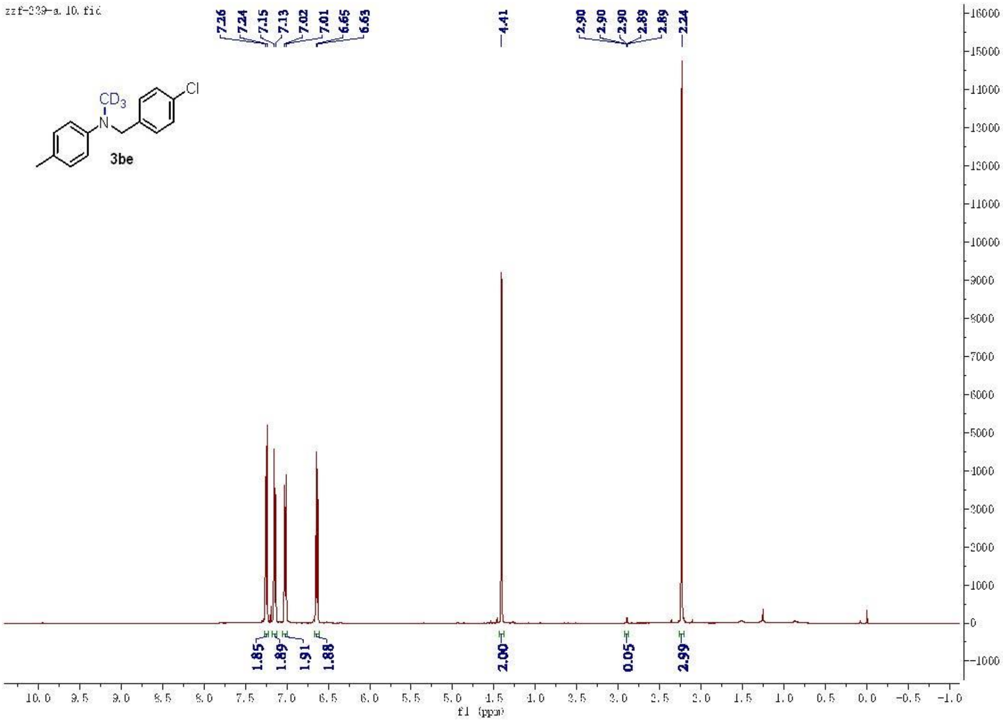

Supplementary Figure 45. ^{1}H NMR of product **3be**.

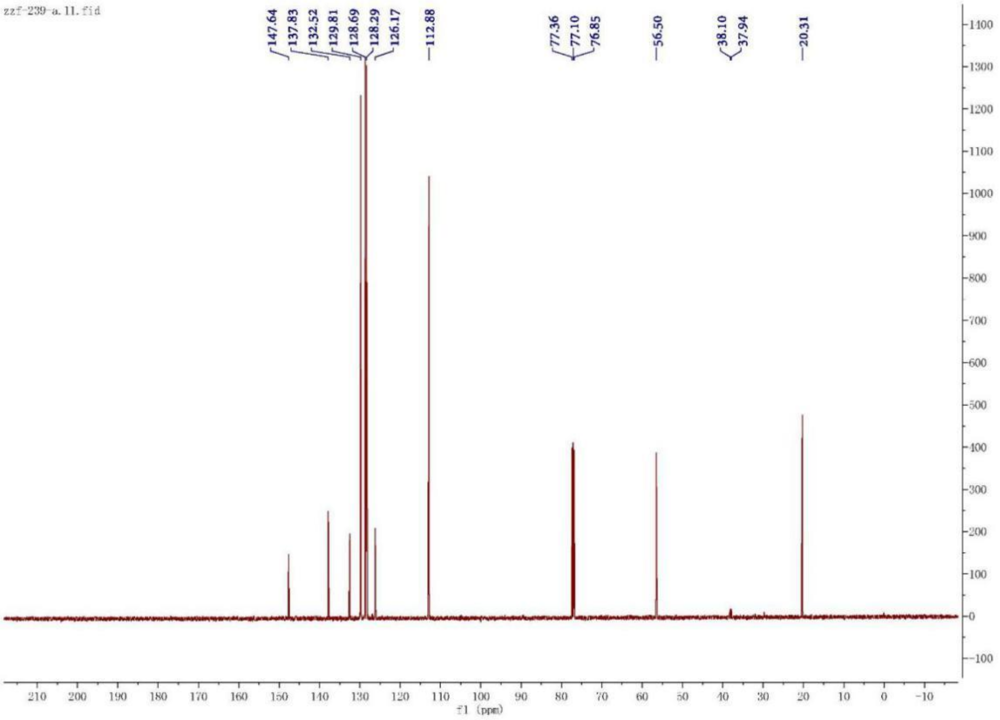

Supplementary Figure 46. ^{13}C NMR of product **3be**.

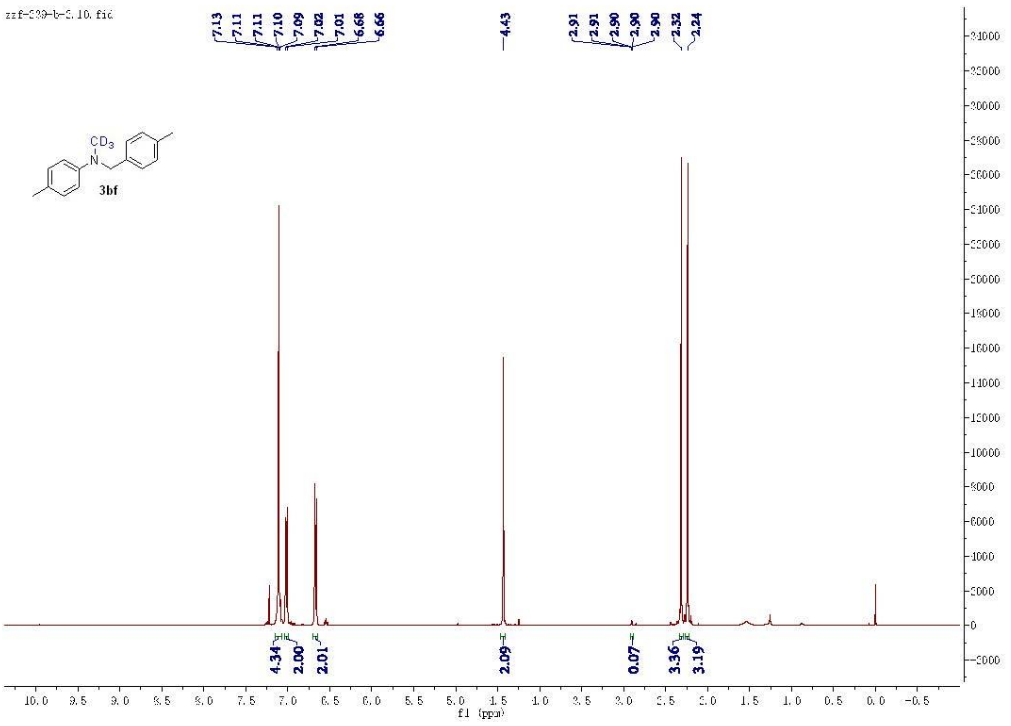

Supplementary Figure 47. ^{1}H NMR of product **3bf**.

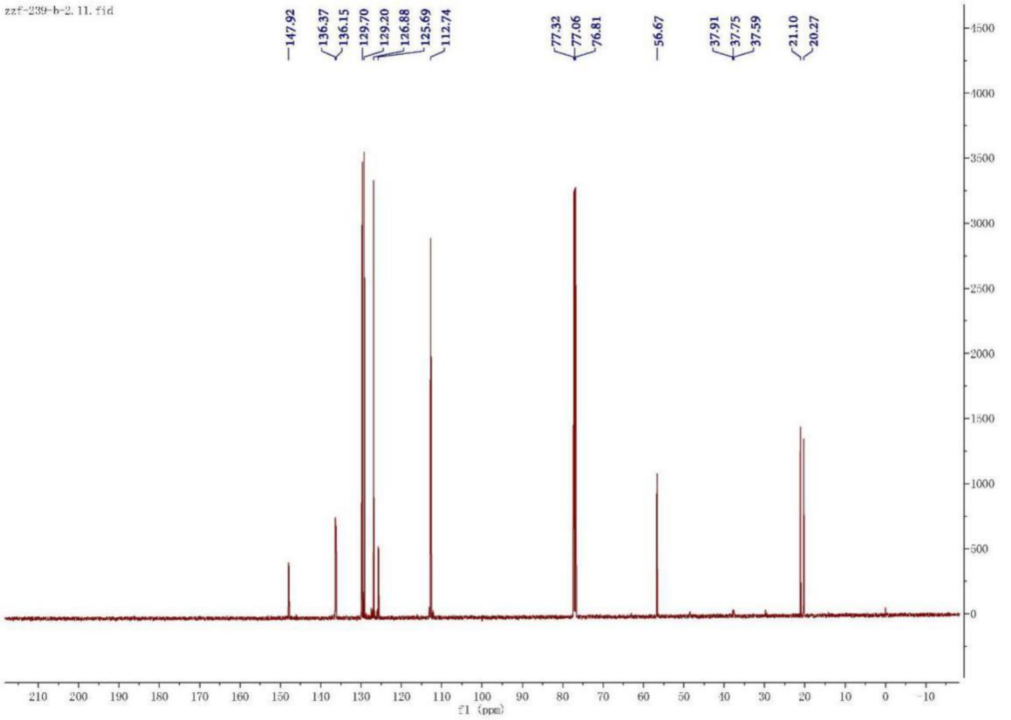

Supplementary Figure 48. ^{13}C NMR of product **3bf**.

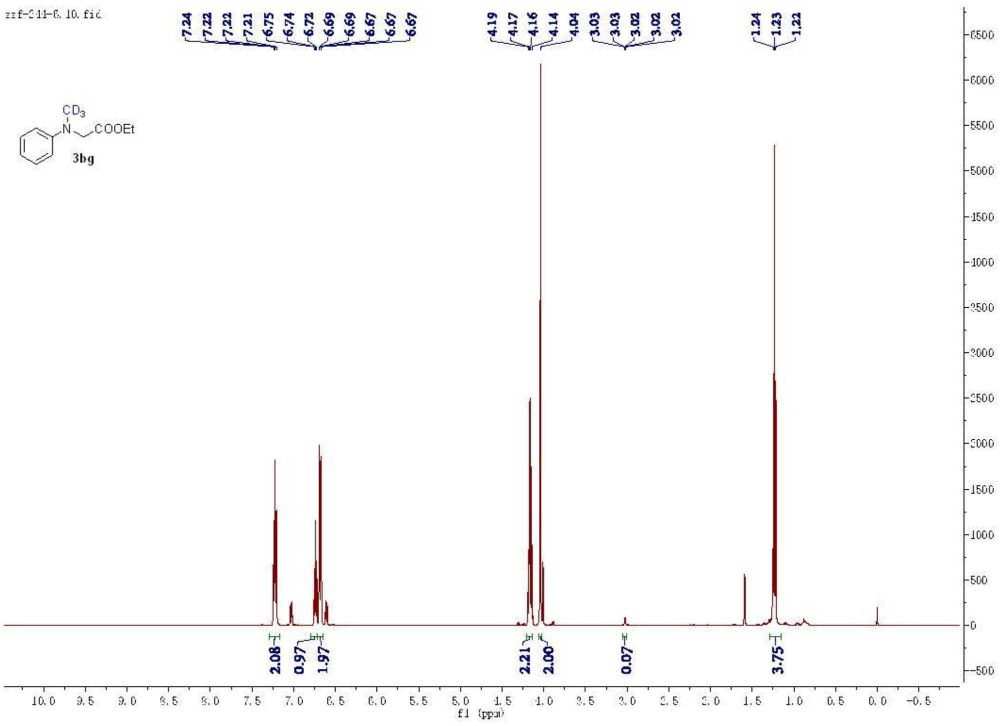

Supplementary Figure 49. ^{1}H NMR of product **3bg**.

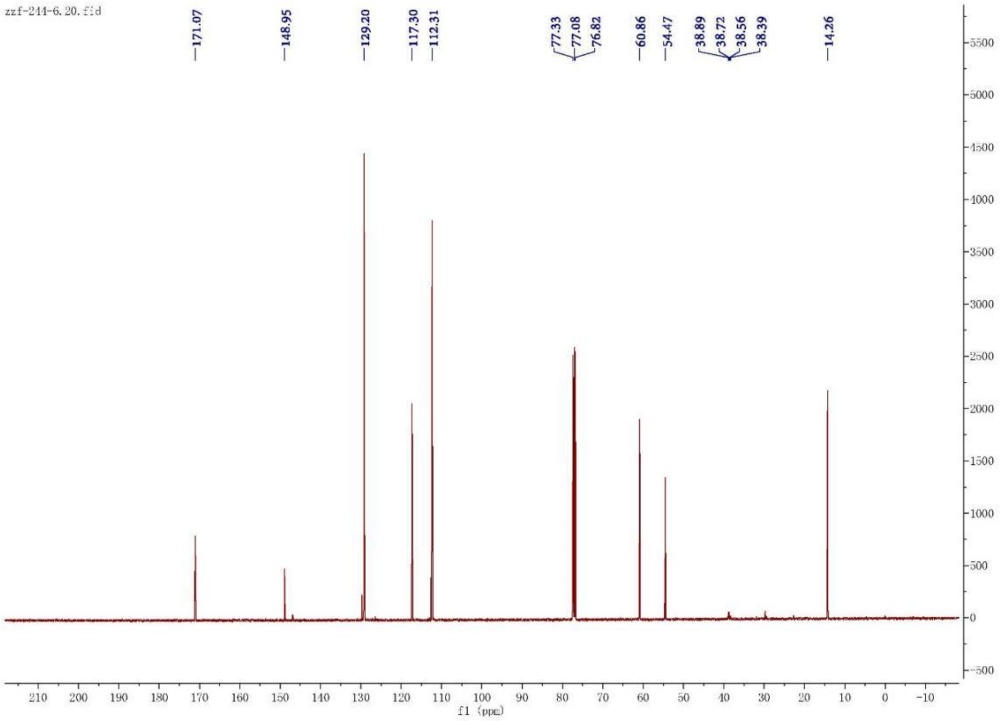

Supplementary Figure 50. ^{13}C NMR of product **3bg**.

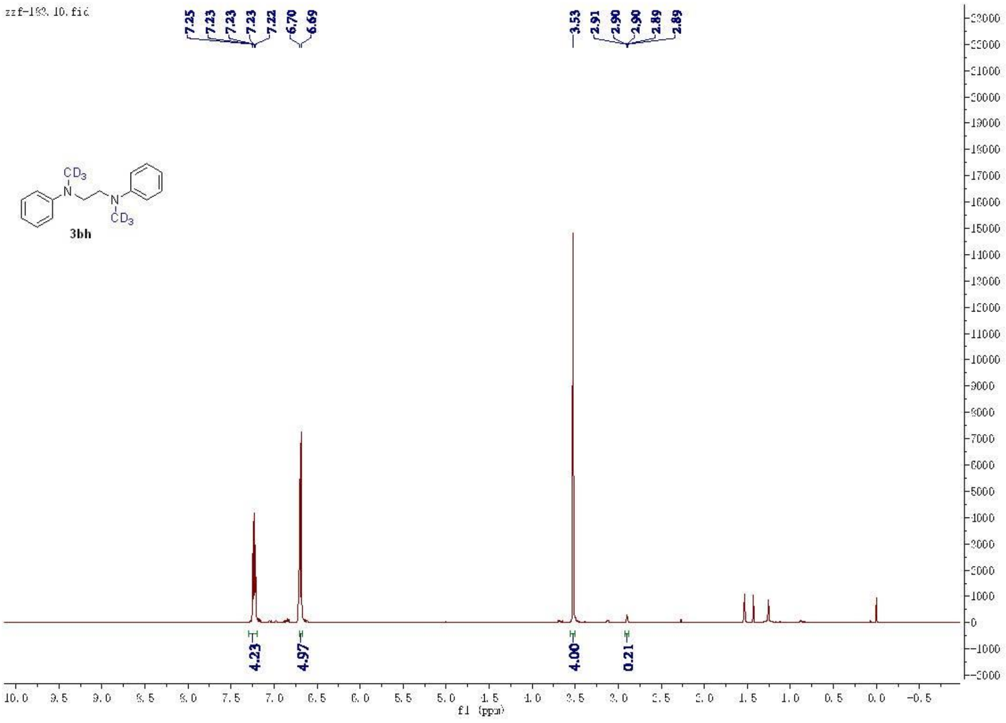

Supplementary Figure 51. ^{1}H NMR of product **3bh**.

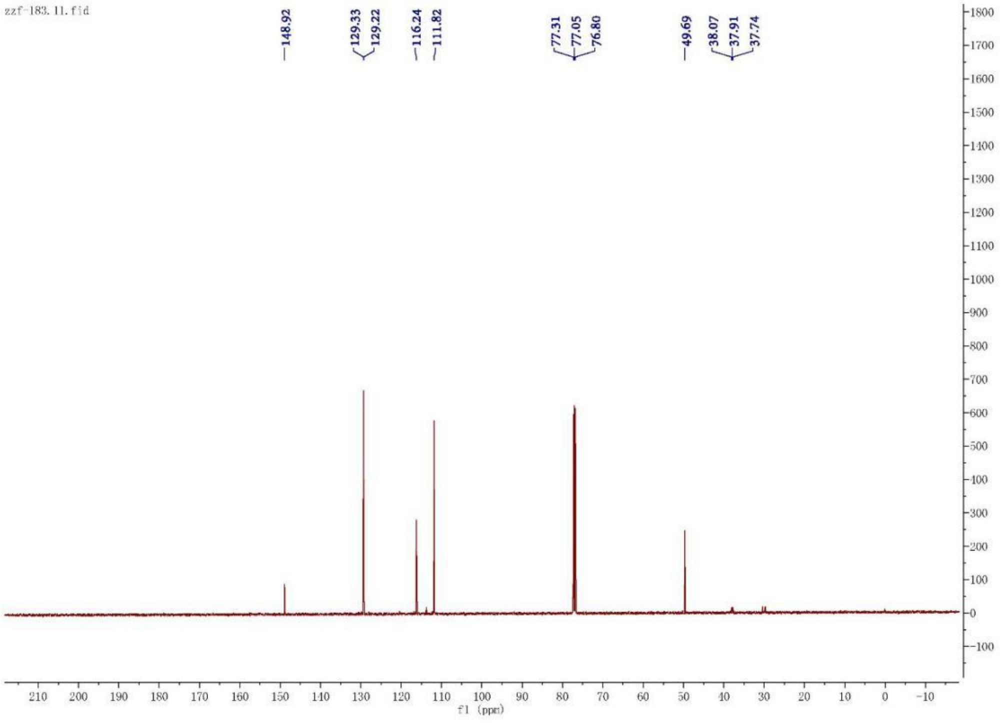

Supplementary Figure 52. ^{13}C NMR of product **3bh**.

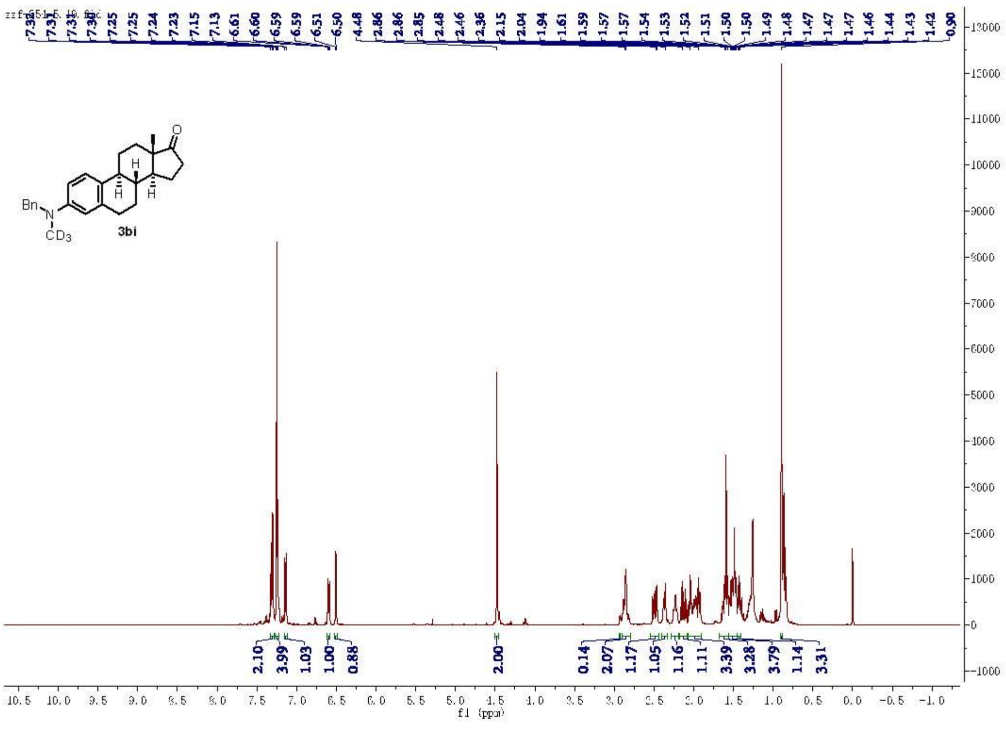

Supplementary Figure 53. ^{1}H NMR of product **3bi**.

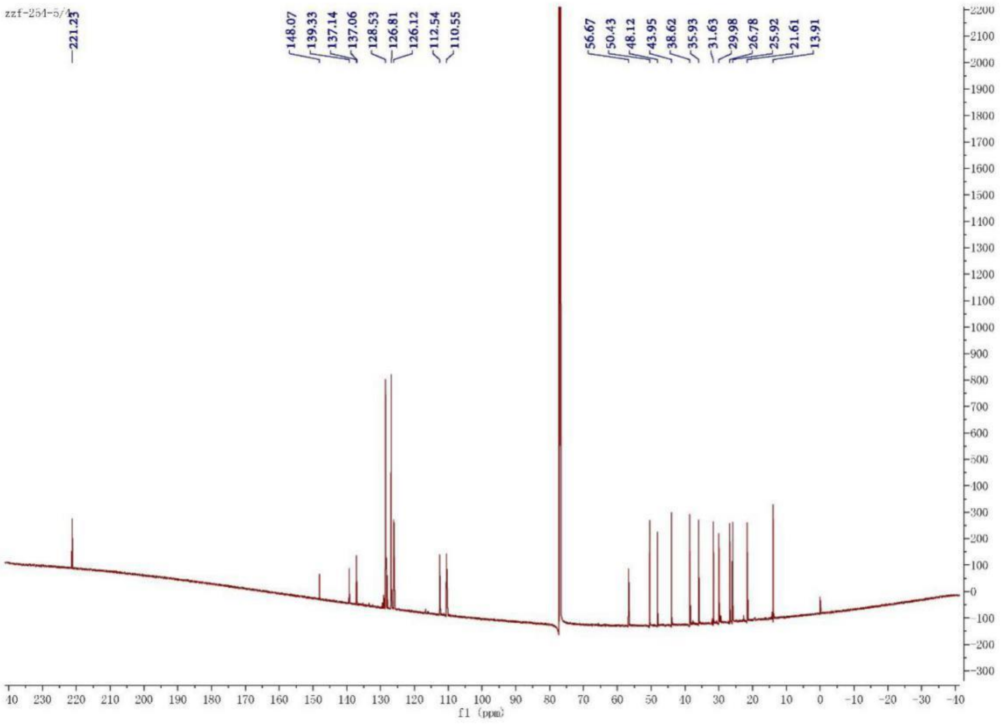

Supplementary Figure 54. ^{13}C NMR of product **3bi**.

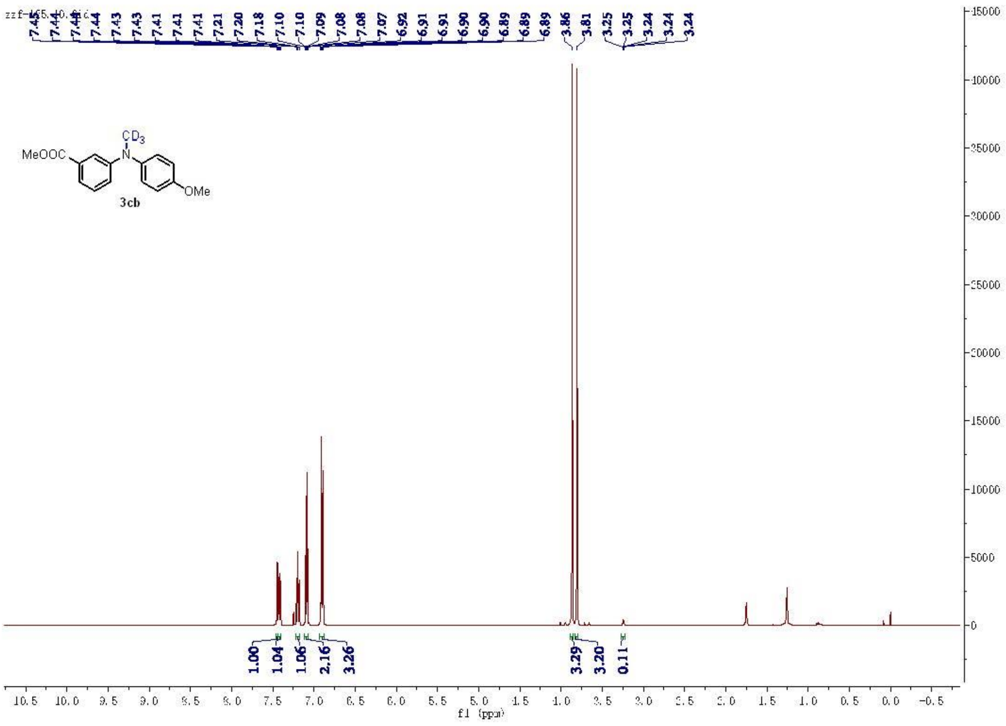

Supplementary Figure 55. ^{1}H NMR of product **3cb**.

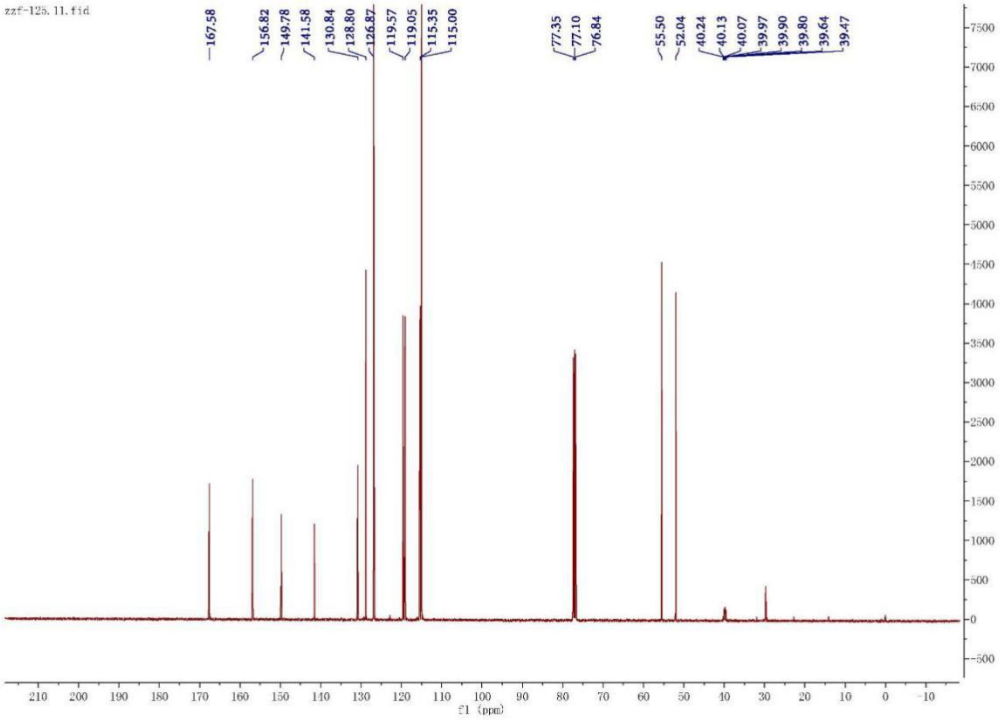

Supplementary Figure 56. ^{13}C NMR of product **3cb**.

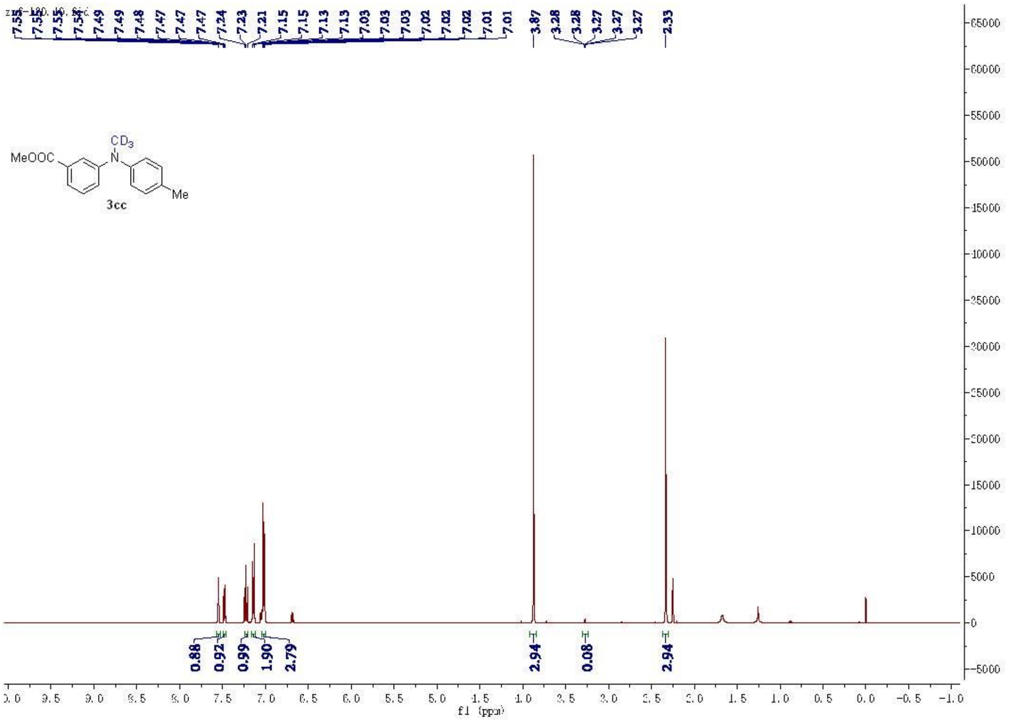

Supplementary Figure 57. ^{1}H NMR of product **3cc**.

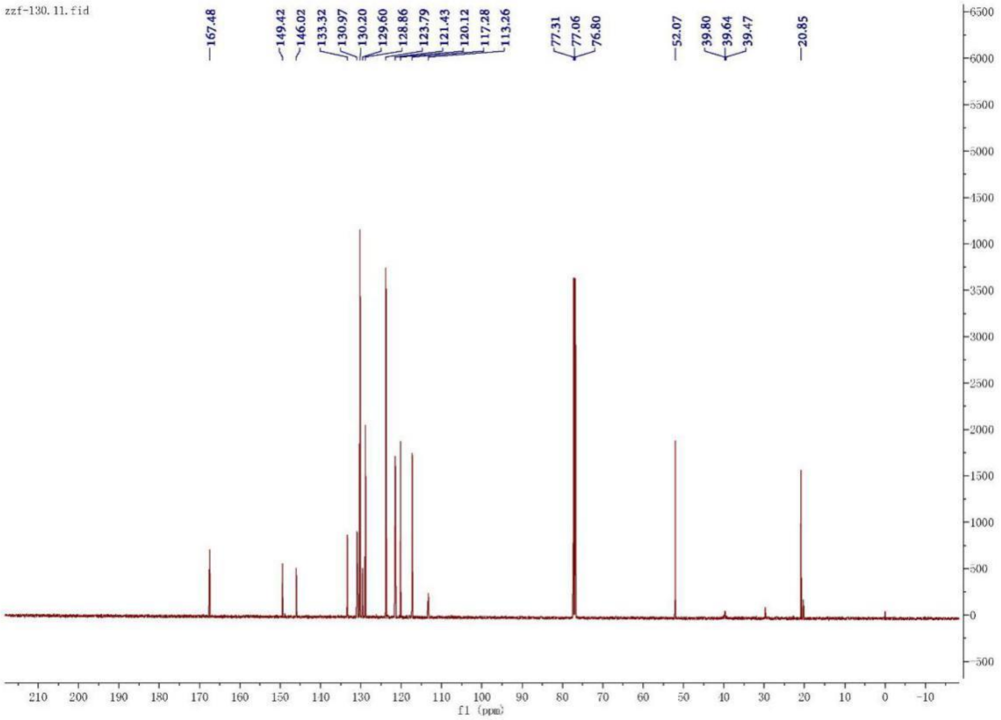

Supplementary Figure 58. ^{13}C NMR of product **3cc**.

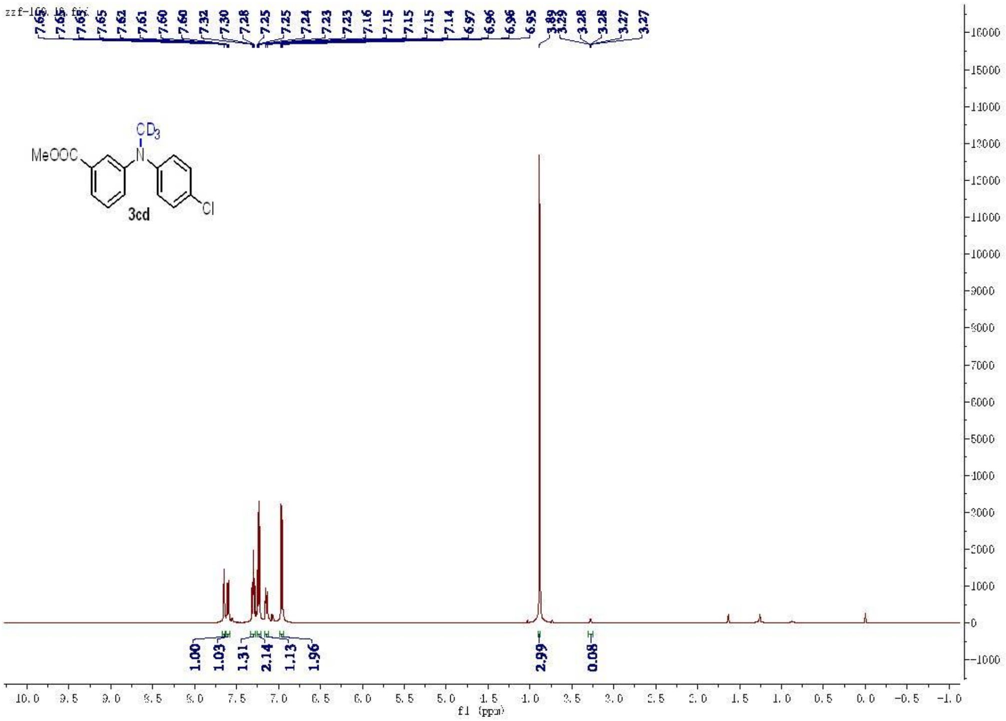

Supplementary Figure 59. ^{1}H NMR of product **3cd**.

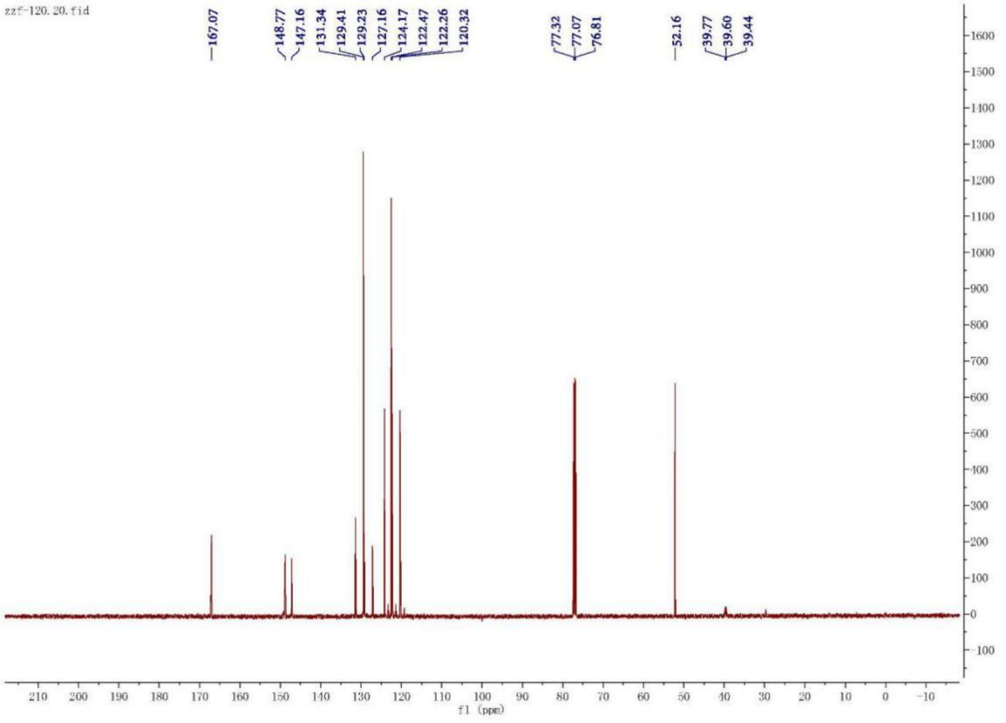

Supplementary Figure 60. ^{13}C NMR of product **3cd**.

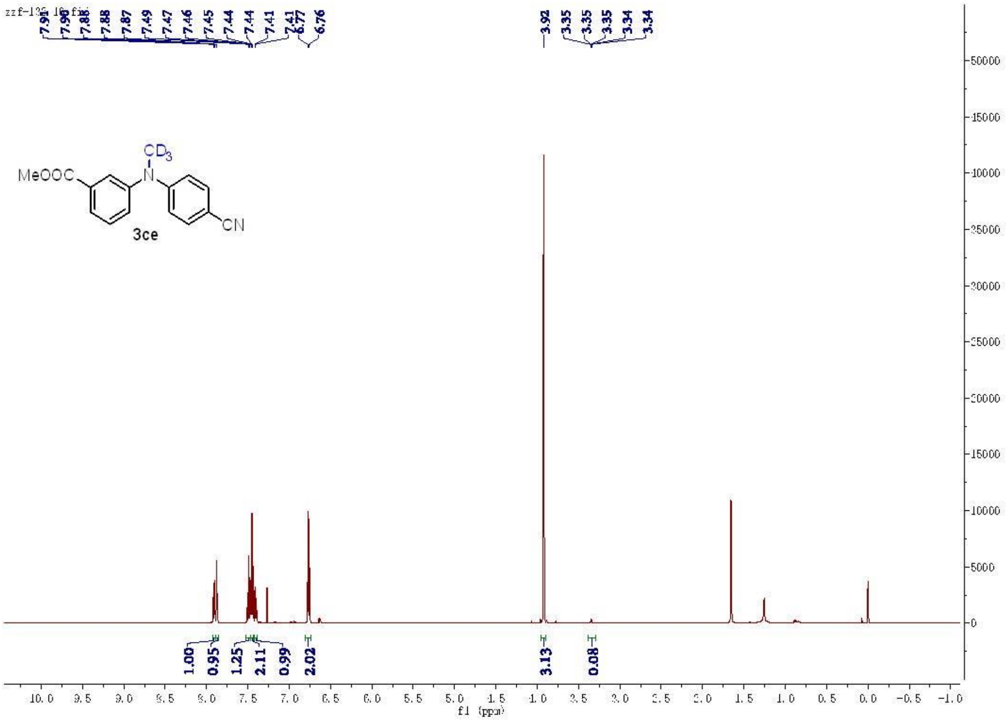

Supplementary Figure 61. ^{1}H NMR of product **3ce**.

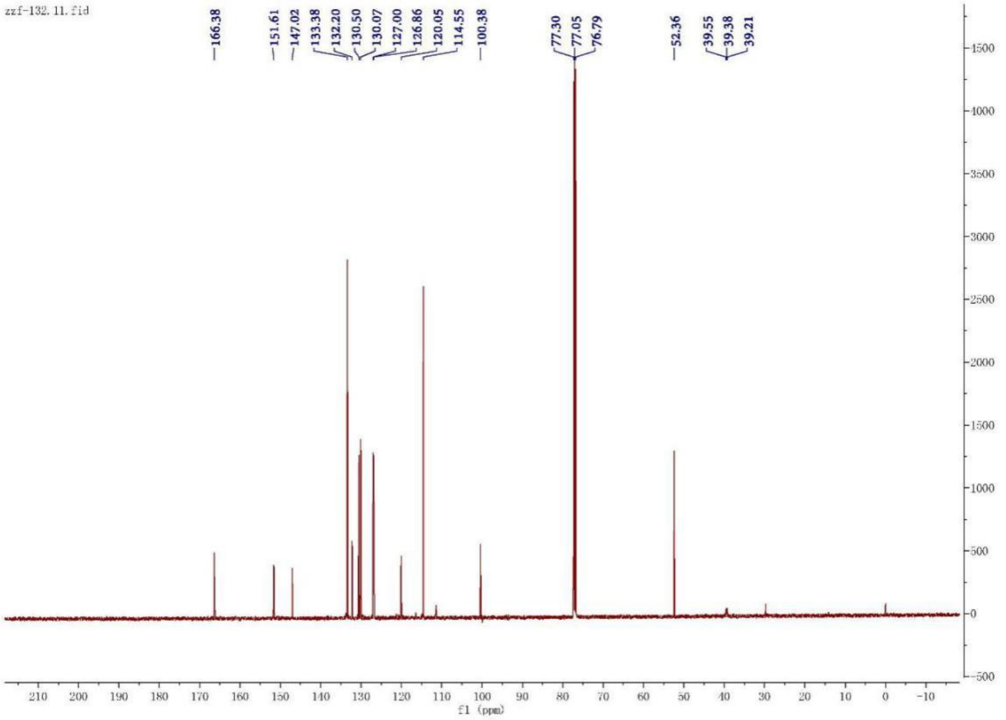

Supplementary Figure 62. ^{13}C NMR of product **3ce**.

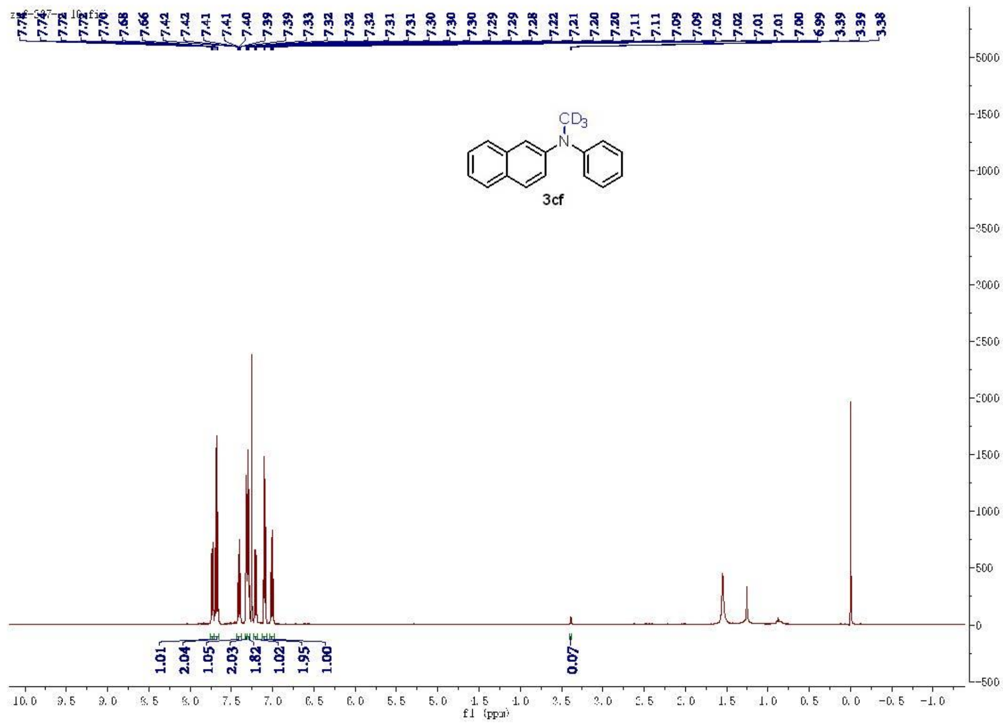

Supplementary Figure 63. ^{1}H NMR of product **3cf**.

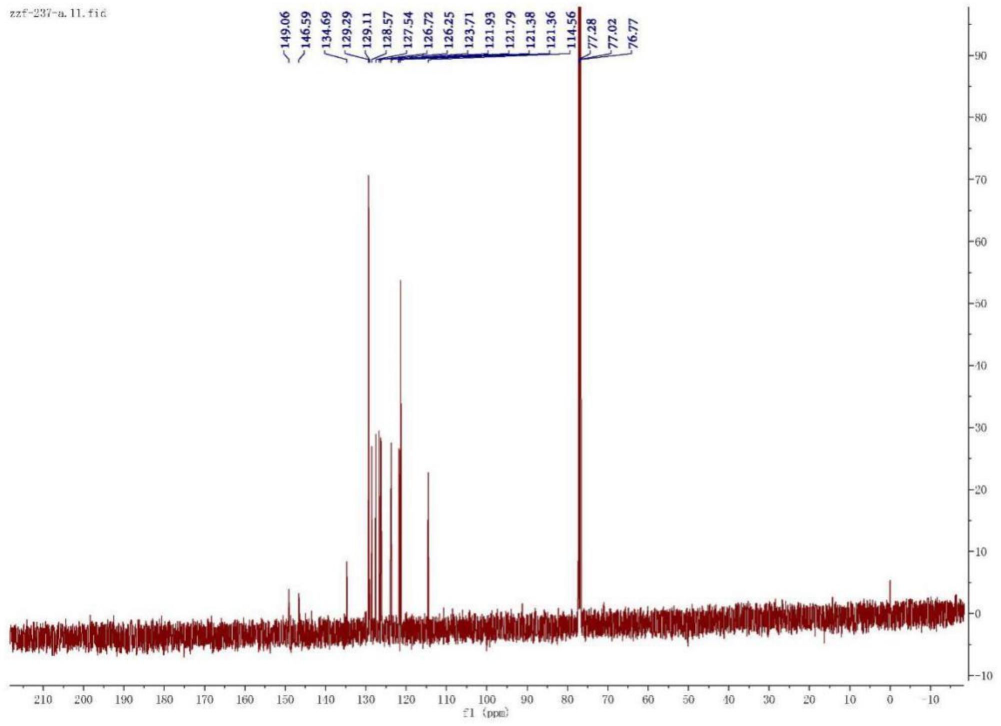

Supplementary Figure 64. ^{13}C NMR of product **3cf**.

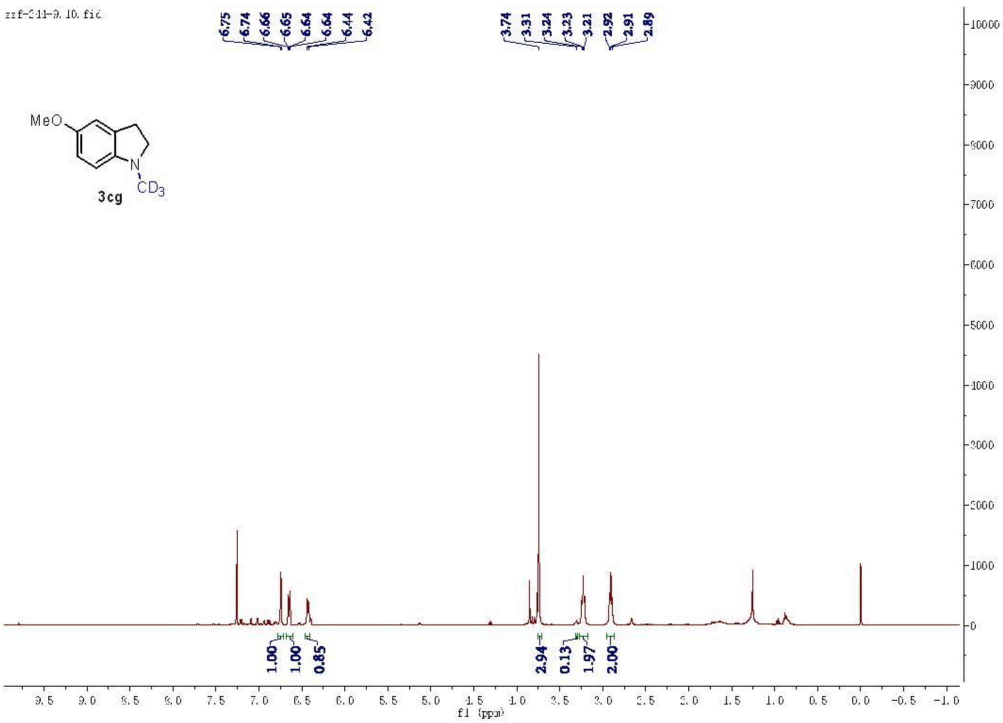

Supplementary Figure 65. ^{1}H NMR of product **3cg**.

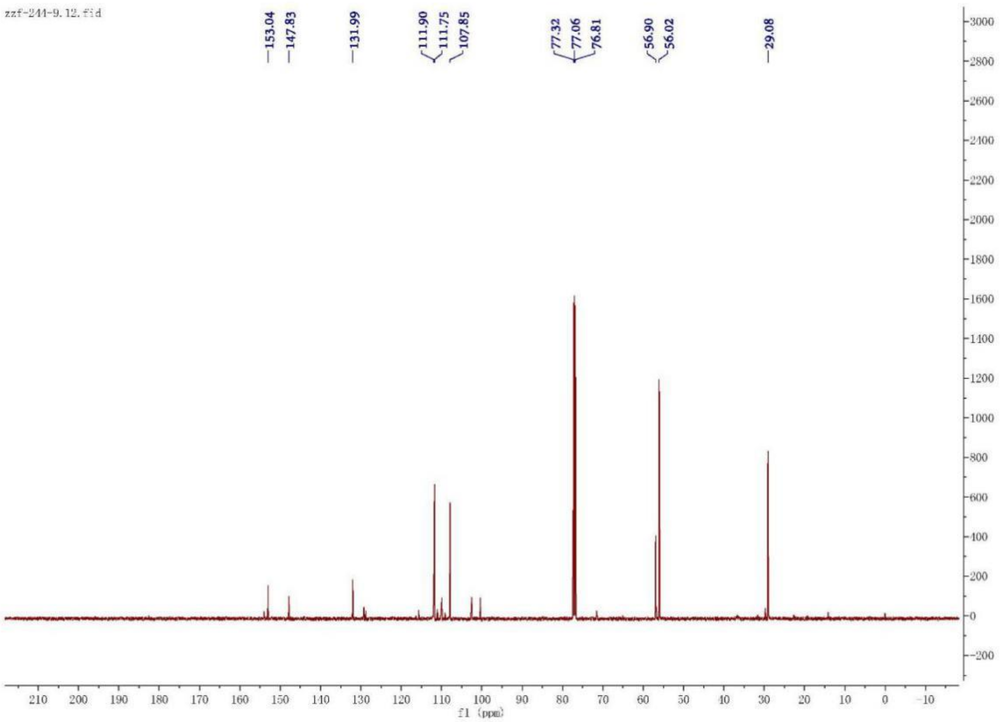

Supplementary Figure 66. ^{13}C NMR of product **3cg**.

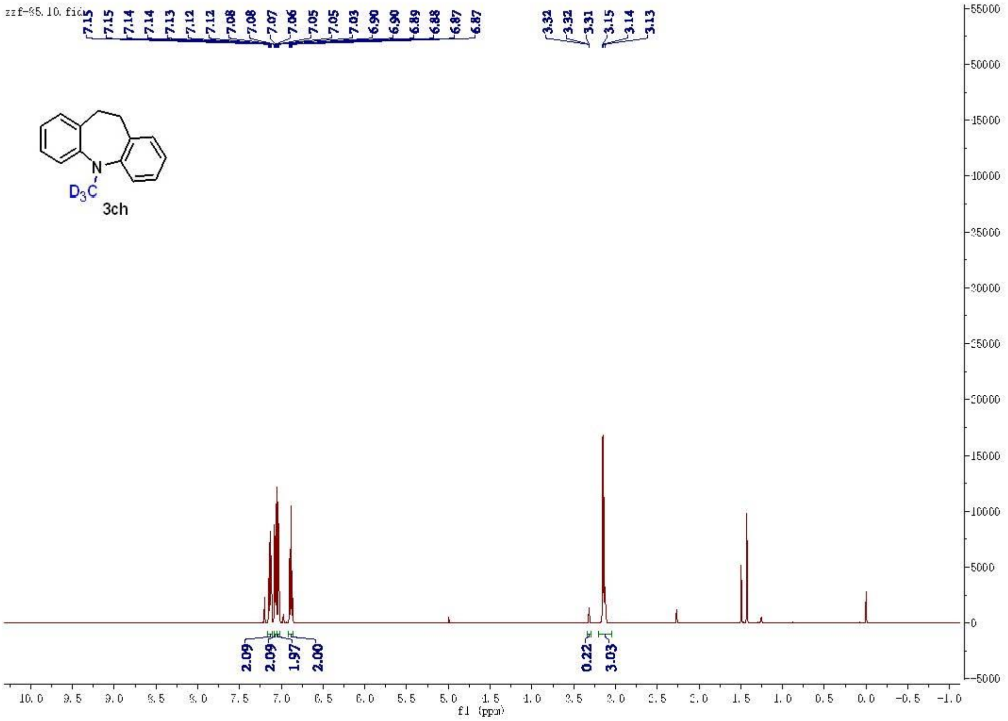

Supplementary Figure 67. ^{1}H NMR of product **3ch**.

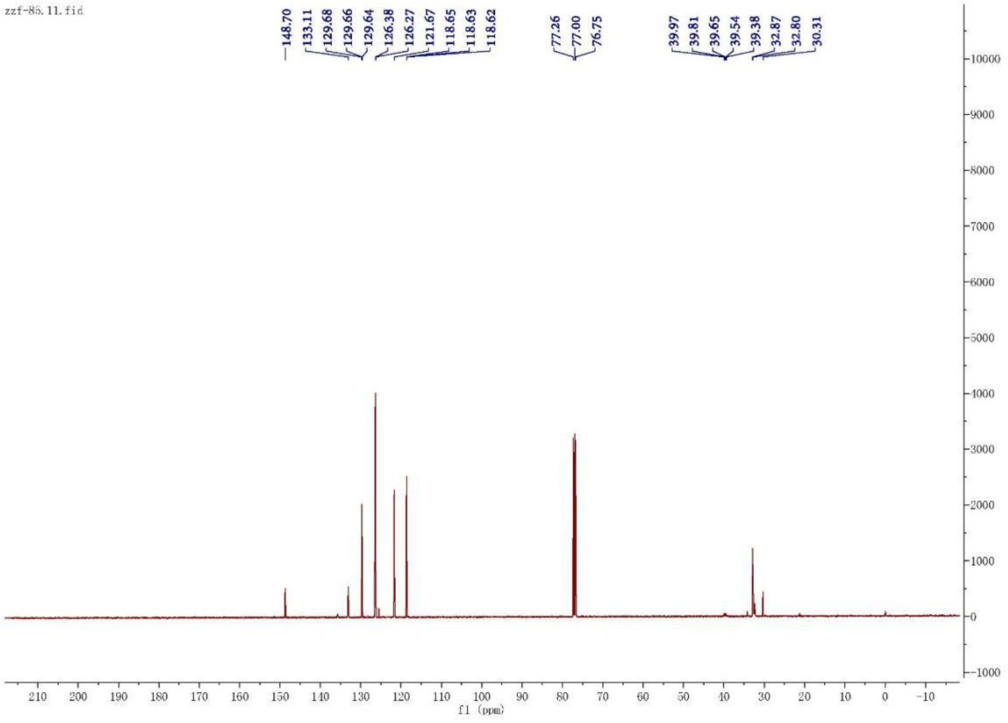

Supplementary Figure 68. ^{13}C NMR of product **3ch**.

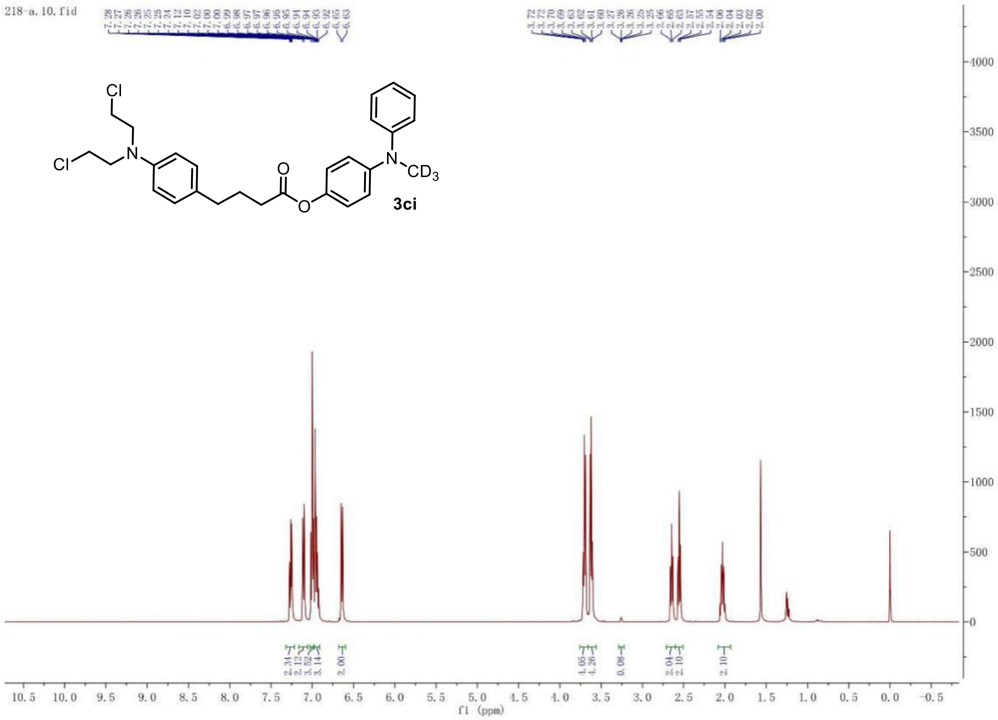

Supplementary Figure 69. ^{1}H NMR of product **3ci**.

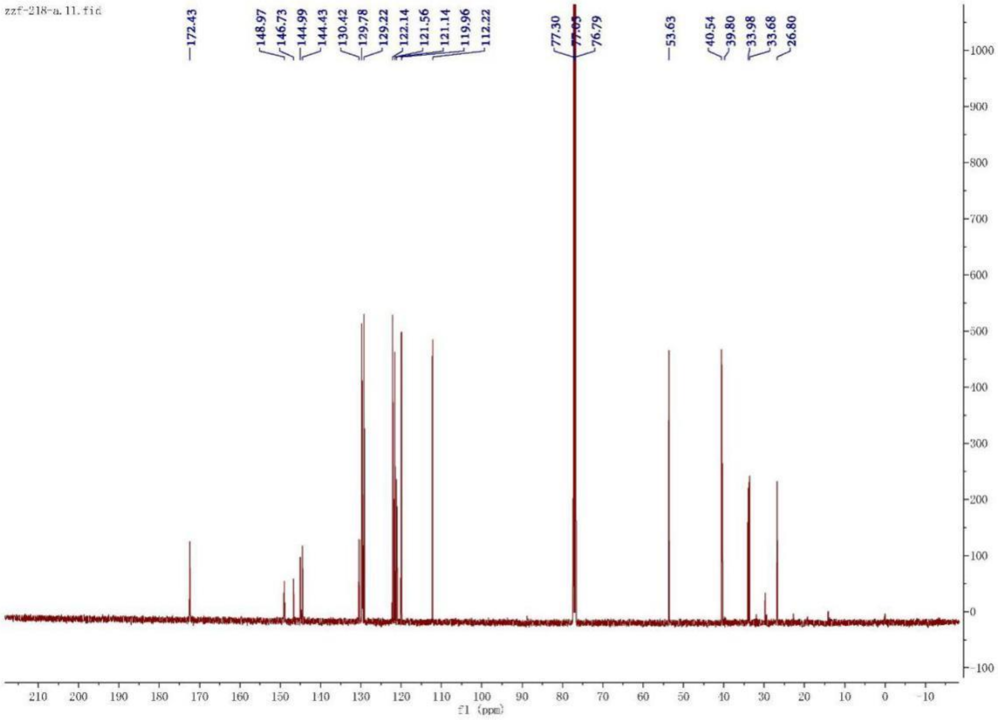

Supplementary Figure 70. ^{13}C NMR of product **3ci**.

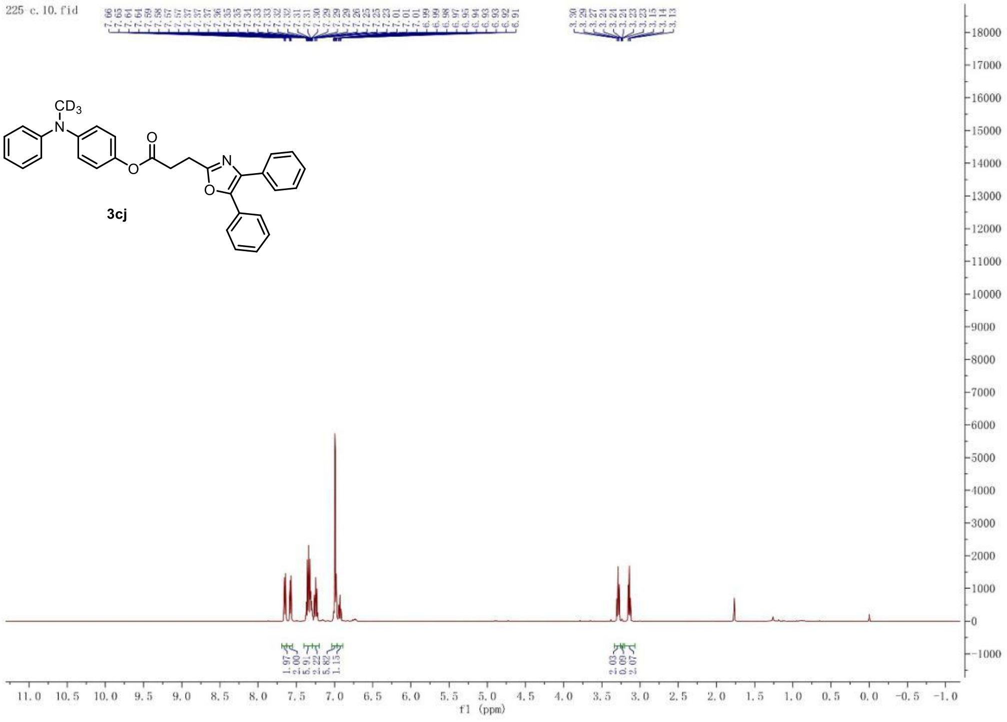

Supplementary Figure 71. ^{1}H NMR of product **3cj**.

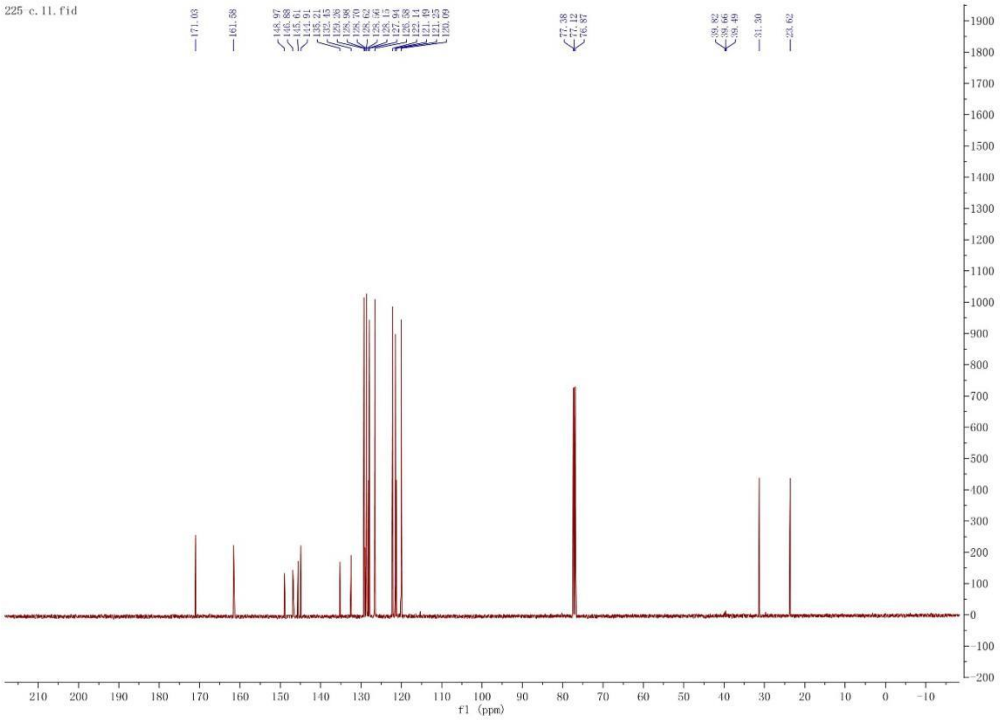

Supplementary Figure 72. ^{13}C NMR of product **3cj**.

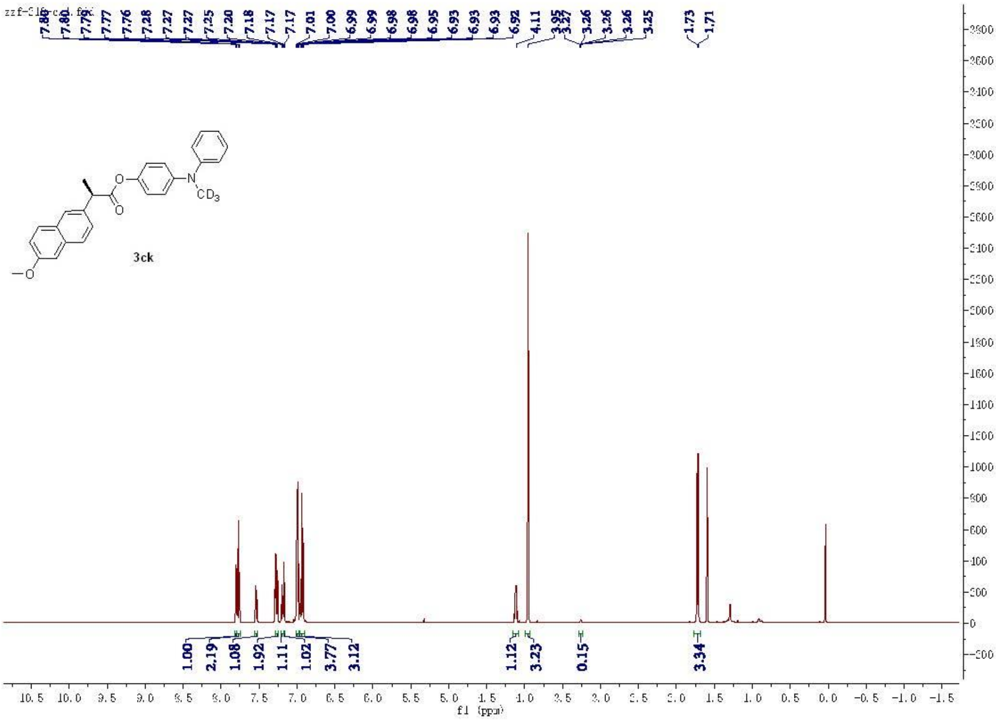

Supplementary Figure 73. ^{1}H NMR of product **3ck**.

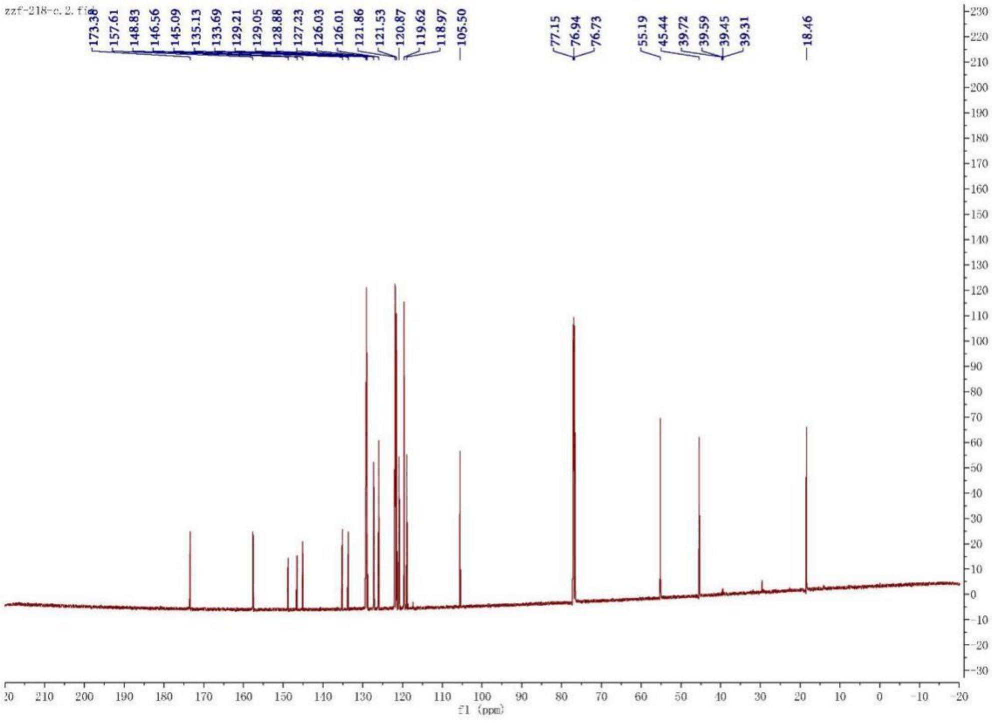

Supplementary Figure 74. ^{13}C NMR of product **3ck**.

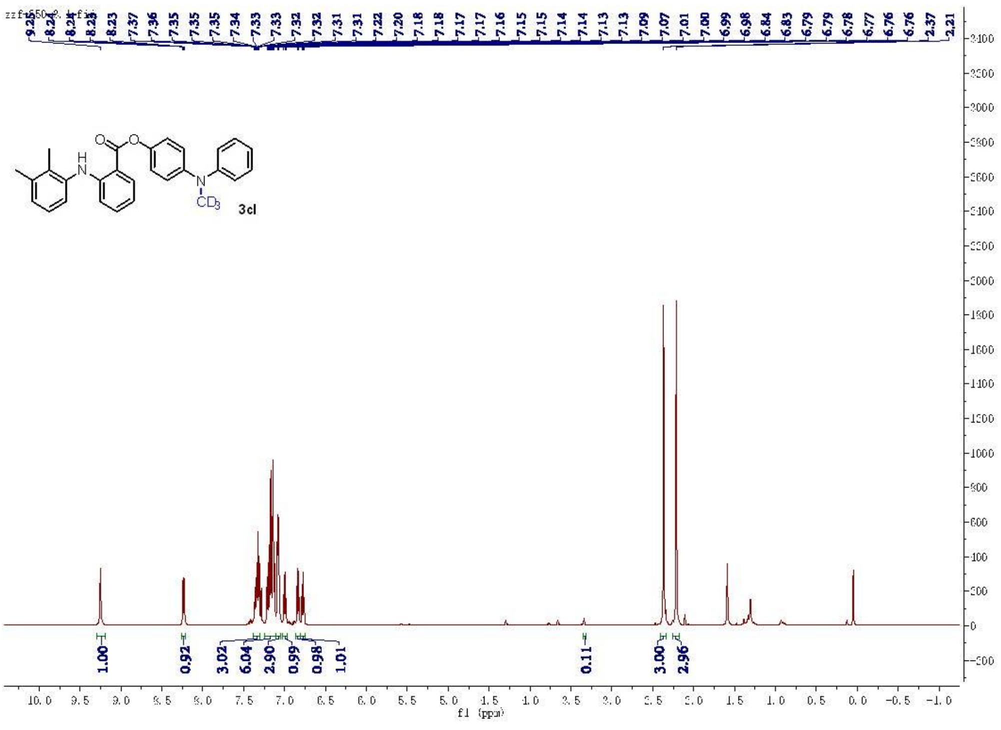

Supplementary Figure 75. ^{1}H NMR of product **3cl**.

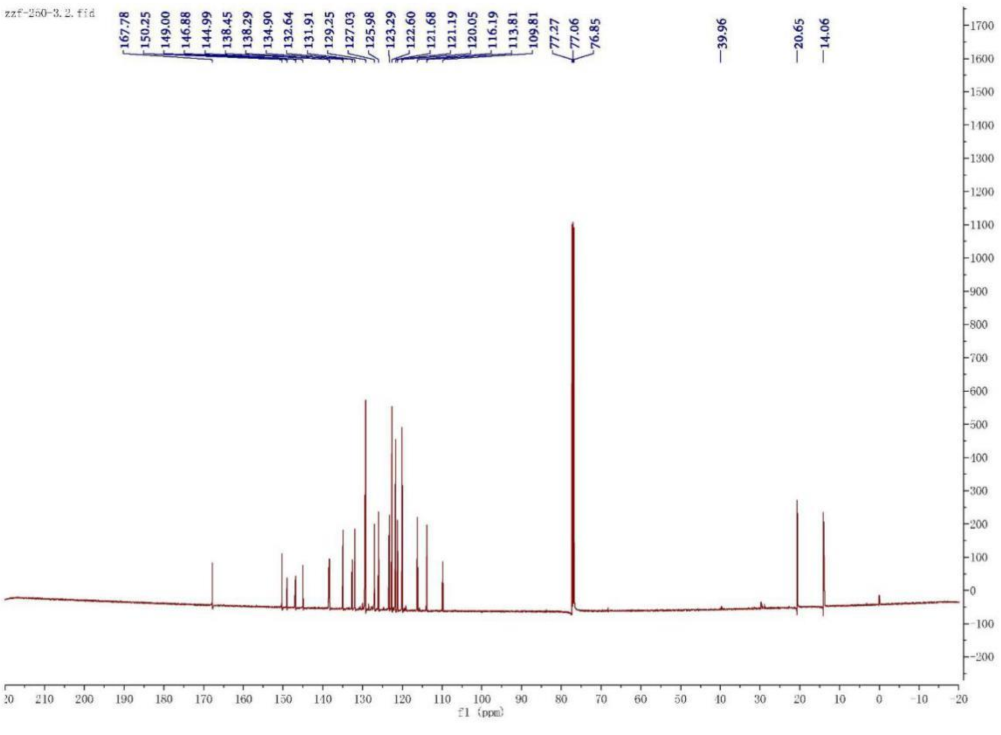

Supplementary Figure 76. ^{13}C NMR of product **3cl**.

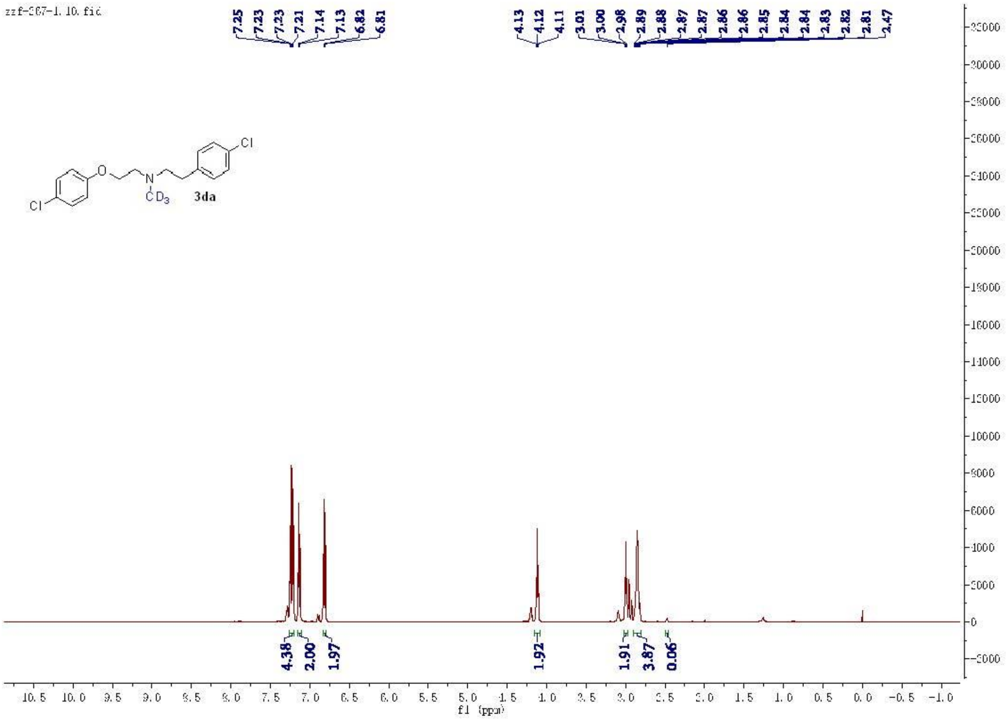

Supplementary Figure 77. ^{1}H NMR of product **3da**.

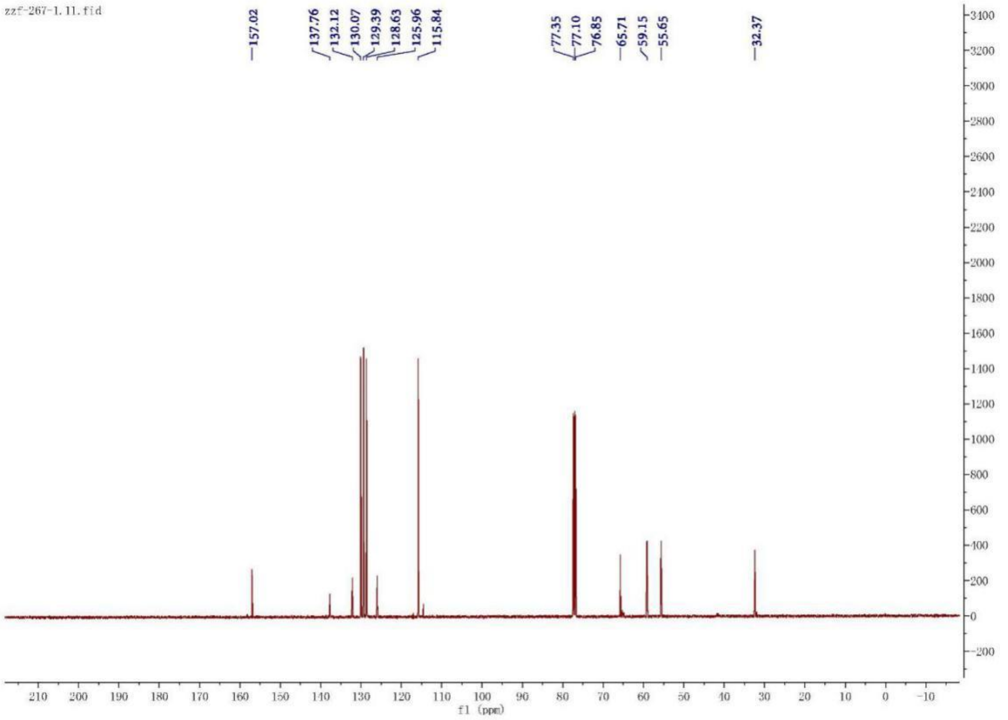

Supplementary Figure 78. ^{13}C NMR of product **3da**.

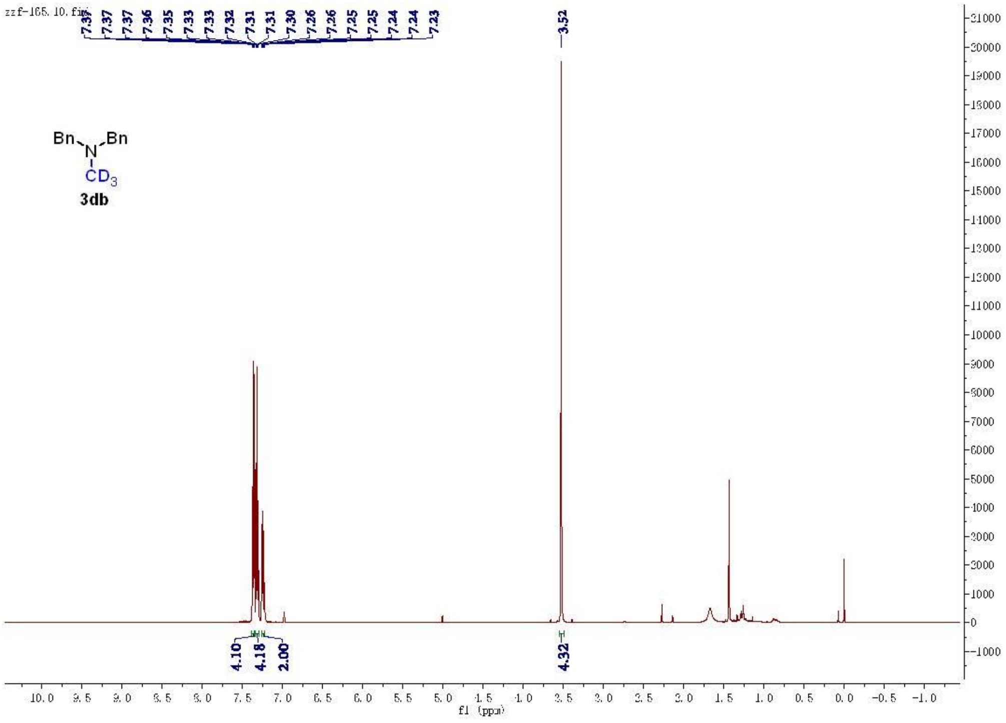

Supplementary Figure 79. ^{1}H NMR of product **3db**.

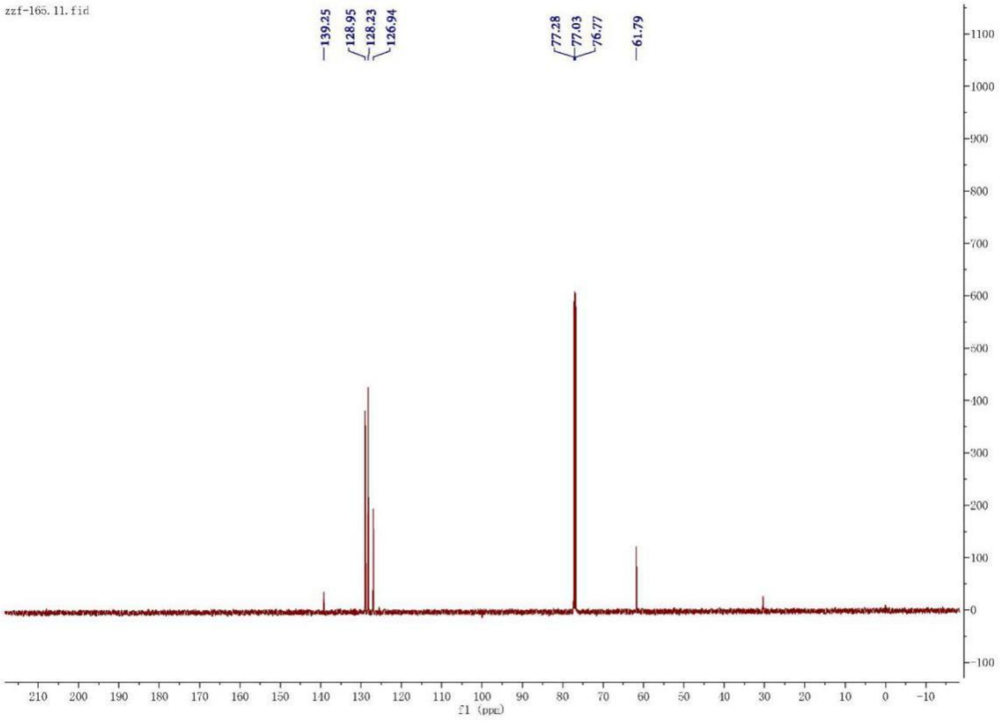

Supplementary Figure 80. ^{13}C NMR of product **3db**.

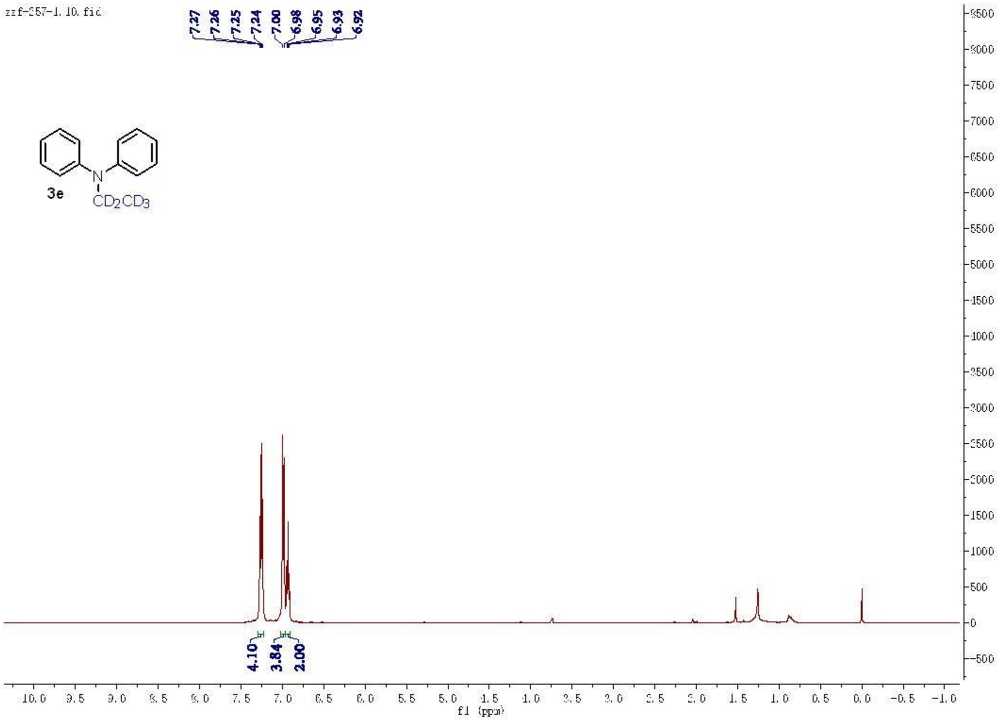

Supplementary Figure 81. ^{1}H NMR of product **3e**.

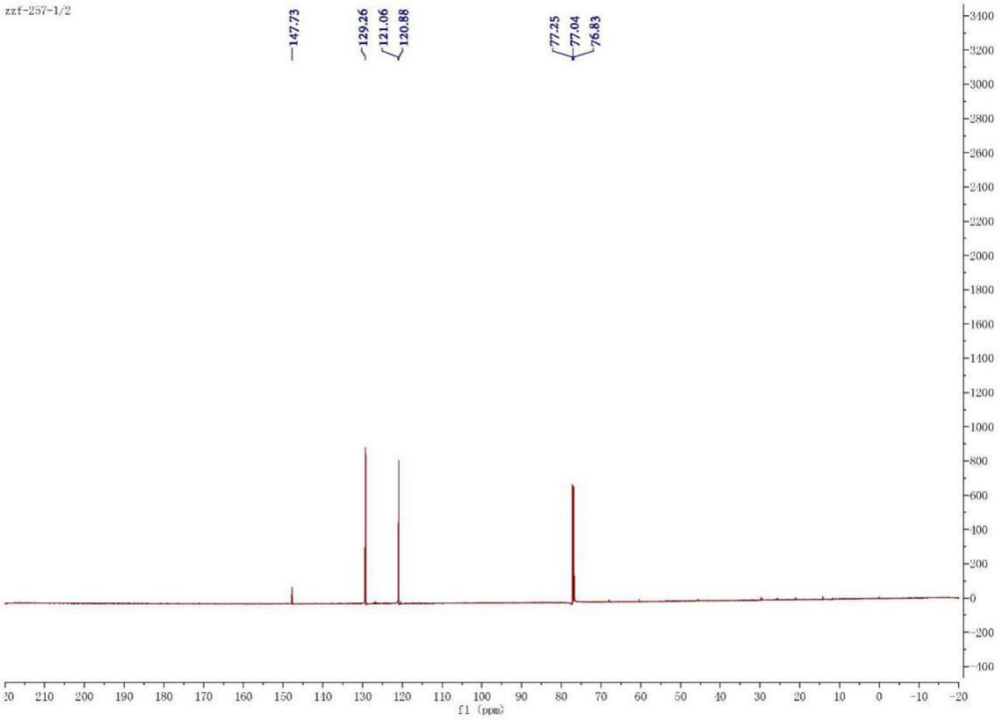

Supplementary Figure 82. ^{13}C NMR of product **3e**.

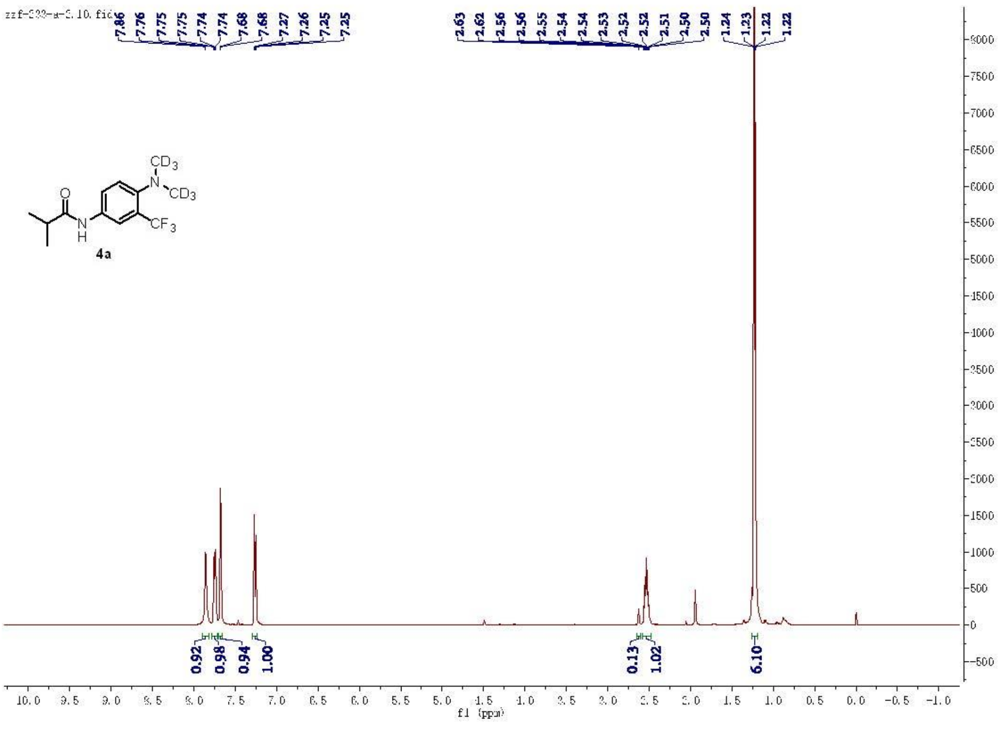

Supplementary Figure 83. ^{1}H NMR of product **4a**.

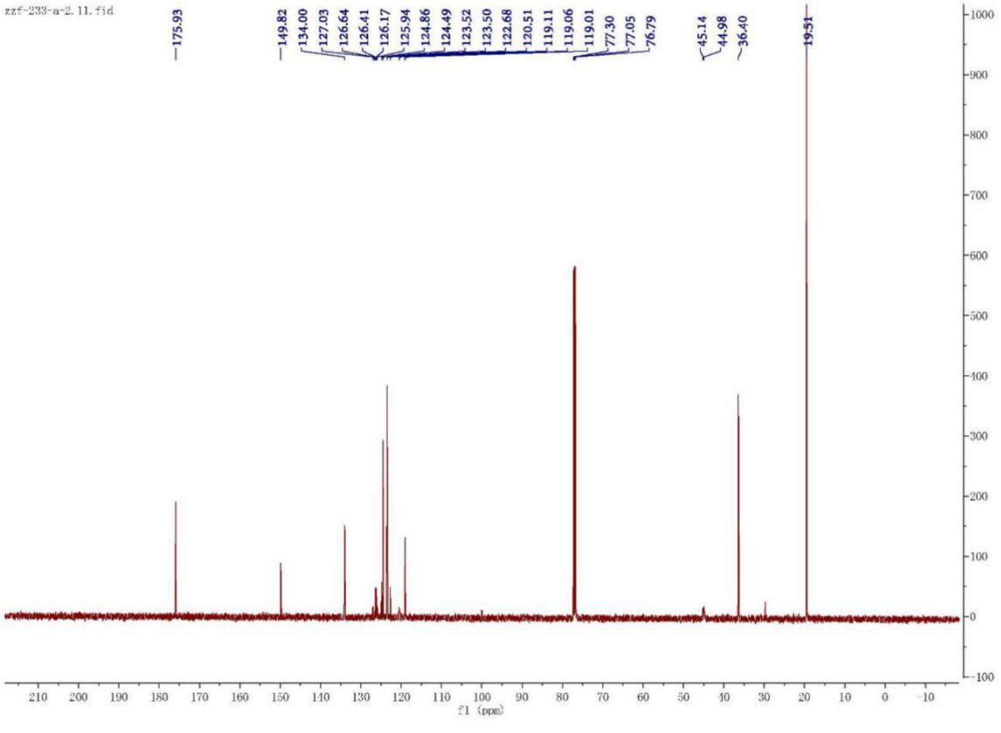

Supplementary Figure 84. ^{13}C NMR of product **4a**.

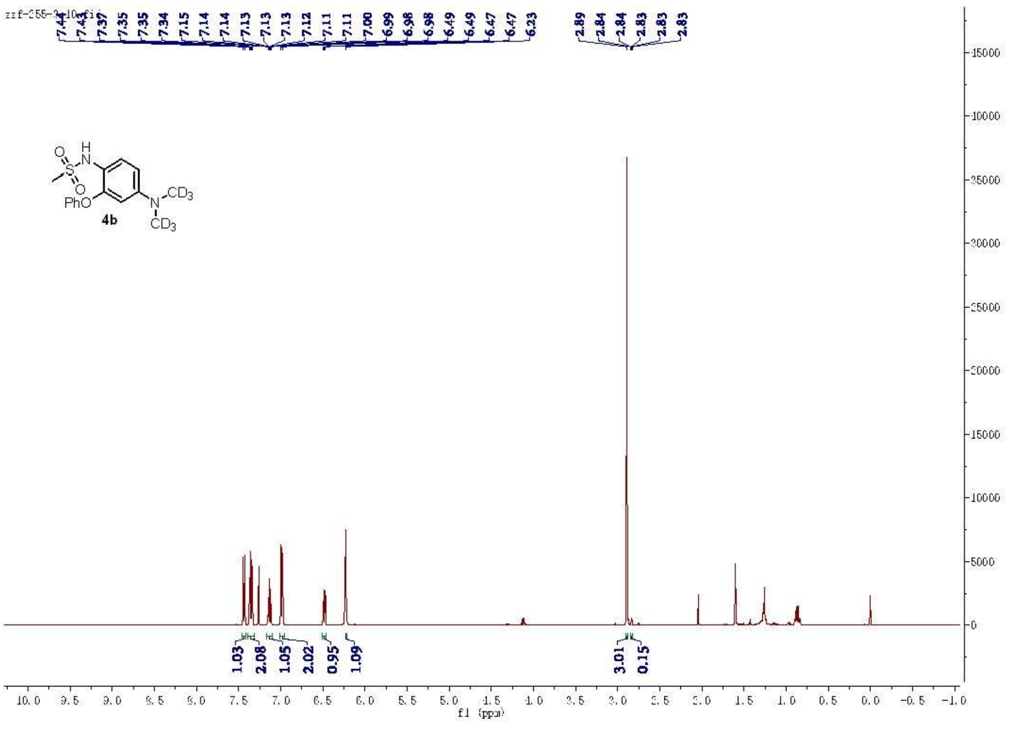

Supplementary Figure 85. ^{1}H NMR of product **4b**.

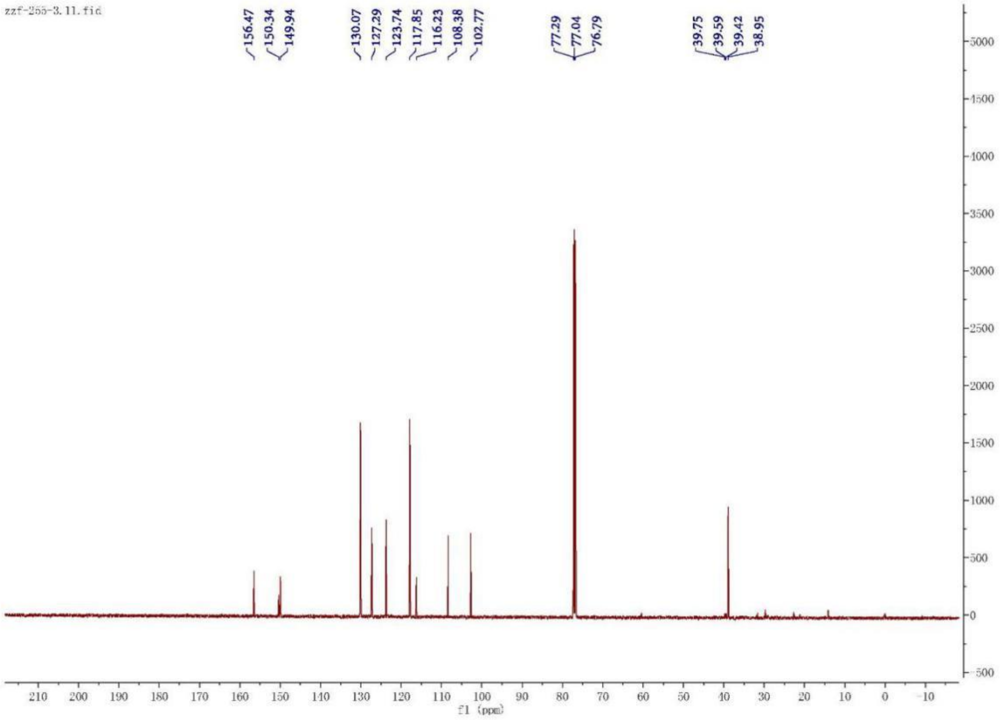

Supplementary Figure 86. ^{13}C NMR of product **4b**.

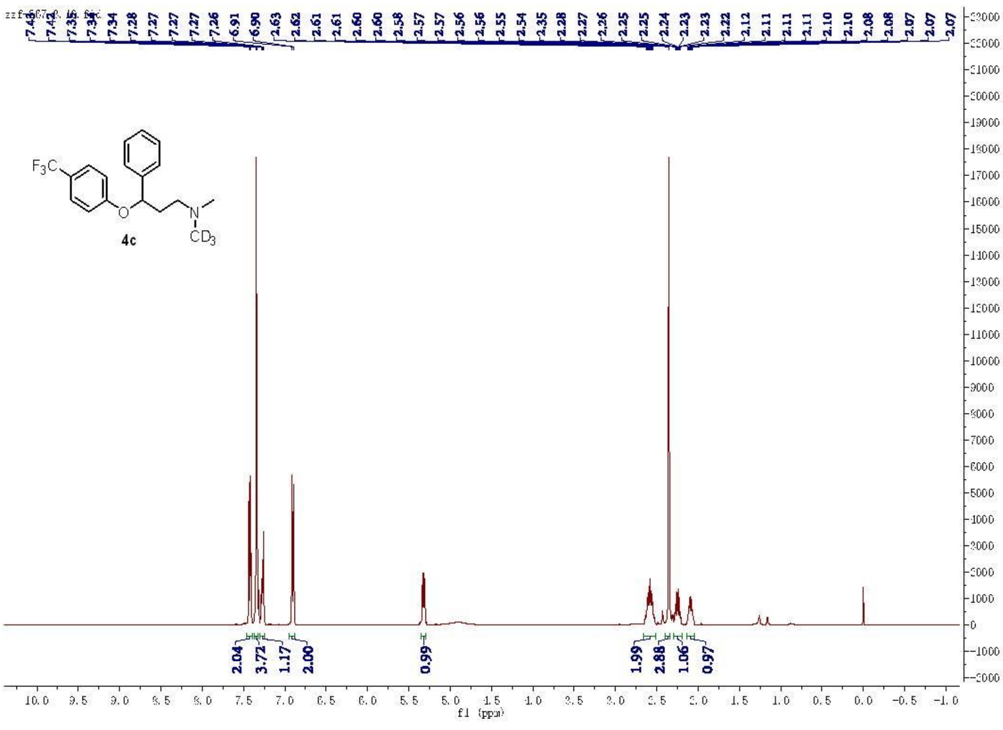

Supplementary Figure 87. ^{1}H NMR of product **4c**.

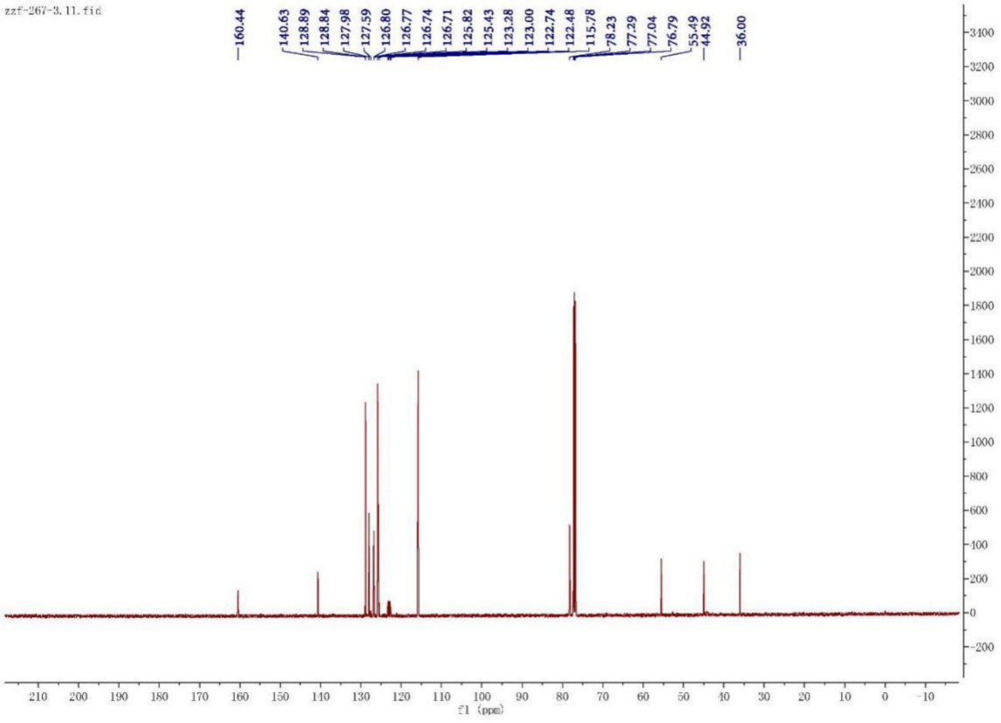

Supplementary Figure 88. ^{13}C NMR of product **4c**.

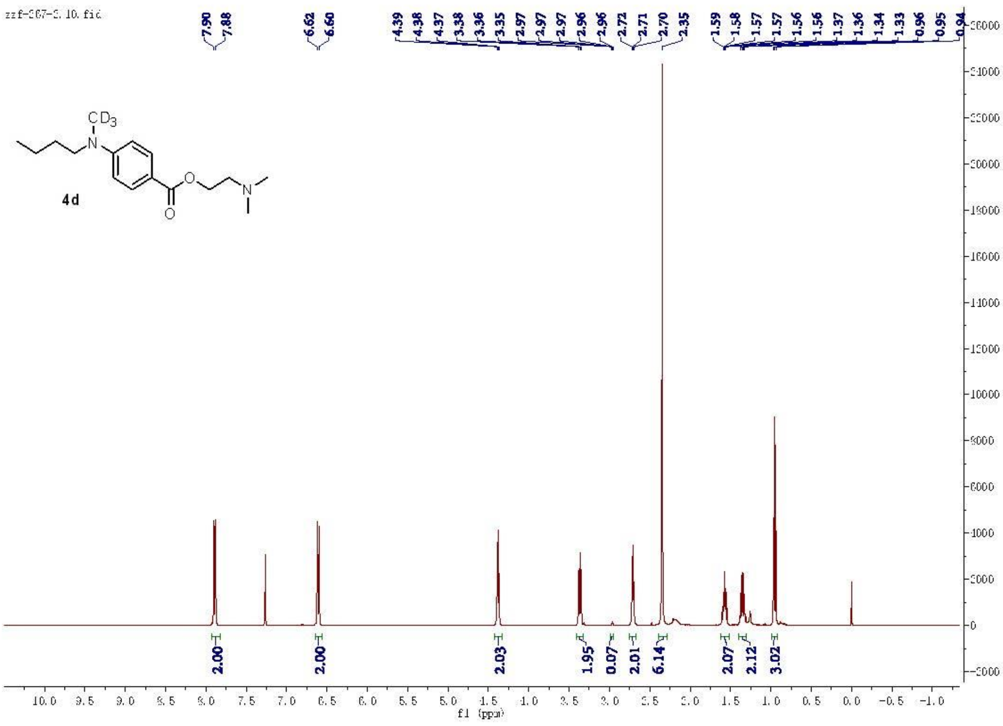

Supplementary Figure 89. ^{1}H NMR of product **4d**.

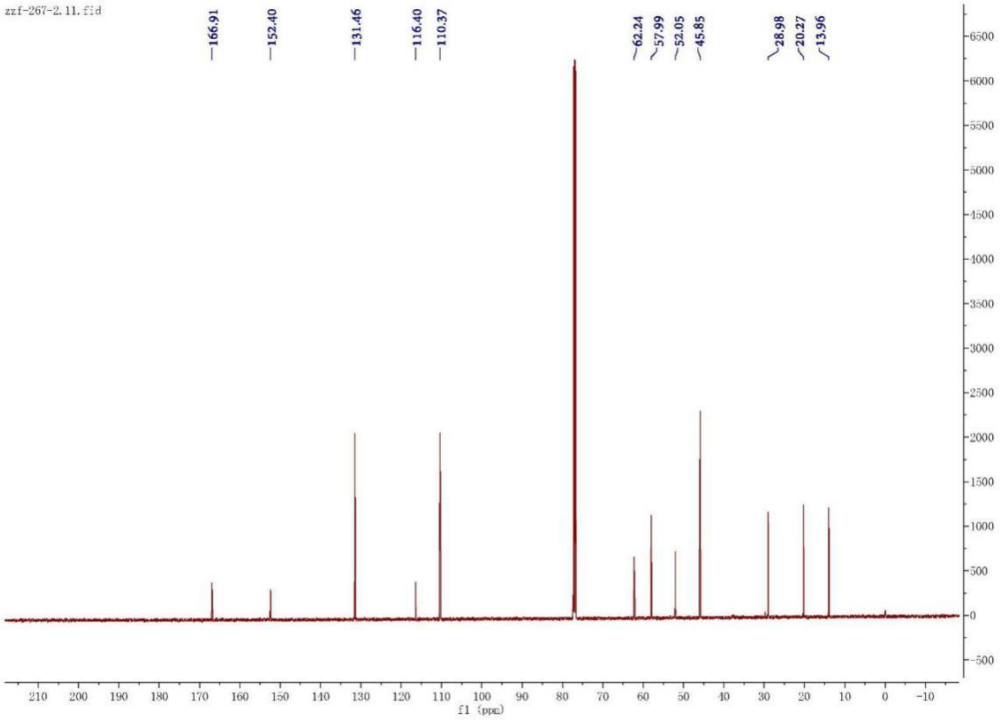

Supplementary Figure 90. ^{13}C NMR of product **4d**.

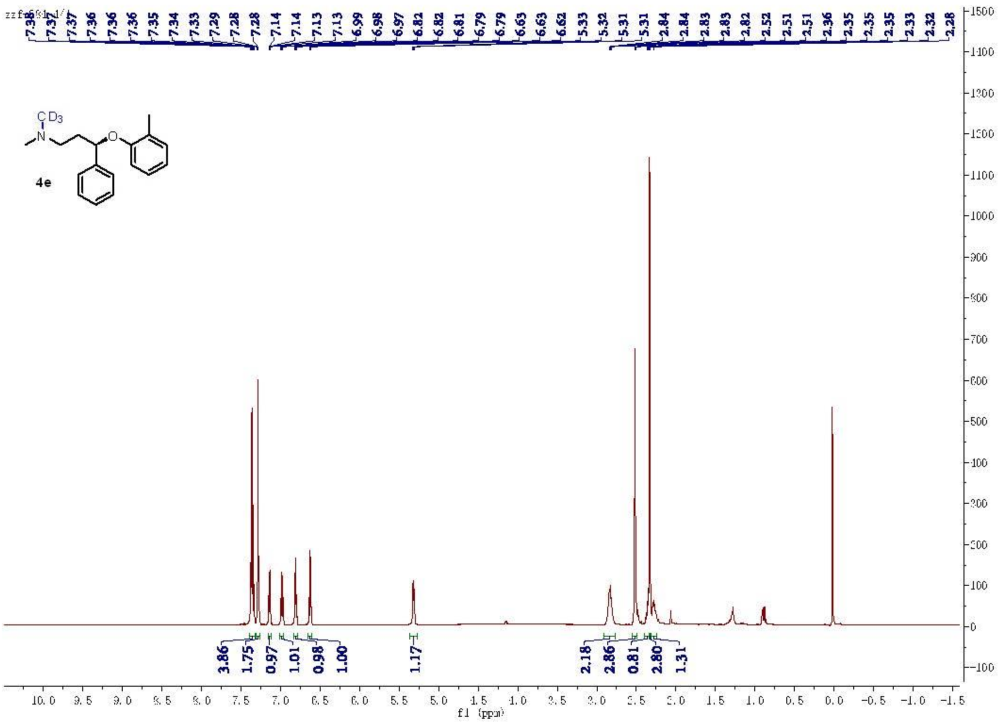

Supplementary Figure 91. ^{1}H NMR of product **4e**.

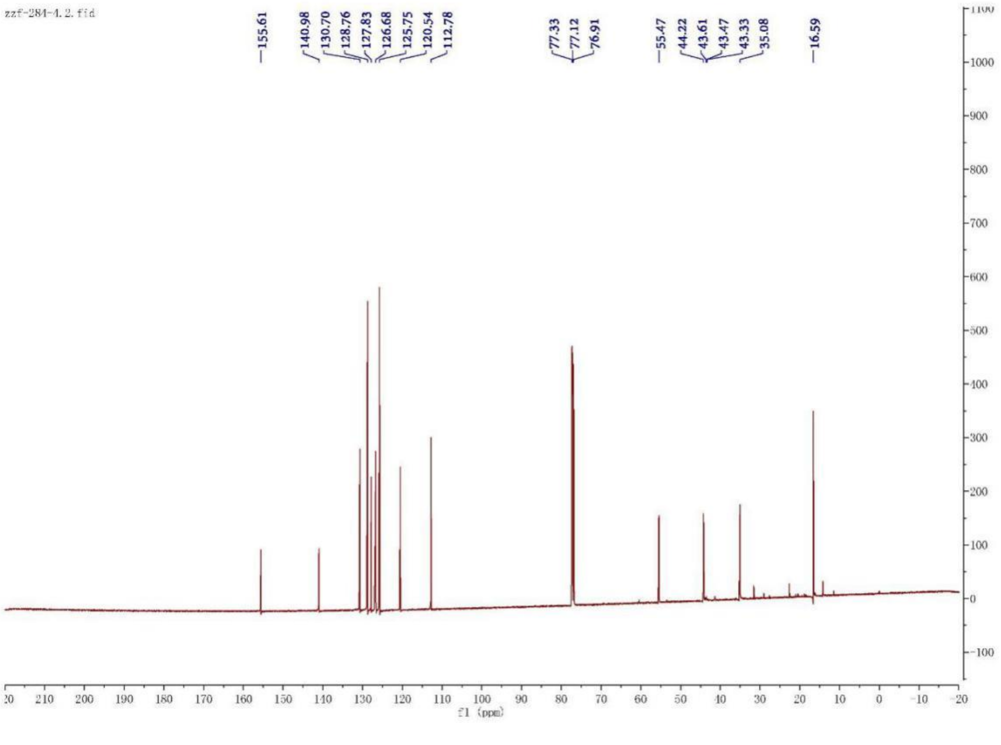

Supplementary Figure 92. ^{13}C NMR of product **4e**.

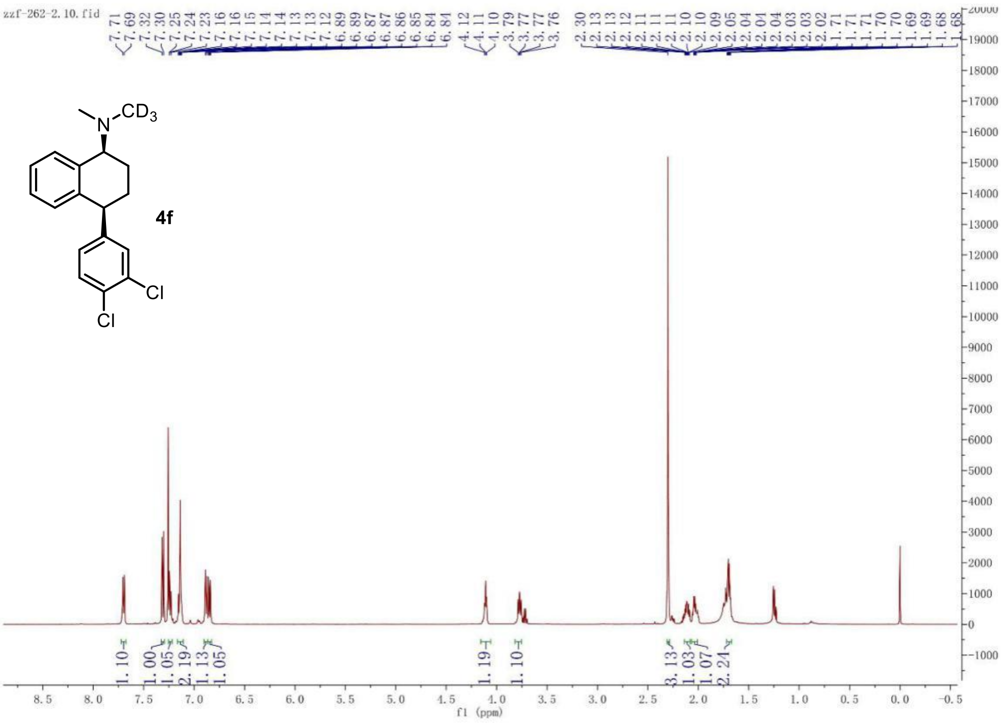

Supplementary Figure 93. ^{1}H NMR of product **4f**.

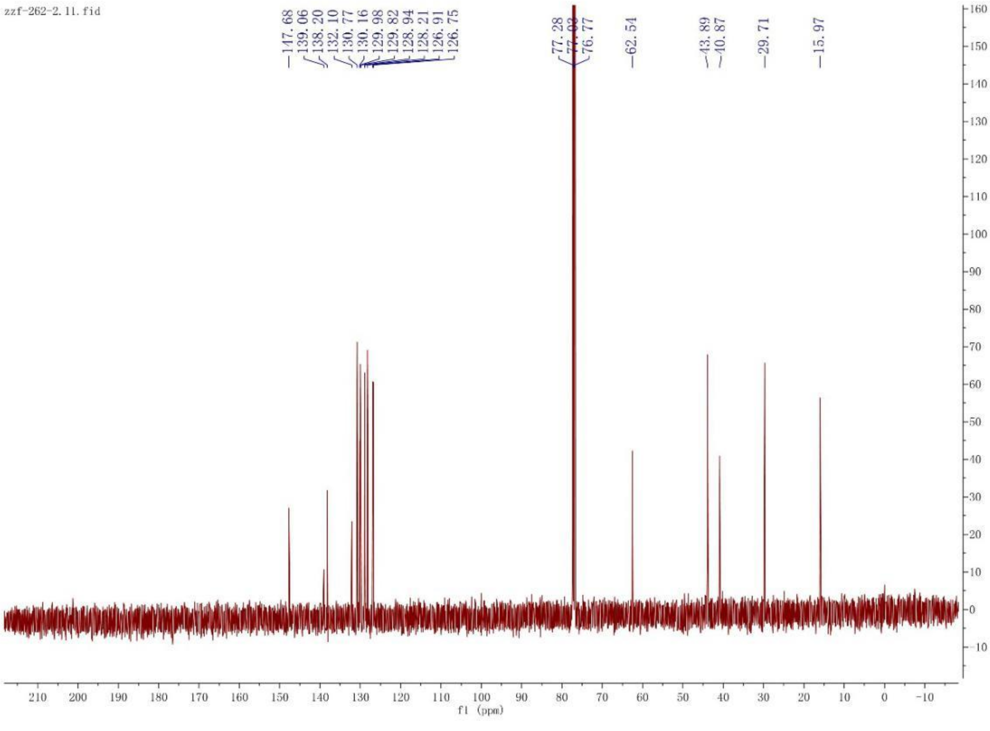

Supplementary Figure 94. ^{13}C NMR of product **4f**.

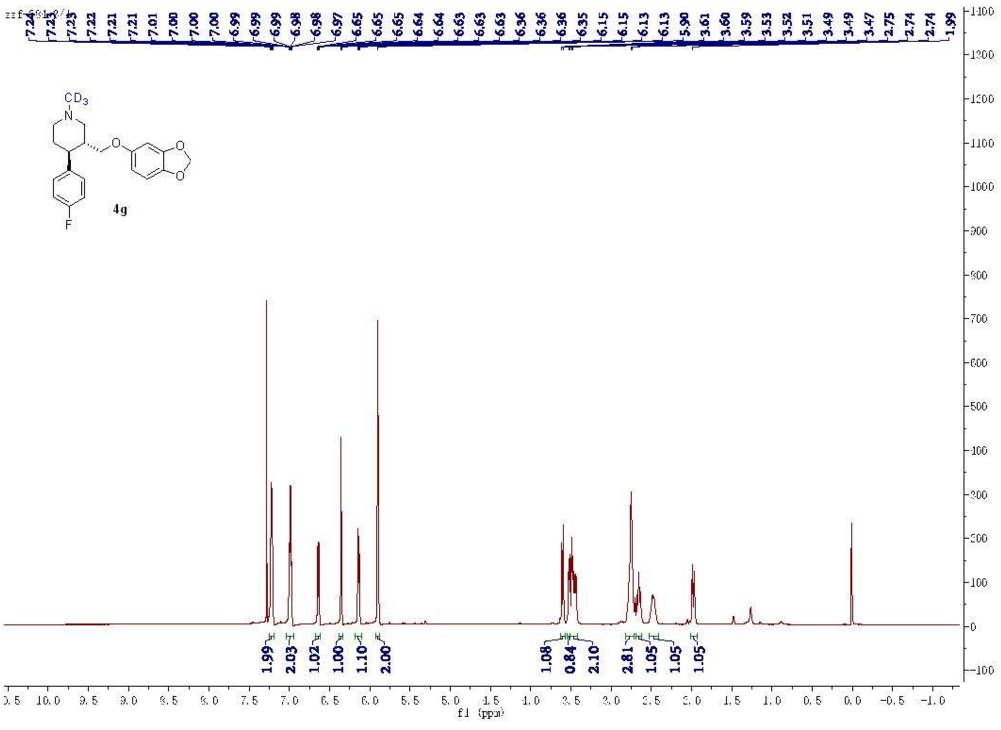

Supplementary Figure 95. ^{1}H NMR of product **4g**.

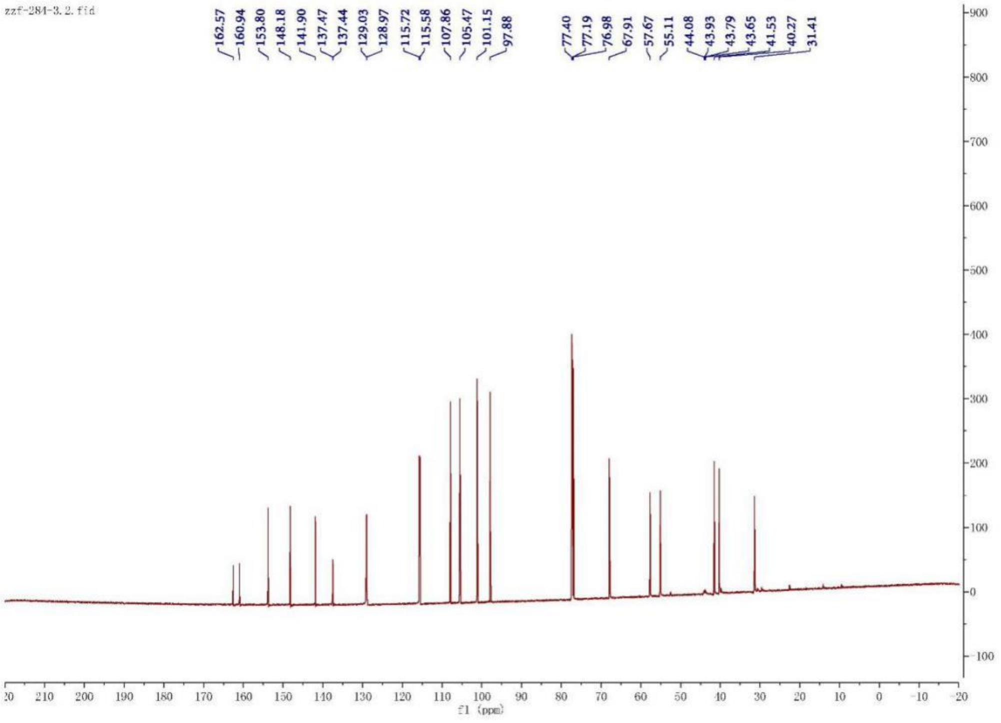

Supplementary Figure 96. ^{13}C NMR of product **4g**.

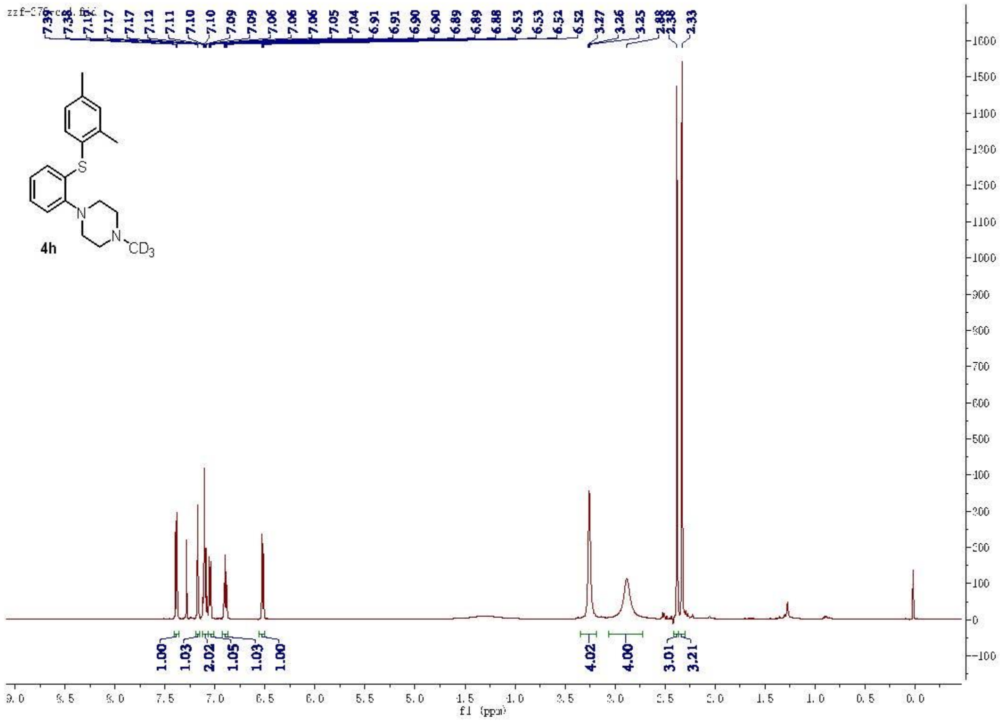

Supplementary Figure 97. ^{1}H NMR of product **4h**.

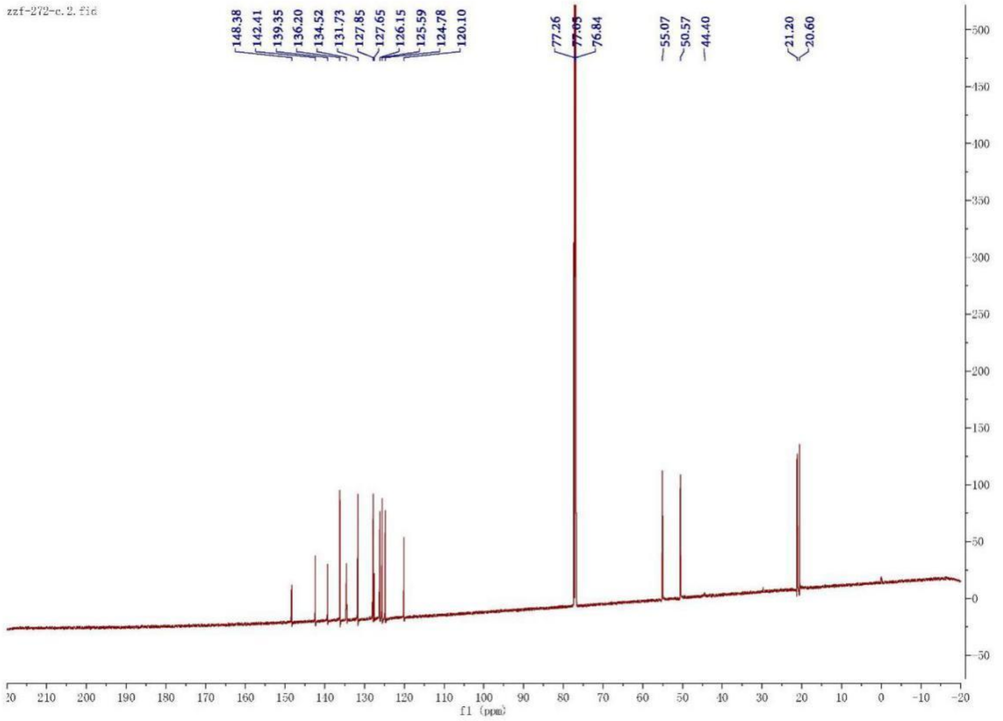

Supplementary Figure 98. ^{13}C NMR of product **4h**.

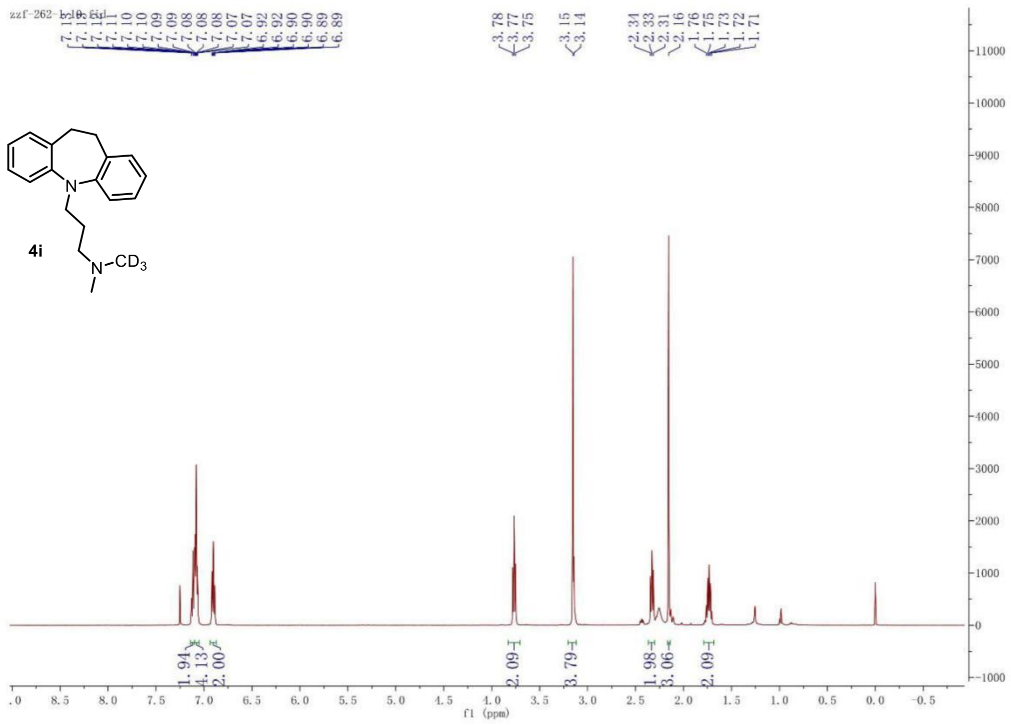

Supplementary Figure 99. ^{1}H NMR of product **4i**.

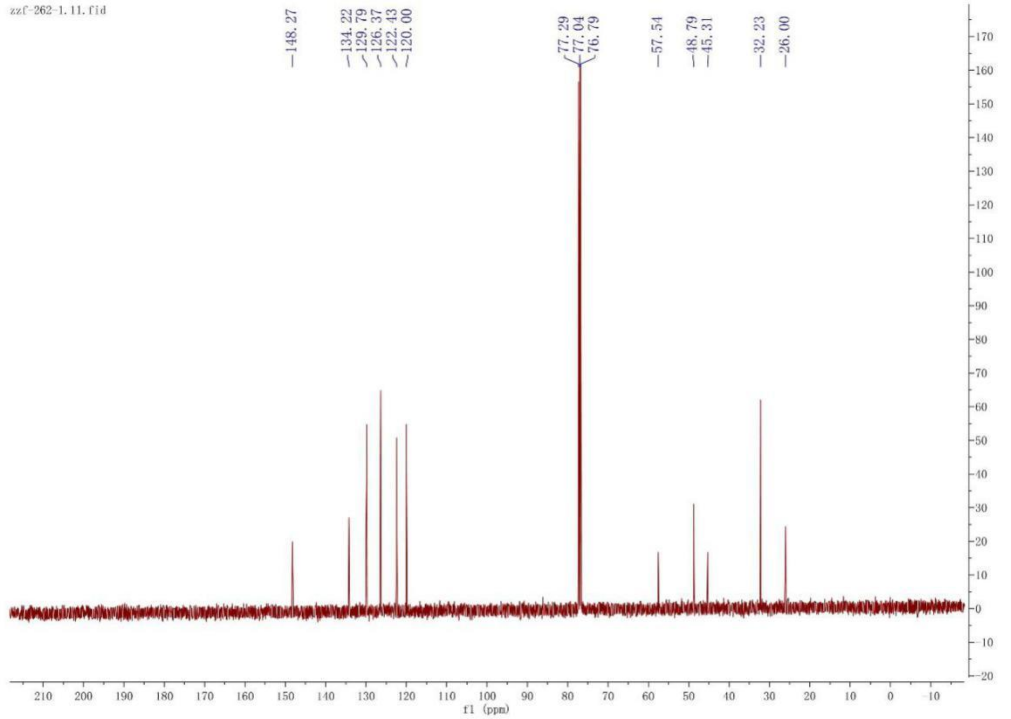

Supplementary Figure 100. ^{13}C NMR of product **4i**.

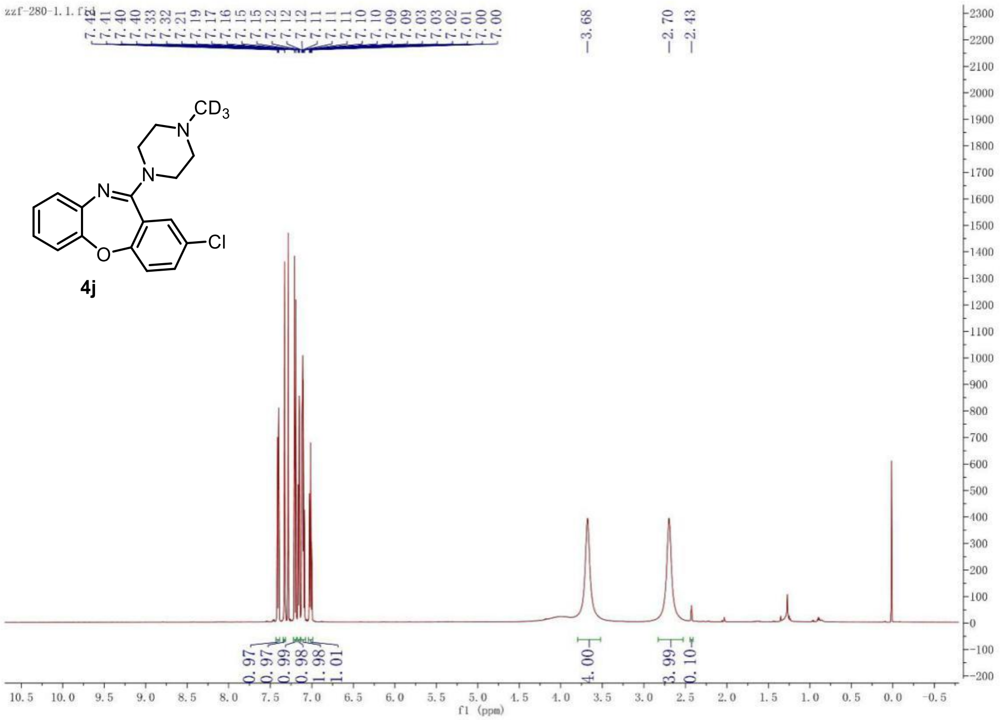

Supplementary Figure 101. ^{1}H NMR of product **4j**.

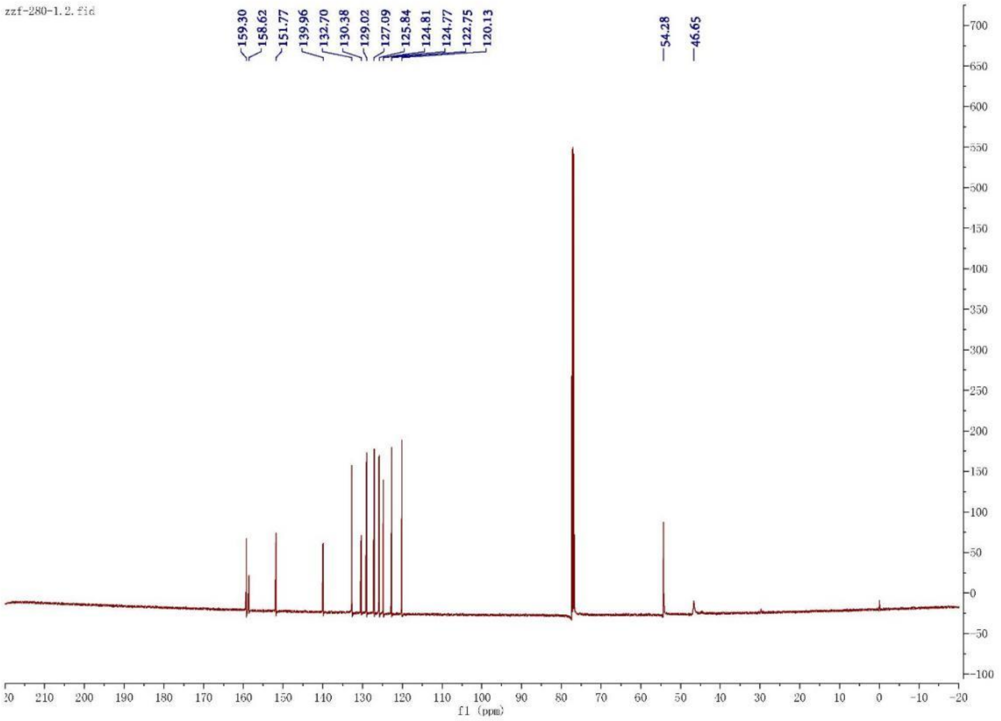

Supplementary Figure 102. ^{13}C NMR of product **4j**.

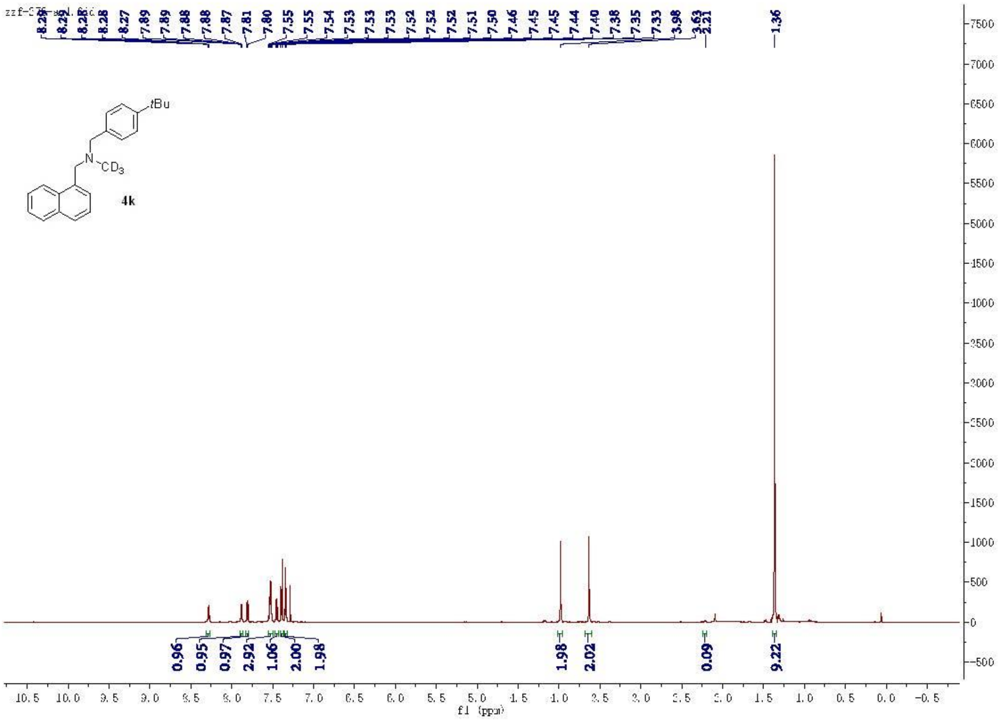

Supplementary Figure 103. ^{1}H NMR of product **4k**.

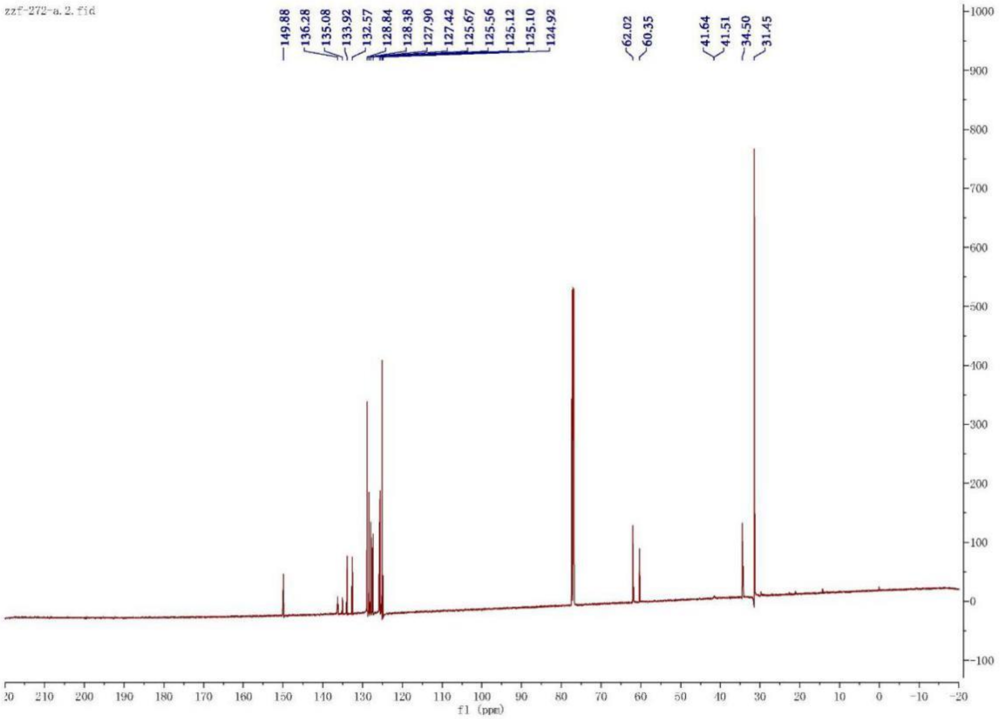

Supplementary Figure 104. ^{13}C NMR of product **4k**.

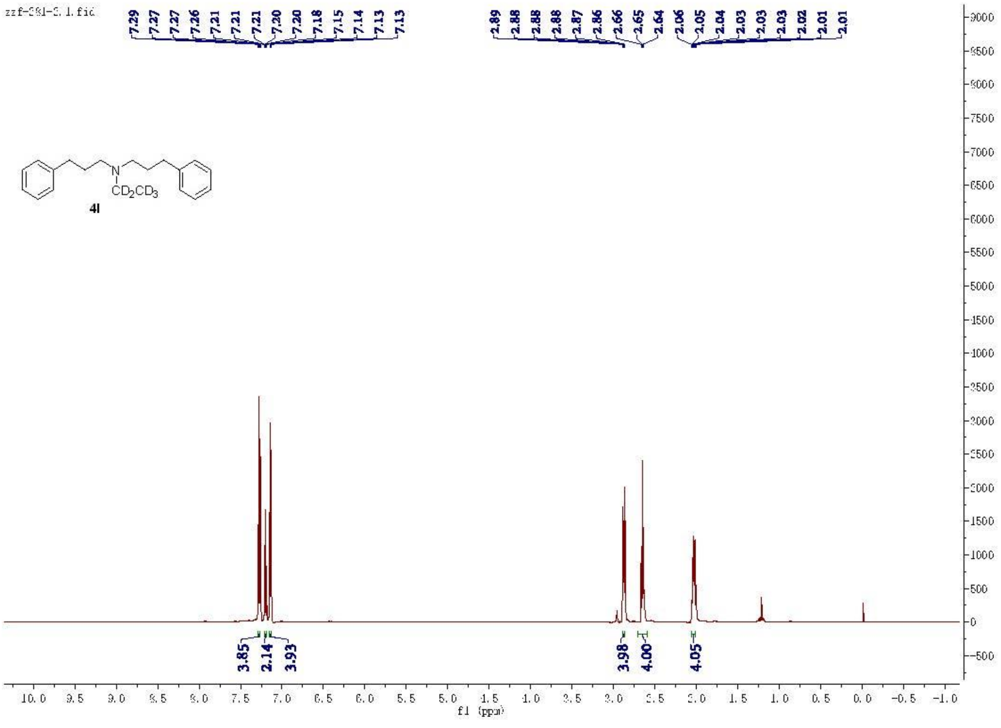

Supplementary Figure 105. ^{1}H NMR of product **4l**.

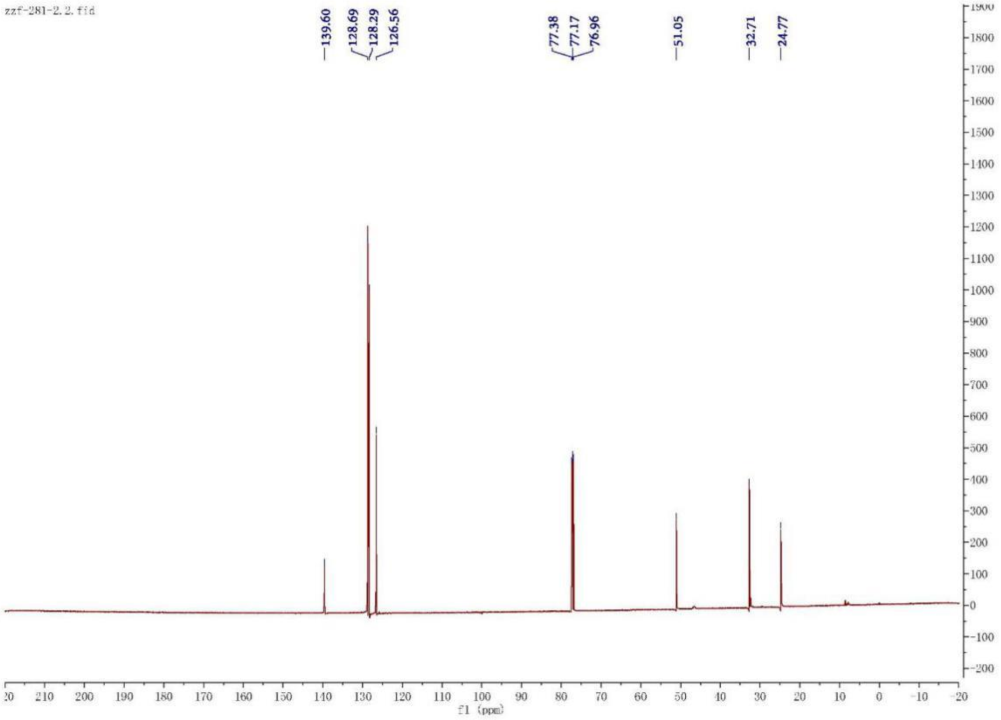

Supplementary Figure 106. ^{13}C NMR of product **4l**.

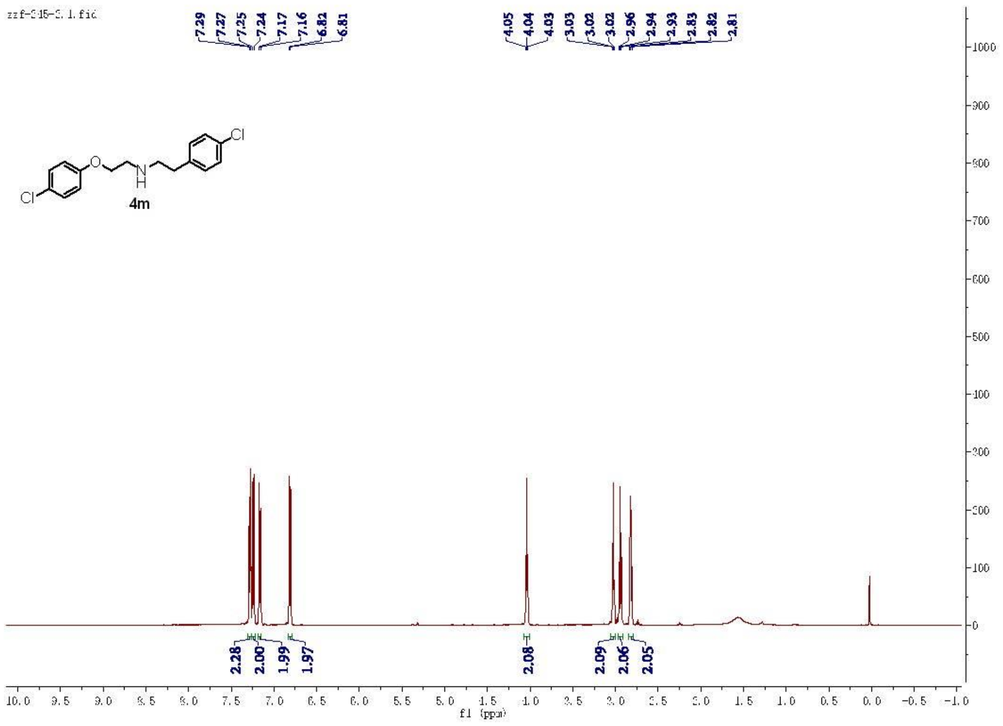

Supplementary Figure 107. ^{1}H NMR of product **4m**.

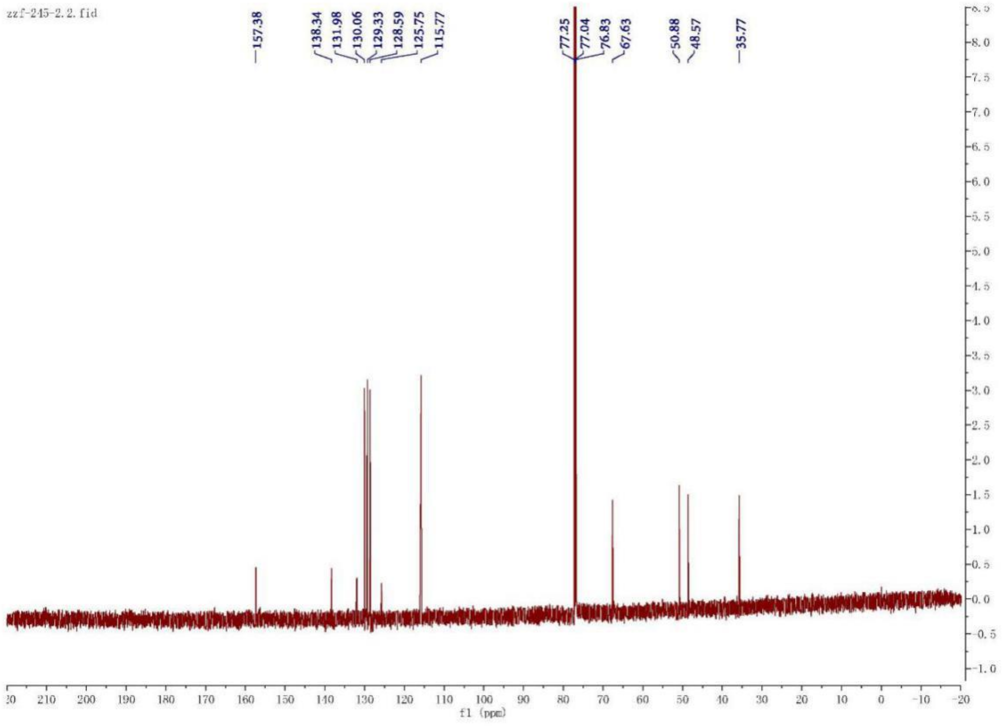

Supplementary Figure 108. ^{13}C NMR of product **4m**.

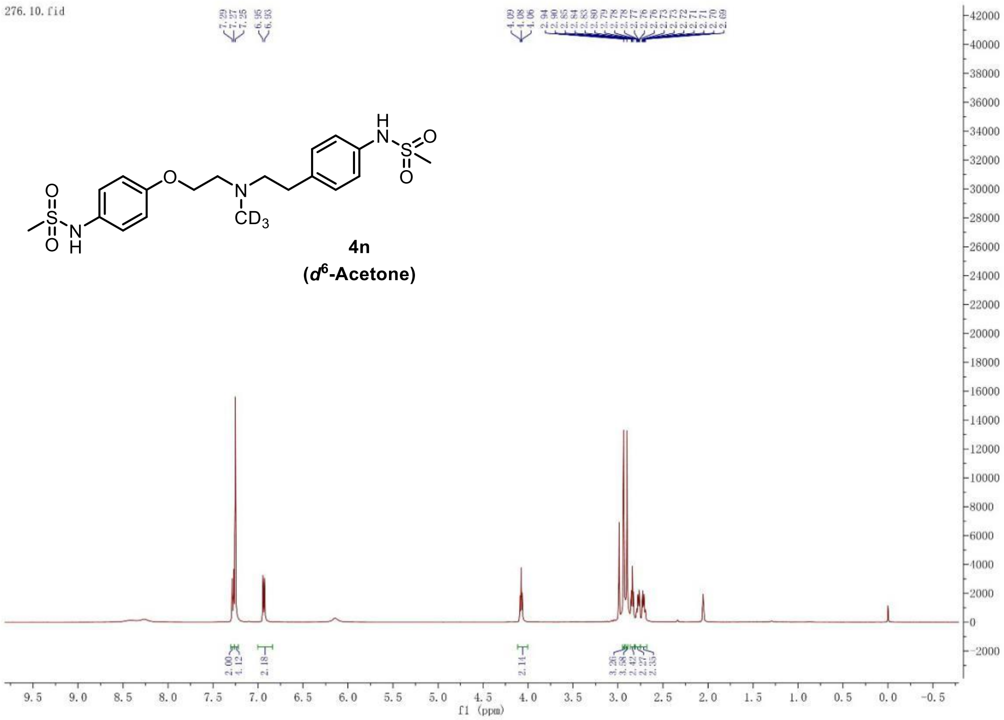

Supplementary Figure 109. ^{1}H NMR of product **4n**.

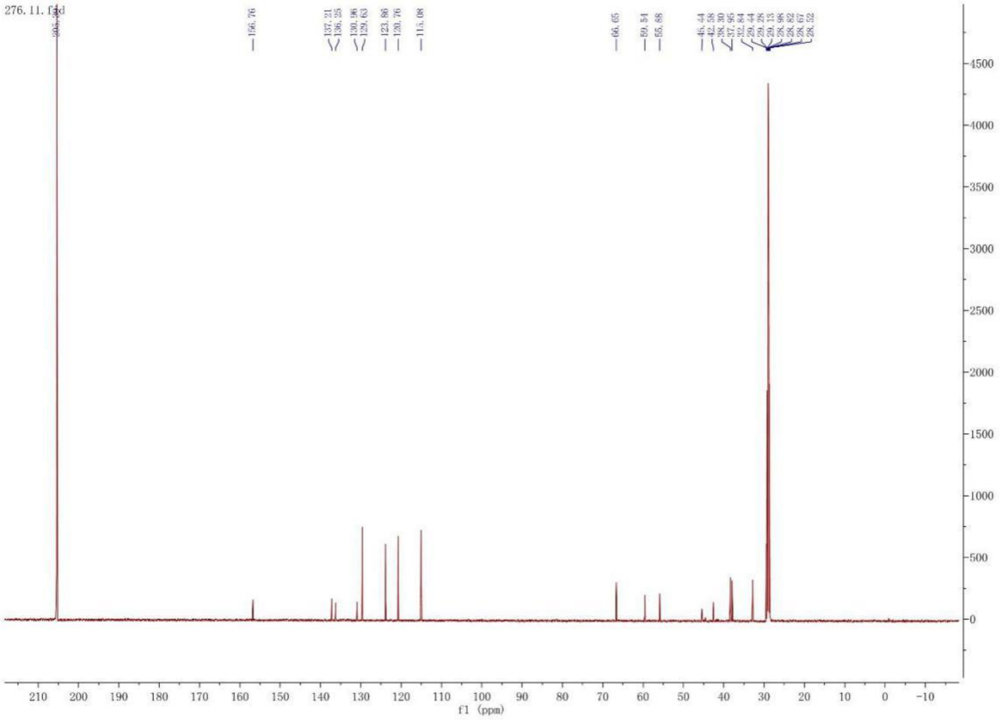

Supplementary Figure 110. ^{13}C NMR of product **4n**.

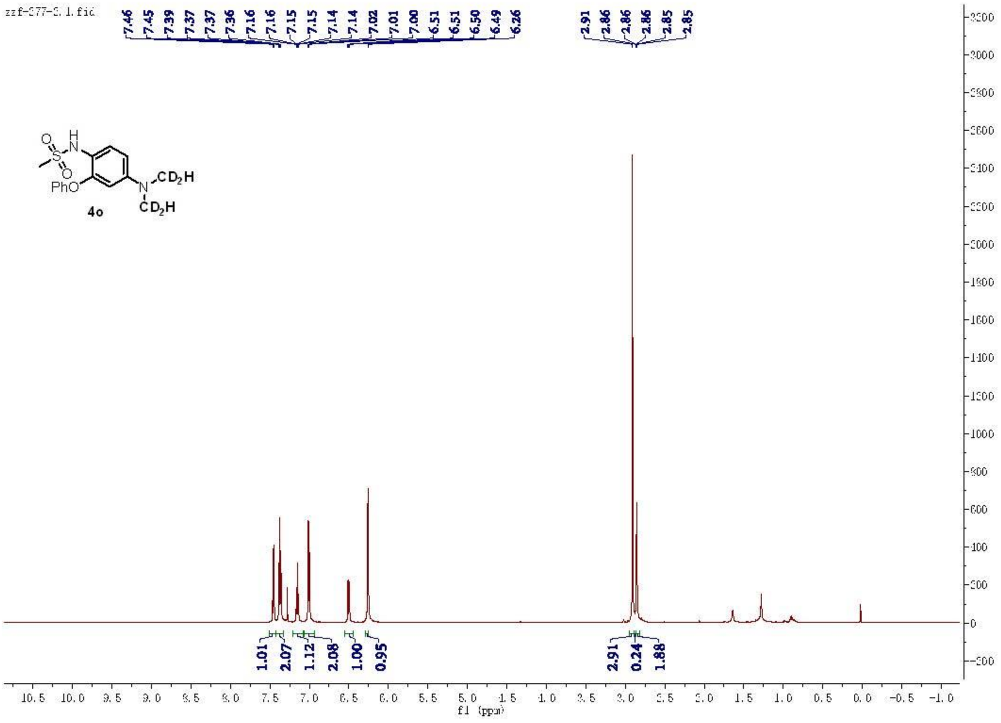

Supplementary Figure 111. ^{1}H NMR of product **4o**.

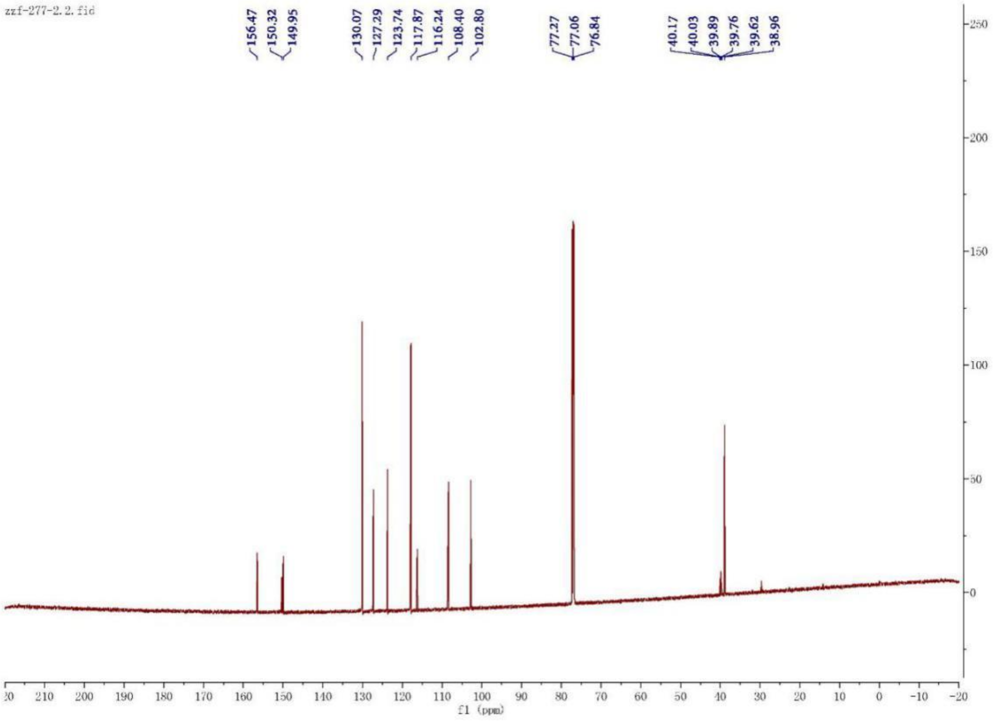

Supplementary Figure 112. ^{13}C NMR of product **4o**.

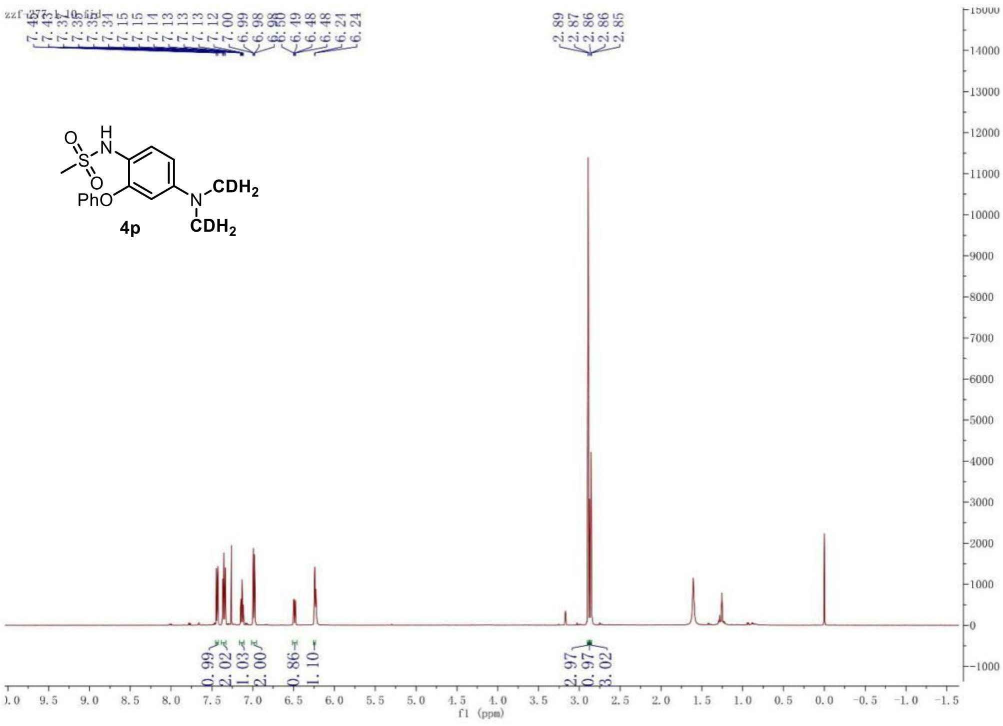

Supplementary Figure 113. ^{1}H NMR of product **4p**.

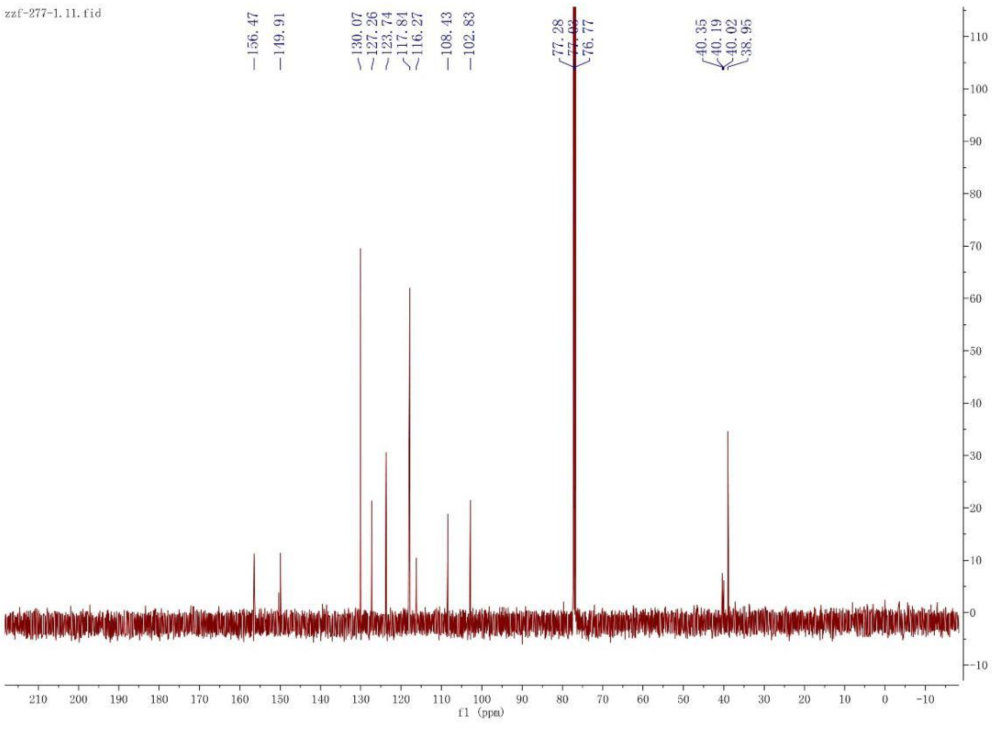

Supplementary Figure 114. ^{13}C NMR of product **4p**.

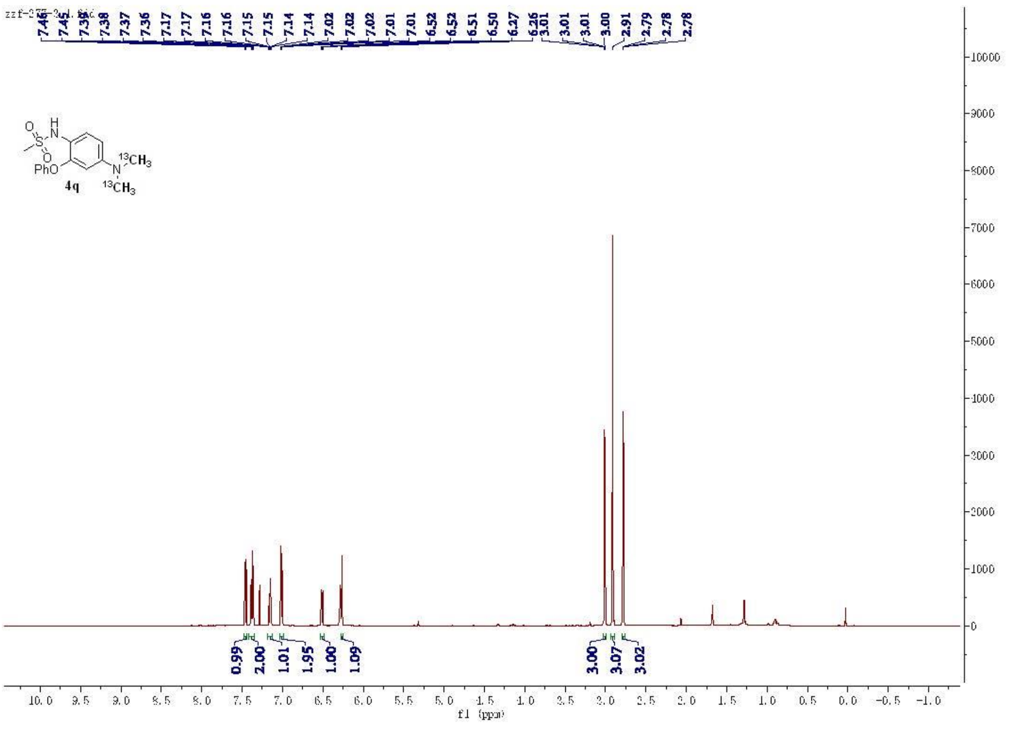

Supplementary Figure 115. ^{1}H NMR of product **4q**.

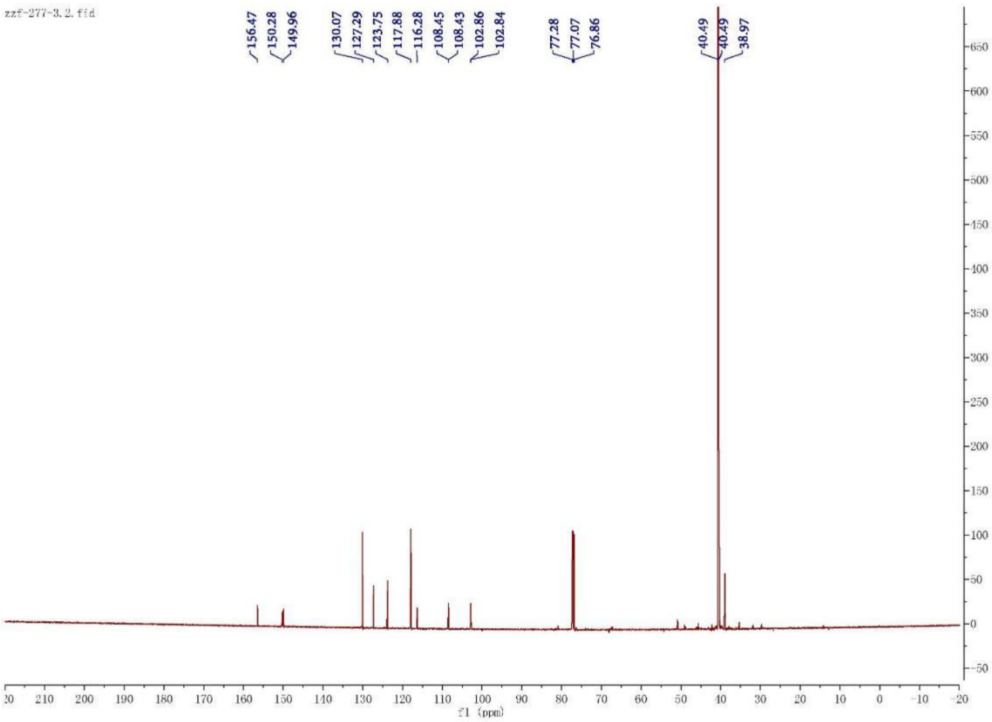

Supplementary Figure 116. ^{13}C NMR of product **4q**.

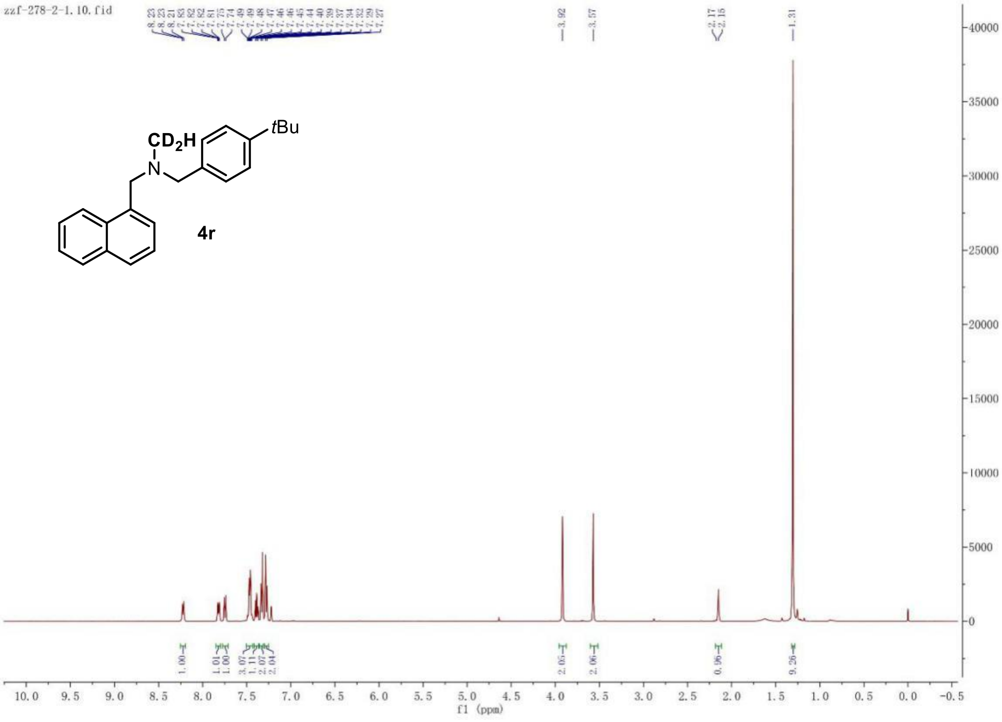

Supplementary Figure 117. ^{1}H NMR of product **4r**.

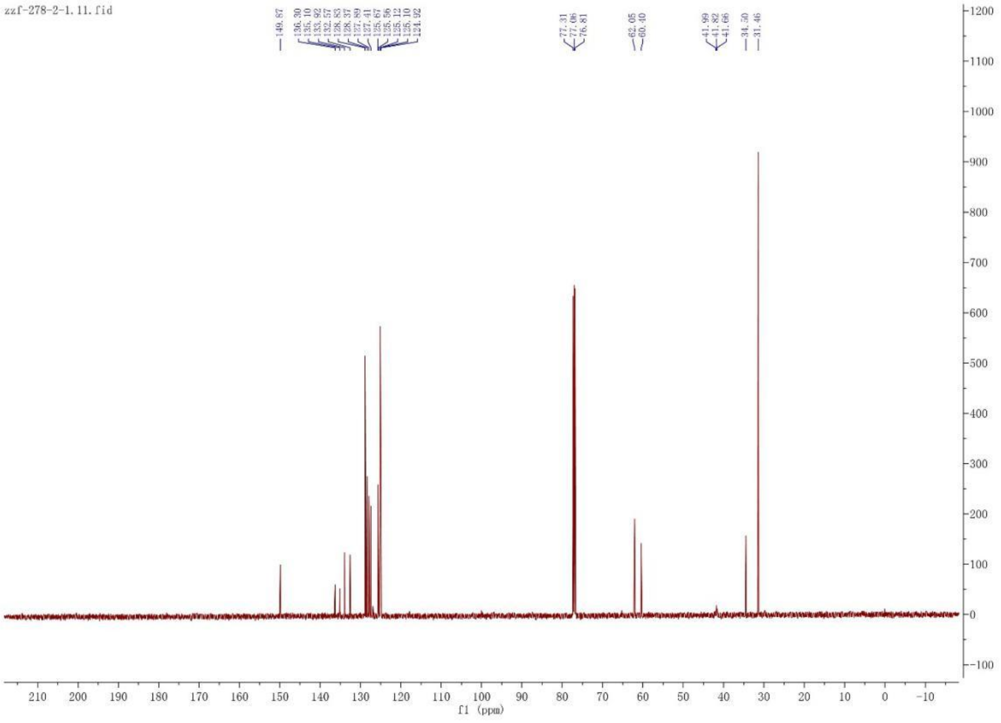

Supplementary Figure 118. ^{13}C NMR of product **4r**.

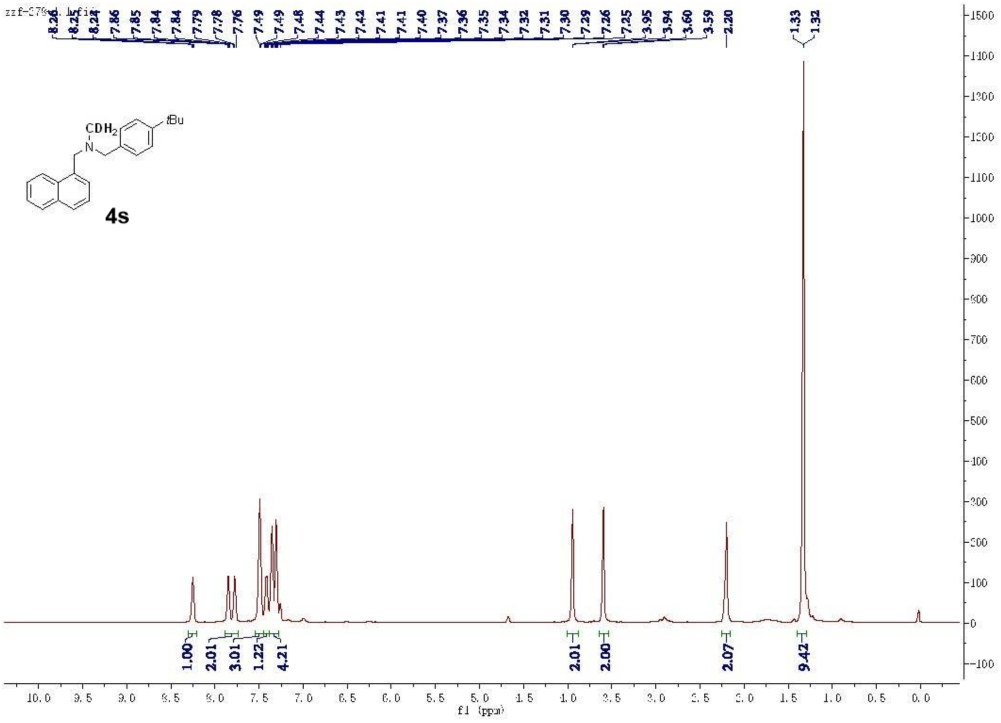

Supplementary Figure 119. ^{1}H NMR of product **4s**.

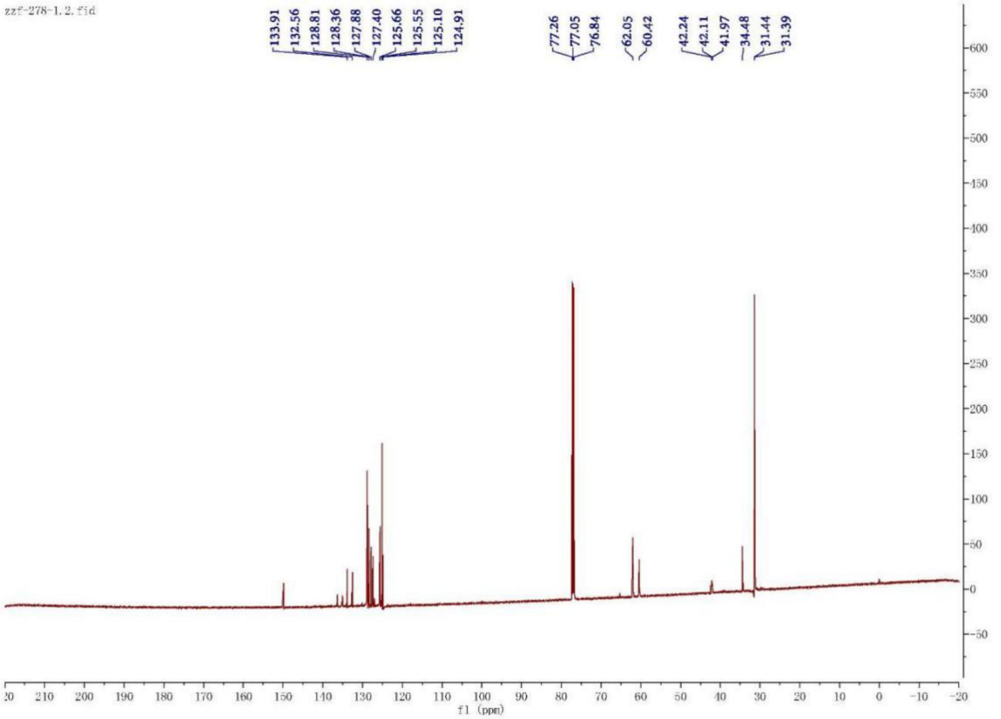

Supplementary Figure 120. ^{13}C NMR of product **4s**.

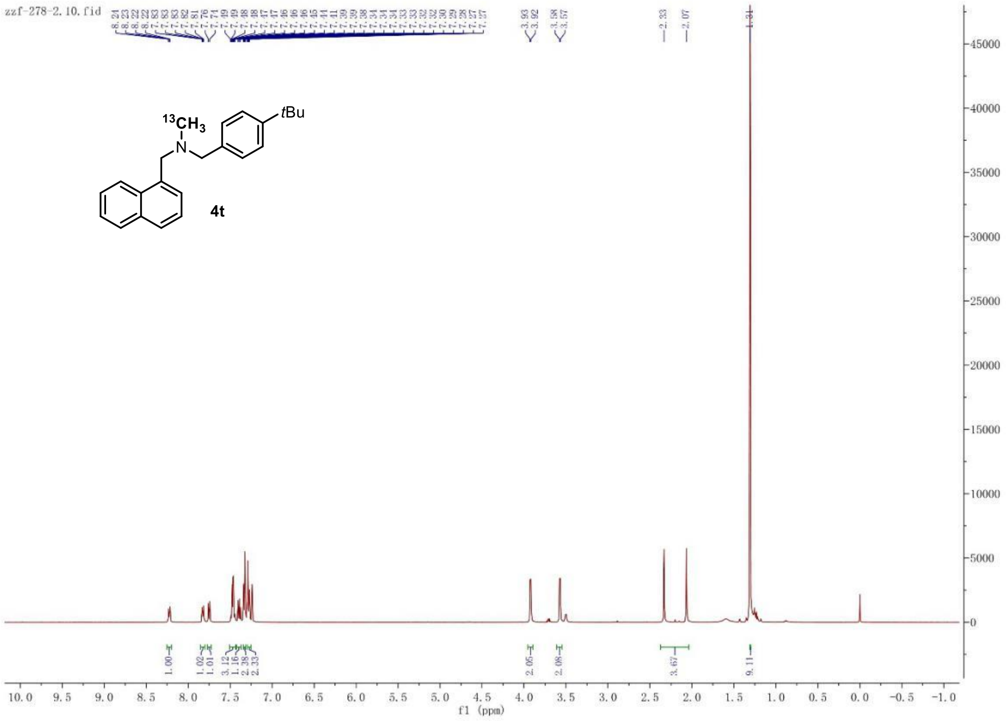

Supplementary Figure 121. ^{1}H NMR of product **4t**.

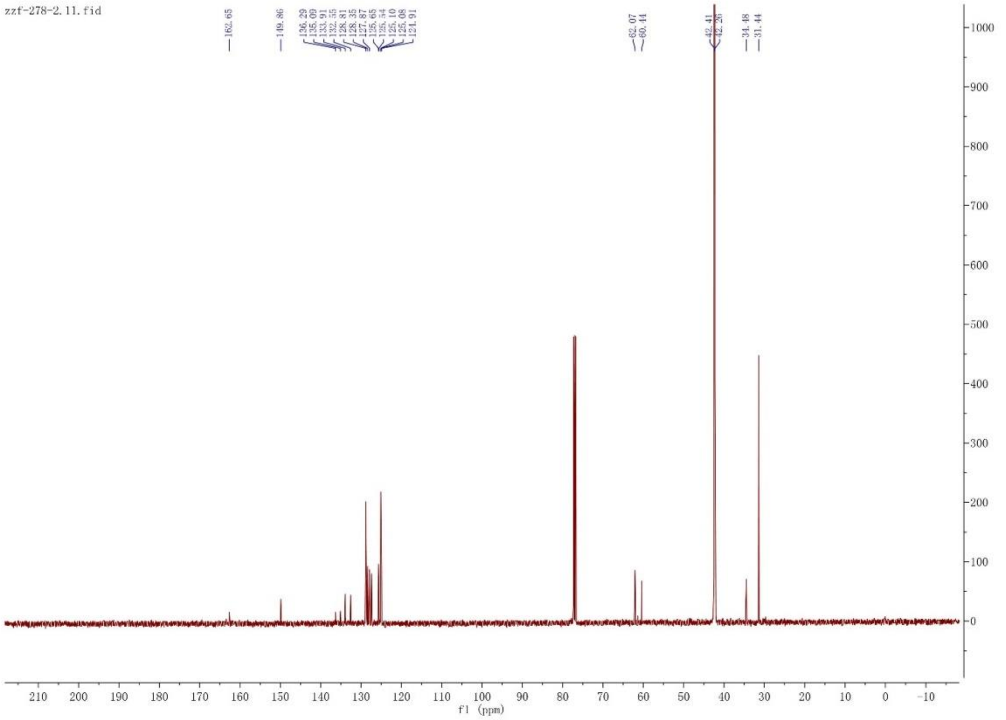

Supplementary Figure 122. ^{13}C NMR of product **4t**.

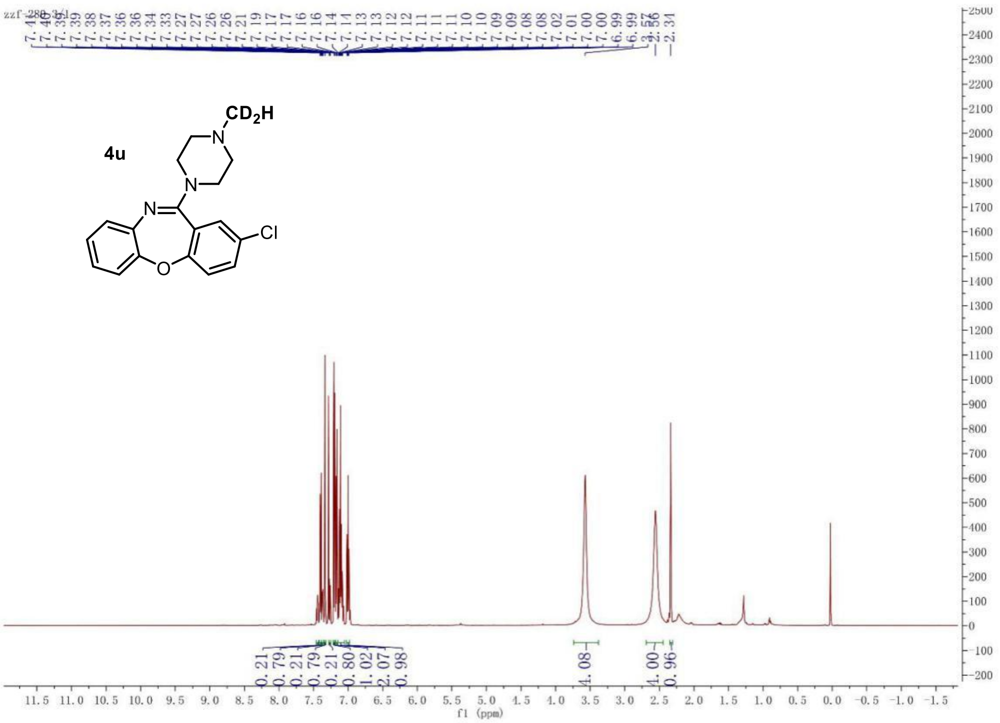

Supplementary Figure 123. ^{1}H NMR of product **4u**.

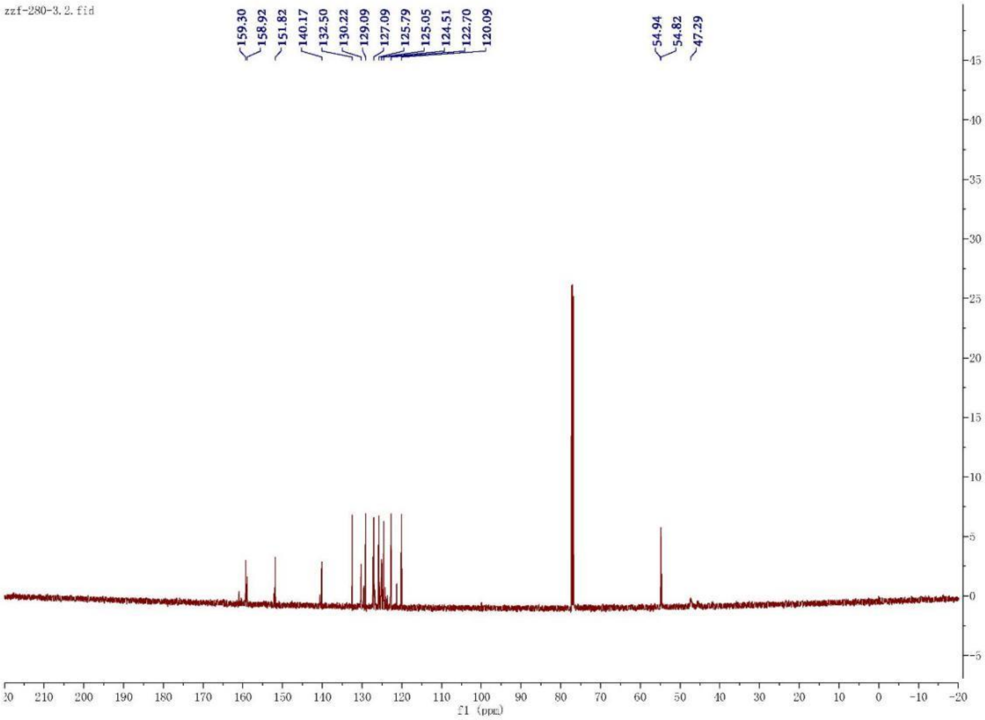

Supplementary Figure 124. ^{13}C NMR of product **4u**.

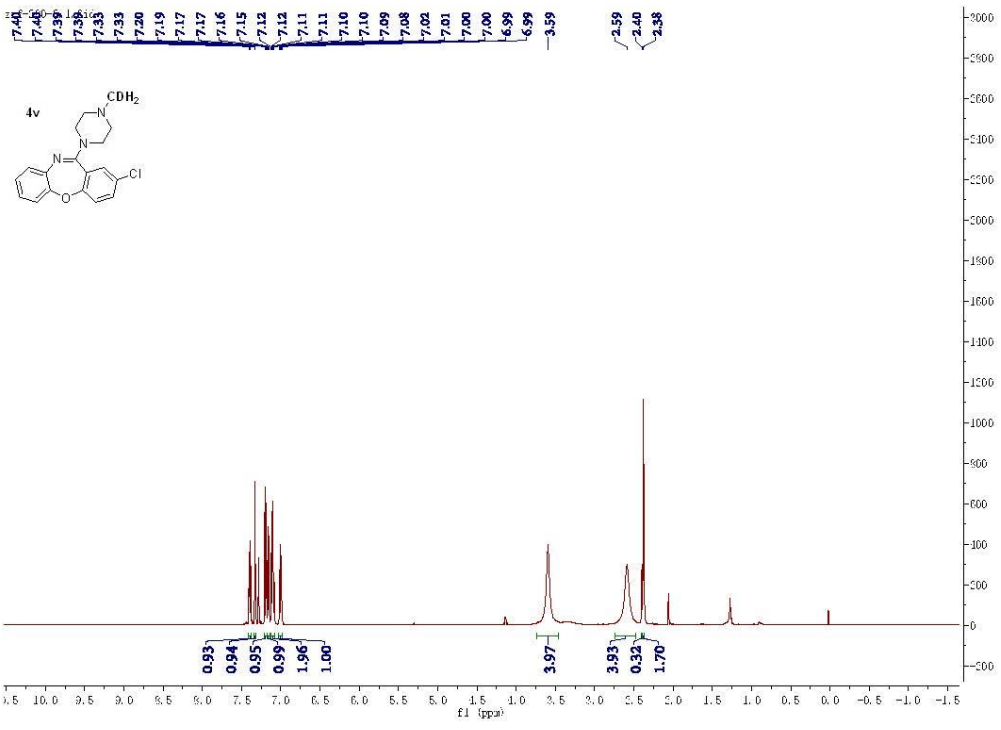

Supplementary Figure 125. ^{1}H NMR of product **4v**.

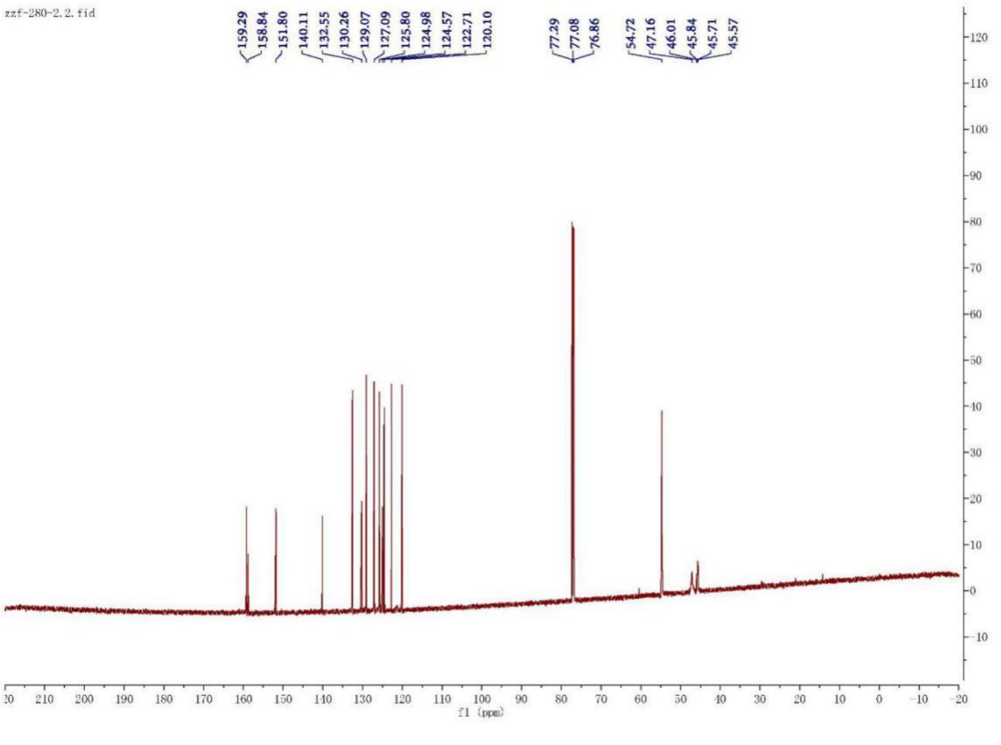

Supplementary Figure 126. ^{13}C NMR of product **4v**.

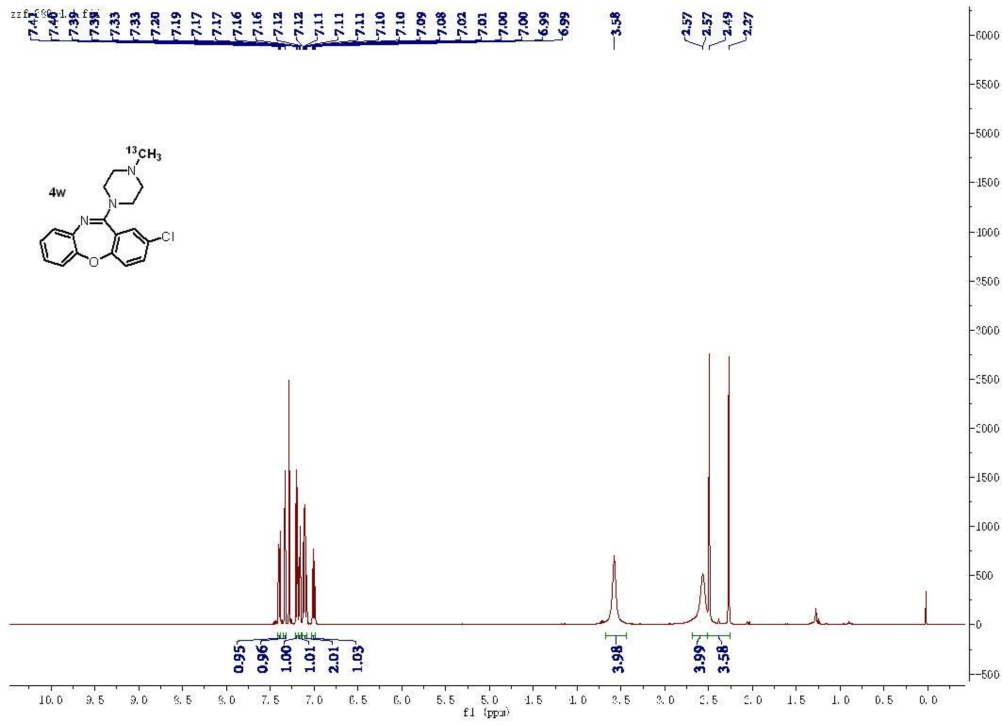

Supplementary Figure 127. ^{1}H NMR of product **4w**.

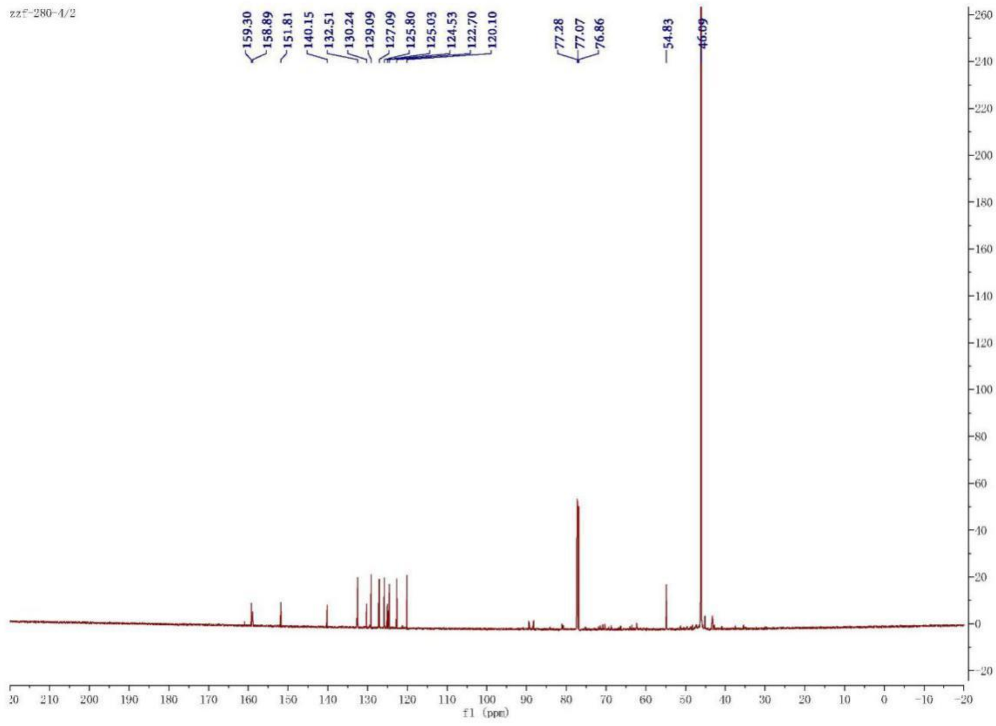

Supplementary Figure 128. ^{13}C NMR of product **4w**.

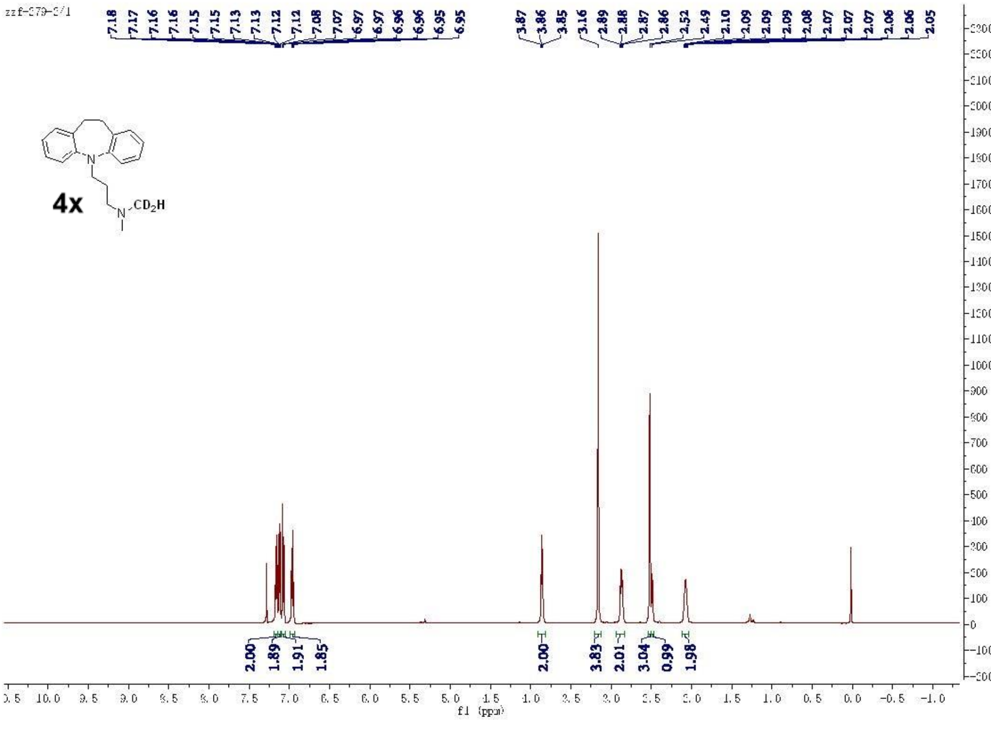

Supplementary Figure 129. ^{1}H NMR of product **4x**.

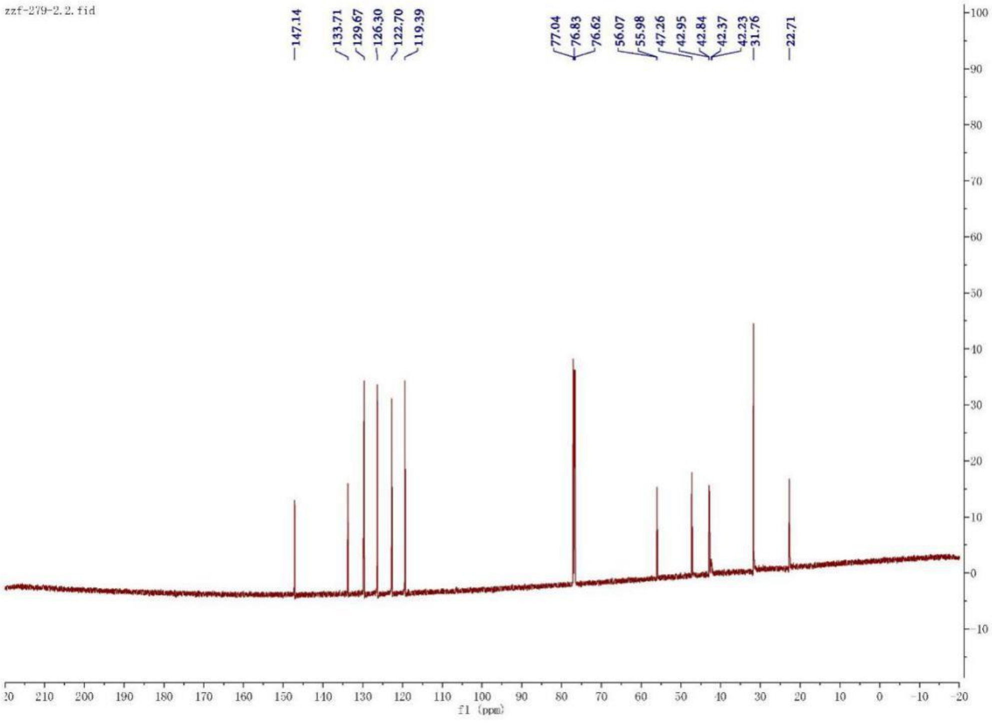

Supplementary Figure 130. ^{13}C NMR of product **4x**.

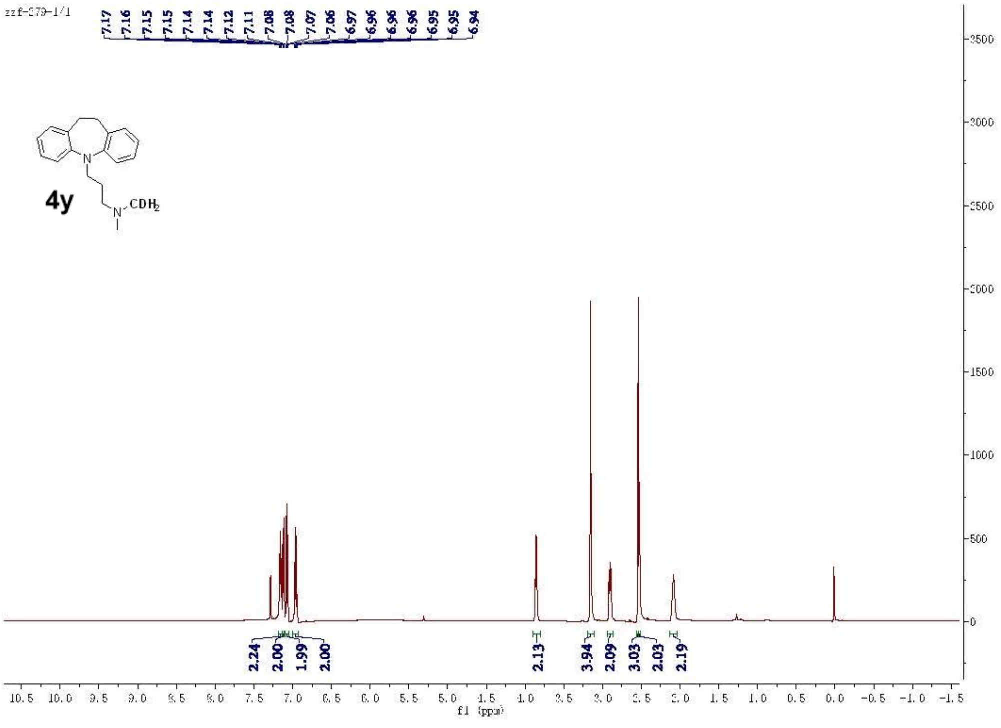

Supplementary Figure 131. ^{1}H NMR of product **4y**.

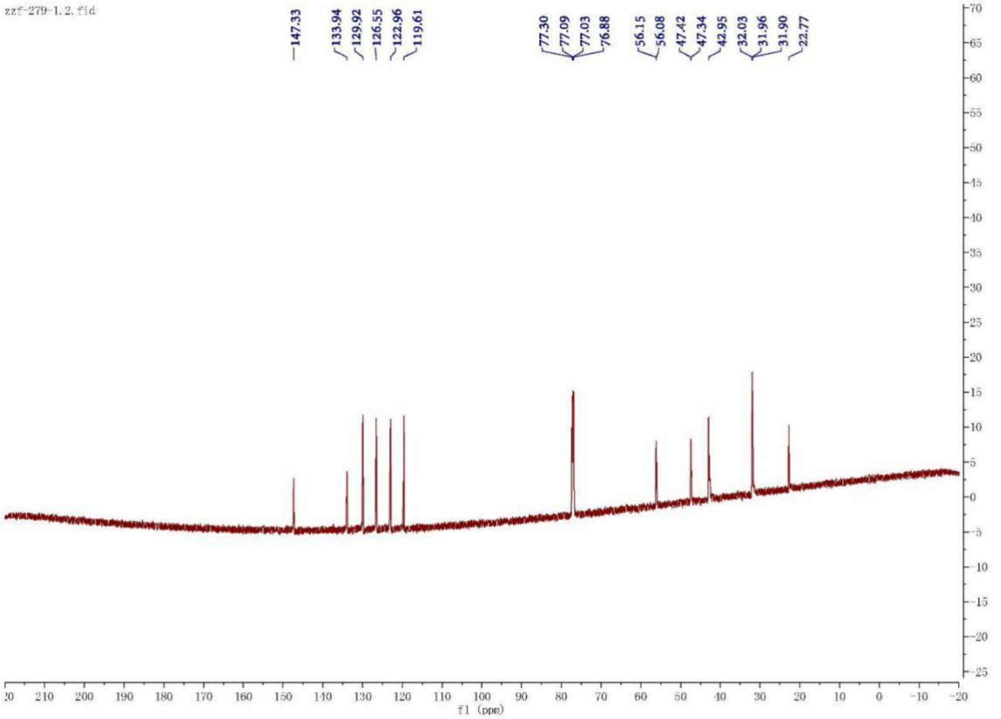

Supplementary Figure 132. ^{13}C NMR of product **4y**.

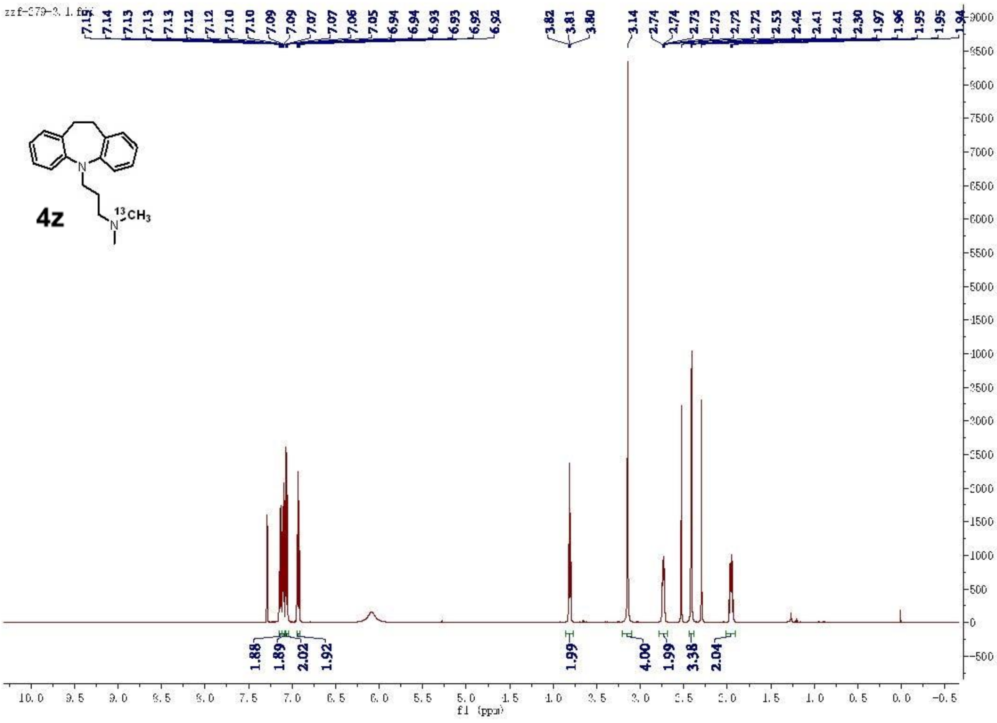

Supplementary Figure 133. ^{1}H NMR of product **4z**.

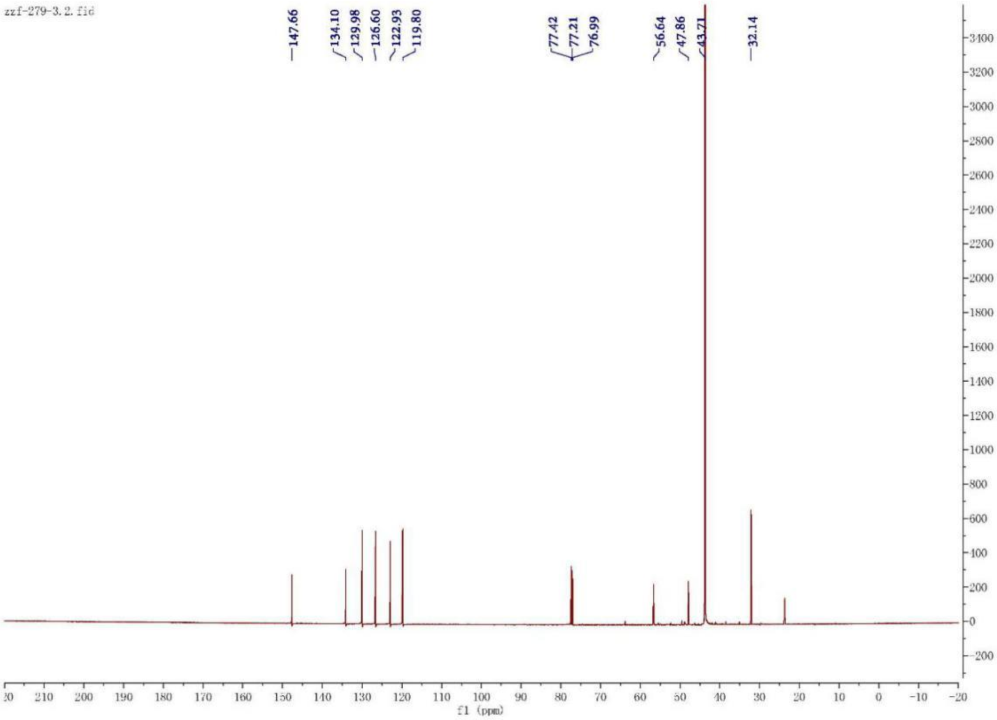

Supplementary Figure 134. ^{13}C NMR of product **4z**.

Supplementary References

1. Yu, R. P., Hesk, D., Rivera, N., Pelczer, I., Chirik, P. J. Iron-catalysed tritiation of pharmaceuticals. *Nature* **529**, 195-199 (2016).

2. Brown, J. A. et al. The synthesis of highly active Iridium(I) complexes and their application in catalytic hydrogen isotope exchange. *Adv. Synth. Catal*. **356**, 3551-3562 (2014).

3. Loh, Y. et al. Photoredox-catalyzed deuteration and tritiation of pharmaceutical products. *Science* **358**, 1182-1187 (2017).

4. Neubert, L. et al. Ruthenium-catalyzed selective α, β-deuteration of bioactive amines. *J. Am. Chem. Soc.* **134**, 12239-12244 (2012)

5. Shagufta et al. Exploring chemical substructures essential for hERG K+ channel blockade by synthesis and biological evaluation of Dofetilide Analogues. *ChemMedChem***, 4**, 1722-1732 (2009).

6. Rosen, B. R., Ruble, J. C., Beauchamp, T. J., Navarro, A. Mild Pd-Catalyzed N-Arylation of methanesulfonamide and related nucleophiles: Avoiding potentially genotoxic reagents and byproducts. *Org. Lett.* **13**, 2564-2567 (2011).
